# Supplementary material for: Wiring Between Close Nodes in Molecular Networks Evolves More Quickly Than Between Distant Nodes
Source: Mol Biol Evol. 2024 May 20;41(5):msae098. doi: 10.1093/molbev/msae098 (PMC11136681; doi:10.1093/molbev/msae098)

Supplementary Materials for

Wiring between close nodes in biological networks evolves more quickly than between distant nodes

Alejandro Gil-Gomez, alejandro.gilgomez@stonybrook.edu
Joshua S. Rest*, joshua.rest@stonybrook.edu, 631-632-1916
Department of Ecology and Evolution
Stony Brook University
650 Life Sciences
Stony Brook, NY 11794-4254

*Corresponding author

Sup. Table S1. Drug-target mapping
Sup. Table S2. Statistical summaries for Figure 4
- Statistical summary for Fig. 4A
- Statistical summary for Fig. 4B
- Statistical summary for Fig. 4C
- Statistical summary for Fig. 4D

Sup. Table S3. Details of some of the DDIs analyzed.
Sup. Table S4. Network measurements of DDIs
Sup. Document S1. t-SNE results of all DDIs for perplexity range between 5 and 2100.
Sup. Fig. S1: DDI-based Euclidean distances between strains scale as a function of phylogenetic distance plotted on a log scale.
Sup. Figure S2. Evolutionary rates as a function of minimum distance between targets for the STRING-DB networks. STRING-DB-full (.protein.links.detailed.v12.0.txt.gz) and STRING-DB-physical (*.protein.physical.links.detailed.v12.0.txt.gz) for the strains: Escherichia coli K-12 BW2952 (ebw), Escherichia coli O8 IAI1 (ecr), Pseudomonas aeruginosa PAO1 (pae), Salmonella enterica subsp. enterica serovar Typhimurium LT2 (stm).
Sup. Fig. S3: Evolutionary rates of DDIs vs network metrics of DDI targets in two E coli networks.
Sup. Fig. S4. Dotplot showing significant GO categories of drug targets across clusters. Clusters 3, 6, and 16 have significant enrichment for ion binding. Clusters 2, 3, 4, 6, and 16 are significantly enriched for anion binding and small molecule binding.
Sup. Fig. S5. Heatmap of DDI scores across strains for the subset of DDI that are susceptible in at least one of the species for both drugs.
Sup. Fig. S6. Differences in minimum path length, k edge connectivity, mean node degree, mean betweenness centrality, and mean eigenvector centrality between targets for different types of drug interactions in E. coli for co-functional and PPI networks for the subset of DDI that are susceptible in at least one of the species for both drugs.
Sup. Fig. S7. Relationships among evolutionary rate, interaction type, and path length for the subset of DDI that are susceptible in at least one of the species for both drugs.

Sup. Table S1. Drug-target mapping
See separate CSV File.

Sup. Table S2. Statistical summaries for Figure 4

**Statistical summary for Fig. 4A**					
**Group 1**	**Group 2**	**statistical test**	**Statistic**	**p-value**	**df**
"Additivity-Synergy"	"Additivity-Antagonism-Synergy"	Welch Two Sample t-test	3.134	0.002	128.7
"Additivity-Synergy"	"Additivity"	Welch Two Sample t-test	13.06	p < 0.001	417.249
"Additivity-Synergy"	"Additivity-Antagonism"	Welch Two Sample t-test	6.735	p < 0.001	486.877
"Additivity-Synergy"	"Synergy"	Welch Two Sample t-test	-24.847	p < 0.001	390
"Additivity-Synergy"	"Antagonism-Synergy"	Not enough observations	NA	NA	NA
"Additivity-Antagonism-Synergy"	"Additivity"	Welch Two Sample t-test	4.214	p < .001	73.759
"Additivity-Antagonism-Synergy"	"Additivity-Antagonism"	Welch Two Sample t-test	0.581	0.526	78.427
"Additivity-Antagonism-Synergy"	"Synergy"	Welch Two Sample t-test	-18.39	p < .001	72
"Additivity-Antagonism-Synergy"	"Antagonism-Synergy"	Not enough observations	NA	NA	NA
"Additivity"	"Additivity-Antagonism"	Welch Two Sample t-test	-15.407	p < .001	753.83
"Additivity"	"Synergy"	Welch Two Sample t-test	-205.33	p < .001	1707
"Additivity"	"Antagonism-Synergy"	Not enough observations	NA	NA	NA
"Additivity-Antagonism"	"Synergy"	Welch Two Sample t-test	-90.677	p < .001	472
"Additivity-Antagonism"	"Antagonism-Synergy"	Not enough observations	NA	NA	NA
"Synergy"	"Antagonism-Synergy"	Not enough observations	NA	NA	NA

**Statistical summary for Fig. 4B**					
**Group 1**	**Group 2**	**statistical test**	**Statistic**	**p-value**	**df**
Synergy	Additivity	Welch Two Sample t-test	18.902	p < .001	117.717
Synergy	Antagonism	Welch Two Sample t-test	-12.37	p < .001	95.955
Additivity	Antagonism	Welch Two Sample t-test	-16.896	p < .001	91.776

Sup. Table S2 (continued). Statistical summaries for Figure 4

Statistical summary for Fig. 4C

Group 1	Group 2	statistical test	Statistic	p-value	df	Network
Synergy	Additivity	Welch Two Sample t-test	48.985	p < .001	1542.953	Co-functional (EcoCyc/GO-BP)
Synergy	Antagonism	Welch Two Sample t-test	27.983	p < .001	226.056	Co-functional (EcoCyc/GO-BP)
Additivity	Antagonism	Welch Two Sample t-test	5.113	p < .001	188.331	Co-functional (EcoCyc/GO-BP)
Synergy	Additivity	Welch Two Sample t-test	53.463	p < .001	1233.005	Small/medium-scale PPI
Synergy	Antagonism	Welch Two Sample t-test	25.244	p < .001	224.621	Small/medium-scale PPI
Additivity	Antagonism	Welch Two Sample t-test	1.45	0.148	205.288	Small/medium-scale PPI

Statistical summary for Fig. 4D

Model	Term	Estimate	std.error	statistic	p-value	Network
OLS(Mean.sigma ~ path.length.corr)	Intercept	0.00207	0.000247	8.37	0.00111	Co-functional (EcoCyc/GO-BP)
OLS(Mean.sigma ~ path.length.corr)	Slope	-0.00024	0.000063	-3.75	0.01992	Co-functional (EcoCyc/GO-BP)
OLS(Mean.sigma ~ path.length.corr)	Intercept	0.00278	0.000212	13.15	0.00019	Small/medium-scale PPI
OLS(Mean.sigma ~ path.length.corr)	Slope	-0.00027	0.000054	-4.96	0.00769	Small/medium-scale PPI

Sup. Table S3. Details of some of the DDIs analyzed.

**Drug pair**	**A22_Novobiocin**	**A22_Novobiocin**	**Erythromycin_Trimethoprim**	**Sulfamonomethoxine_Trimethoprim**
**Network**	Co-functional EcoCyc/GO-BP	Small/medium scale PPI	NA	Co-functional EcoCyc/GO-BP
**Sigma rate** (bliss^2/MYA)	0.0035	0.0035	0.0003	0.0035
**Path length**	2	3.5	NA	1
**K-edge**	12	3	NA	21
**Mean node degree**	16	5.75	NA	26.5
**DDI in ebw (D1-D2)**	Additive (S/S)		Additive (S/S)	Synergy **(R/S)**
**DDI in ecr (D1-D2)**	Synergy (S/S)		Additive (S/S)	Synergy **(R/S)**
**DDI in seo (D1-D2)**	Synergy **(S/R)**		Additive (S/**R**)	Synergy **(R/S)**
**DDI in stm (D1-D2)**	Additive (S/S)		Additive (S/**R**)	Synergy **(R/S)**
**DDI in pae (D1-D2)**	Additive **(R/R)**		Additive (S/S)	Additive **(R/R)**
**DDI in pau (D1-D2)**	Additive **(S/R)**		Additive (S/S)	Additive **(R/R)**

Sup. Table S4. Network measurements of DDIs
See separate CSV File.

Sup. Document S1. t-SNE results of all DDIs for perplexity range between 5 and 2100.
See separate PDF File

Sup. Fig. S1: DDI-based Euclidean distances between strains scale as a function of phylogenetic distance plotted on a log scale.

Intraspecific comparisons (in blue) have the lowest amount of sequence and chemical divergence, while comparisons with Pseudomonas are the most distant in terms of sequence and chemical divergence. All drug-drug interaction data is from Brochado et al. (2018).

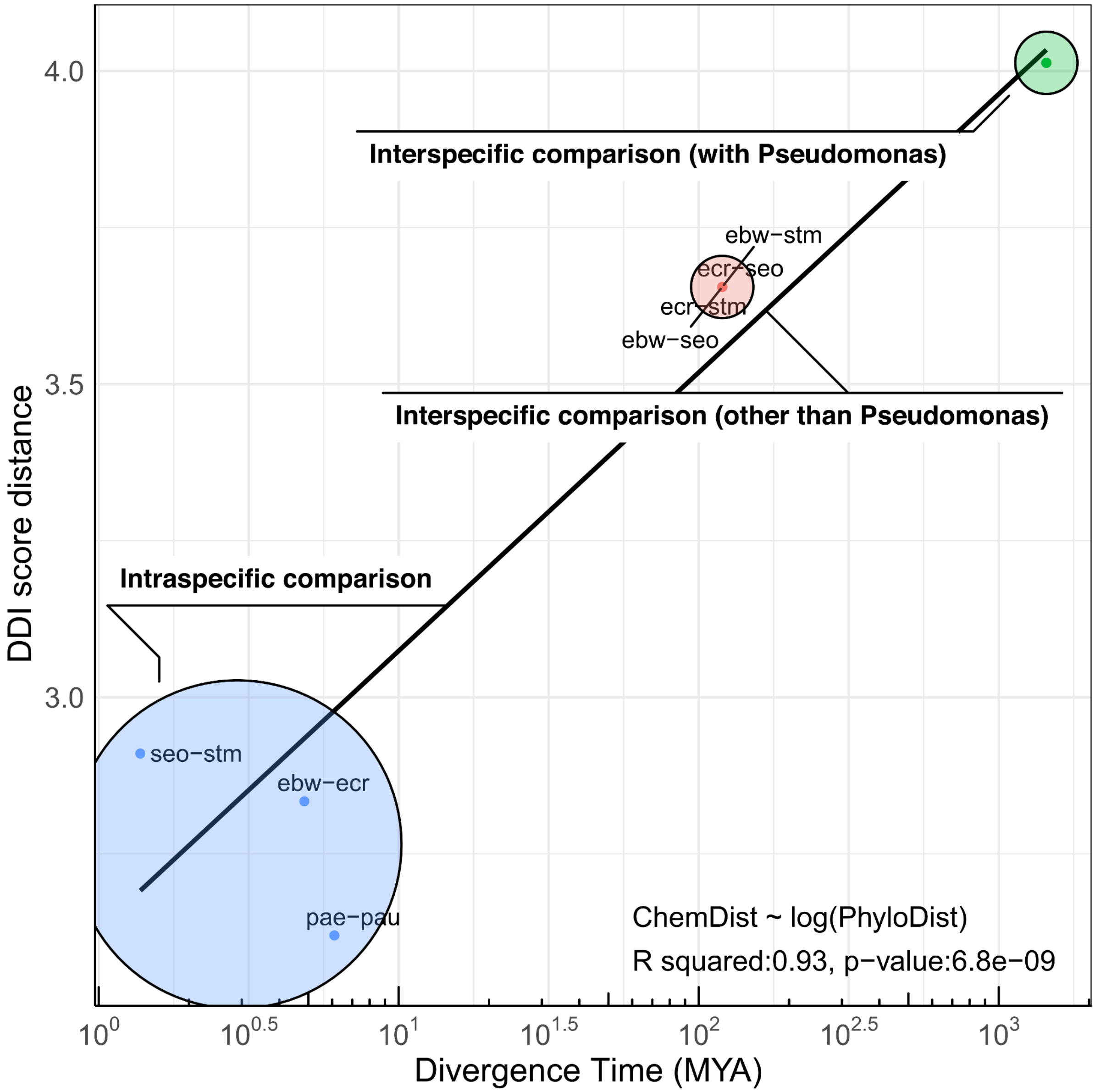

Sup. Figure S2. Evolutionary rates as a function of minimum distance between targets for comparative STRING networks. STRING-DB-full (*.protein.links.detailed.v12.0.txt.gz) and STRING-DB-physical (.protein.physical.links.detailed.v12.0.txt.gz) for the strains: *Escherichia coli* K-12 BW2952 (ebw), *Escherichia coli* O8 IAI1 (ecr), *Pseudomonas aeruginosa* PAO1 (pae), *Salmonella enterica* subsp. enterica serovar Typhimurium LT2 (stm).

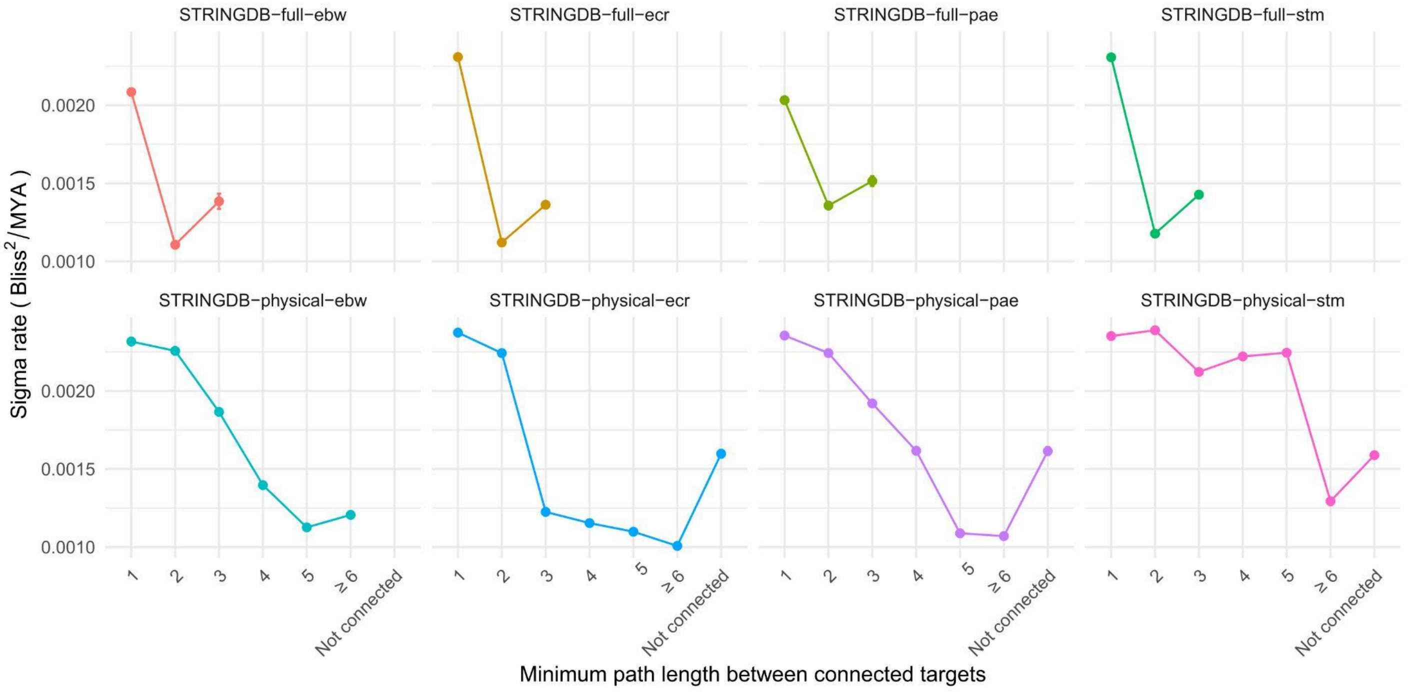

Sup. Fig. S3: Evolutionary rates of DDIs vs network metrics of DDI targets in two *E coli* networks.

A. Comparison of evolutionary rates between adjacent and non-adjacent targets in the co-functional (EcoCyc) network.
B. Comparison of evolutionary rates between adjacent and non-adjacent targets in the PPI network.
C. Comparison of evolutionary rates between connected and not-connected targets in the co-functional (EcoCyc) network.
D. Comparison of the minimum distance between targets for different combinations of DDI across strains for co-functional (EcoCyc/GO-BP) and PPI networks.
E. Comparison of k edge connectivity between targets for different combinations of DDI across strains for (EcoCyc/GO-BP) and PPI networks.
F. Comparison of node degree between targets for different combinations of DDI across strains for (EcoCyc/GO-BP) and PPI networks.
G and **H.** Comparison of average eigenvector centrality between targets for different combinations of DDI across strains for (EcoCyc/GO-BP) and PPI networks.
I. and J. Distribution of connected and disconnected target connections for different types of DDI in the (EcoCyc/GO-BP) and PPI networks.
K. Comparison of evolutionary rates for different K-edge connectivity levels in the co-functional (EcoCyc) network.
L. Comparison of evolutionary rates for different node degree levels in the co-functional (EcoCyc) network.
M. Comparison of evolutionary rates for different betweenness centrality levels in the co-functional (EcoCyc) network.
N. Comparison of evolutionary rates for different eigenvector centrality levels in the co-functional (EcoCyc) network.

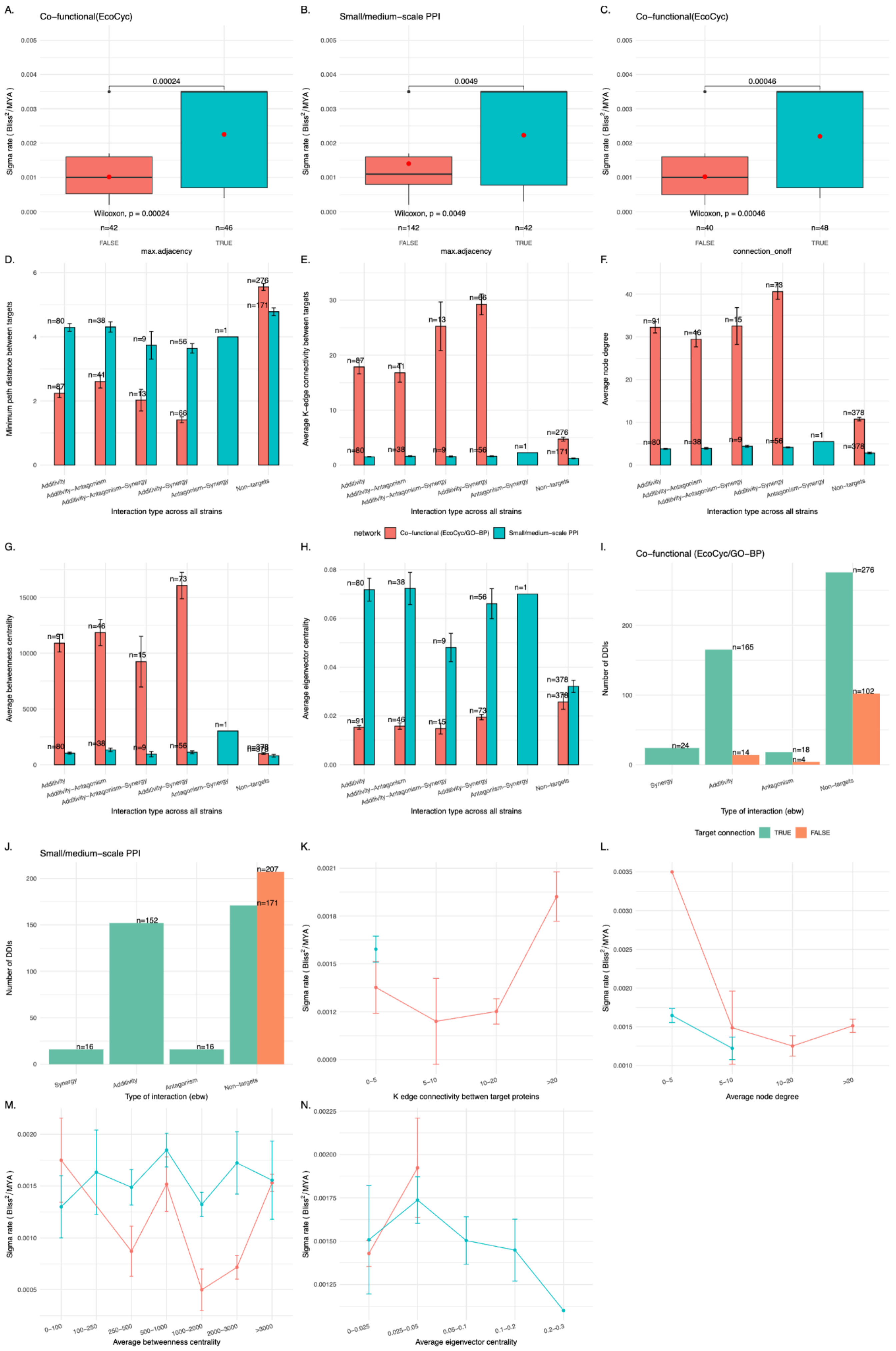

A.
Co−functional(EcoCyc)
Sigma rate (Bliss2 / MYA)
0.00024
Wilcoxon, p = 0.00024
n=42
n=46
FALSE
TRUE
max.adjacency
B.
Small/medium−scale PPI
Sigma rate (Bliss2 / MYA)
0.0049
Wilcoxon, p = 0.0049
n=142
n=42
FALSE
TRUE
max.adjacency
C.
Co−functional(EcoCyc)
Sigma rate (Bliss2 / MYA)
0.00046
Wilcoxon, p = 0.00046
n=40
n=48
FALSE
TRUE
connection_onoff
D.
Minimum path distance between targets
Interaction type across all strains
E.
Average K−edge connectivity between targets
Interaction type across all strains
F.
Average node degree
Interaction type across all strains
Additivity
Additivity−Antagonism
Additivity−Antagonism−Synergy
Additivity−Synergy
Antagonism−Synergy
Non−targets
network
Co−functional (EcoCyc/GO−BP)
Small/medium−scale PPI
G.
Average betweenness centrality
Interaction type across all strains
H.
Average eigenvector centrality
Interaction type across all strains
I.
Co−functional (EcoCyc/GO−BP)
Number of DDIs
Synergy
Additivity
Antagonism
Non−targets
Type of interaction (ebw)
Target connection
TRUE
FALSE
J.
Small/medium−scale PPI
Number of DDIs
Synergy
Additivity
Antagonism
Non−targets
Type of interaction (ebw)
K.
Sigma rate (Bliss2 / MYA)
0−5
5−10
10−20
>20
K edge connectivity bettwen target proteins
L.
Sigma rate (Bliss2 / MYA)
0−5
5−10
10−20
>20
Average node degree
M.
Sigma rate (Bliss2 / MYA)
0−100
100−250
250−500
500−1000
1000−2000
2000−3000
>3000
Average betweenness centrality
N.
Sigma rate (Bliss2 / MYA)
0−0.025
0.025−0.05
0.05−0.1
0.1−0.2
0.2−0.3
Average eigenvector centrality

Sup. Fig. S4. Dotplot showing significant GO categories of drug targets across clusters. Clusters 3, 6, and 16 have significant enrichment for ion binding. Clusters 2, 3, 4, 6, and 16 are significantly enriched for anion binding and small molecule binding.

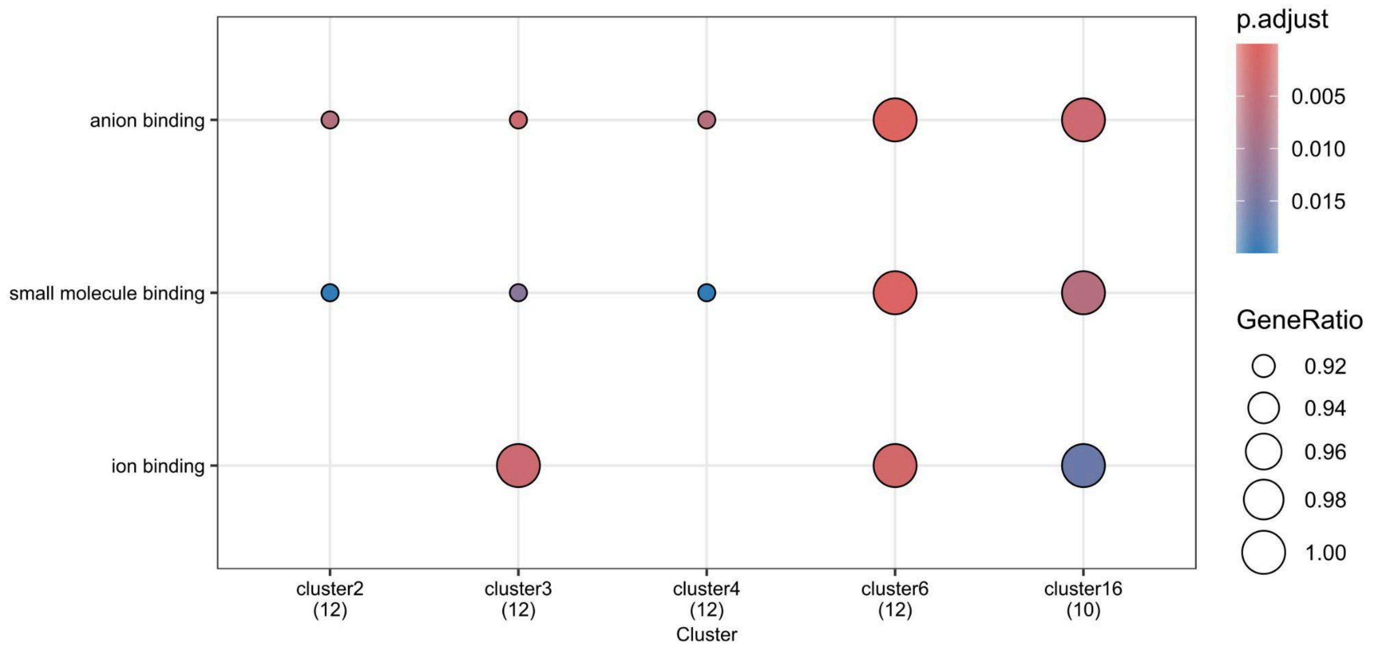

Sup. Fig. S5. Heatmap of DDI scores across strains for the subset of DDI that are susceptible in at least one of the species for both drugs.
Hierarchical clustering based on Euclidean distances across columns (strains: *E. coli* K-12 BW2952 (ebw), *Escherichia coli* O8 IAI1 (ecr), *S. enterica subsp. enterica* serovar Typhimurium 14028S (seo), *Salmonella enterica* subsp. enterica serovar Typhimurium LT2 (stm), *Pseudomonas aeruginosa* PAO1 (pae), *P. aeruginosa* UCBPP-PA14 (pau)), and rows (DDIs); the latter was constrained by tSNE clusters. Synergistic interactions are shown in blue and antagonistic interactions are in red, all DDIs are measured in bliss score units. The annotations on the right indicate whether the two drugs involved in the interaction belong to the same drug category, target the same cellular process, or have the same use (in green) or not (black). The bottom row indicates the evolutionary rate calculated in each cluster, measured in bliss score units^2 per million years.

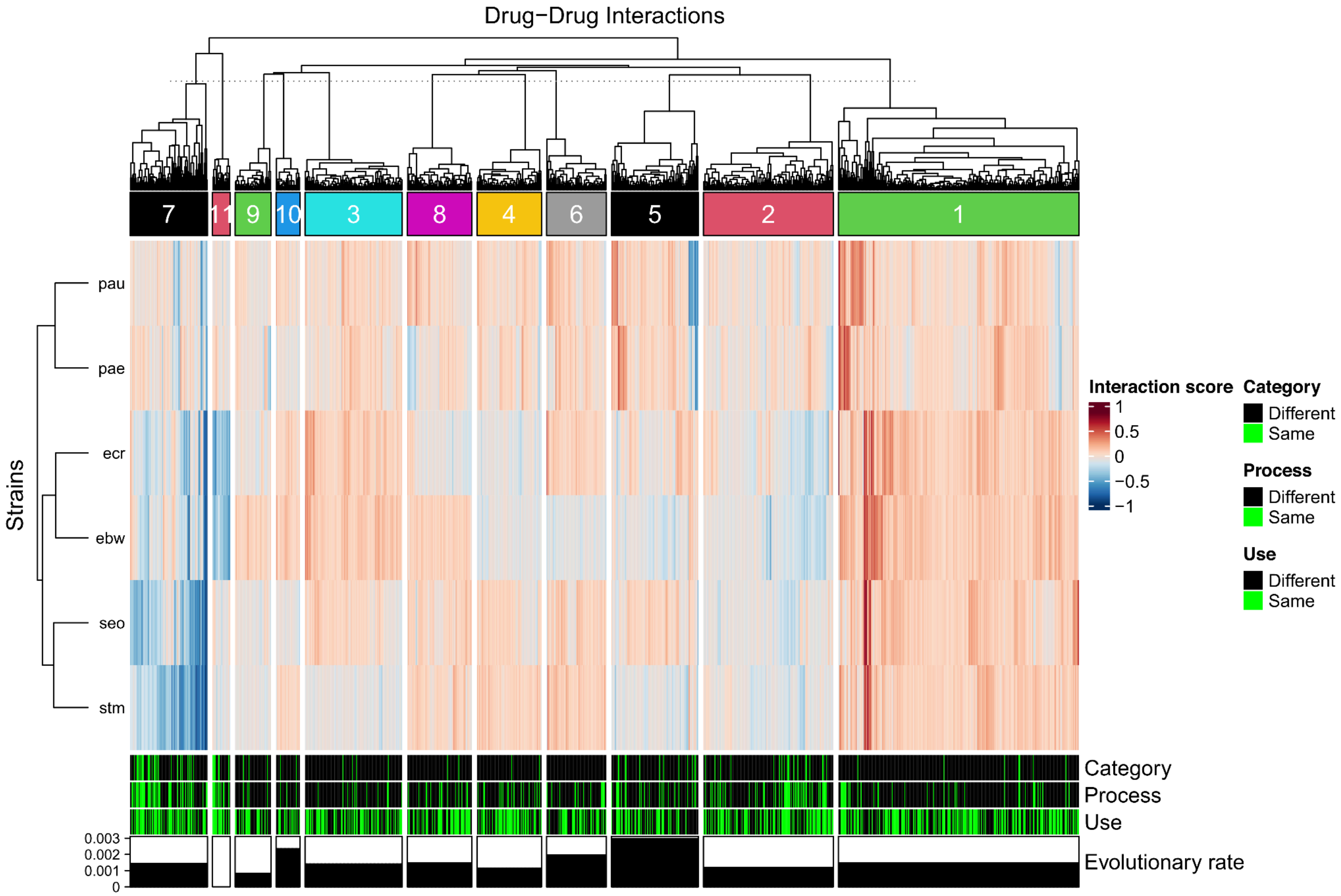

Sup. Fig. S6. Differences in minimum path length, k edge connectivity, mean node degree, mean betweenness centrality, and mean eigenvector centrality between targets for different types of drug interactions in *E. coli* for co-functional and PPI networks for the subset of DDI that are susceptible in at least one of the species for both drugs.

A-B. Synergistic interactions have a lower average path length than additive interactions and antagonistic interactions. **C-D.** K-edge connectivity is higher in synergies than in additive and antagonistic interactions in the co-functional network, but not in the PPI network. **E-F.** The mean node degree is higher in synergies than in additive or antagonistic interactions in the co-functional network, but not in the PPI network. **G-H**. Mean betweenness centrality is higher in synergies than in additive or antagonistic interactions in the co-functional network, but not in the PPI network. **I-J**. Mean eigenvector centrality is higher in synergies than in additive or antagonistic interactions in the co-functional network, but not in the PPI network.

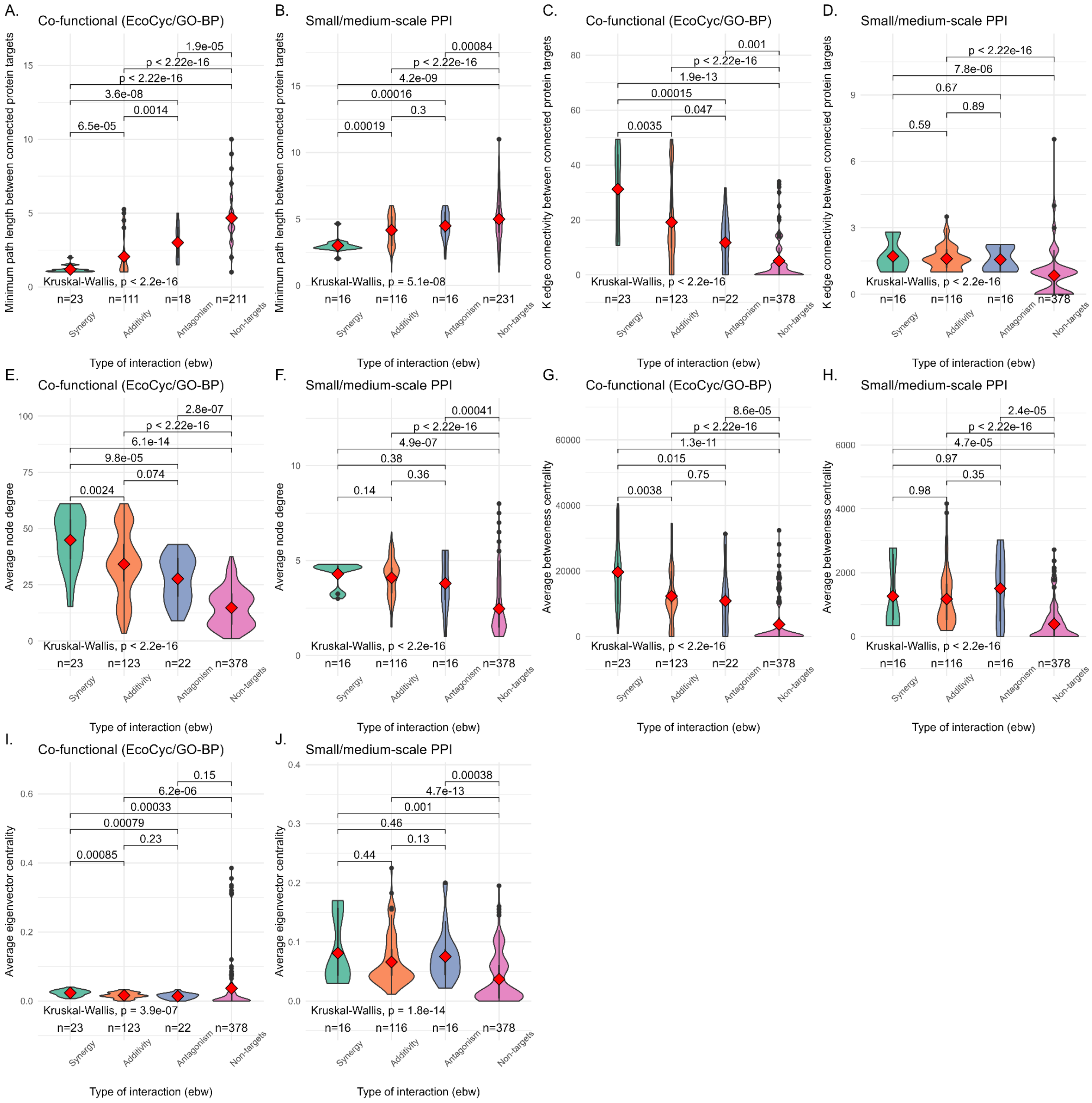

Sup. Fig. S7. Relationships among evolutionary rate, interaction type, and path length for the subset of DDI that are susceptible in at least one of the species for both drugs.

A. DDIs containing only synergies or synergy-antagonism combinations across all strains have faster evolutionary rates. Error bars represent the standard error of the mean. **B.** DDIs that are more synergistic across all strains have higher evolutionary rates. The score is the aggregated sum across strains, where DDIs were scored as +1 for synergies, 0 for additive, and -1 for antagonistic interactions. Error bars represent the standard error of the mean. **C.** Evolutionary rate as a function of interaction type for the co-functional (pink) and small/medium scale PPI network (blue). Synergistic interactions have higher evolutionary rates than additive and antagonistic interactions in *E. coli*. **D.** Evolutionary rate as a function of the minimum distance between targets. Wiring between close nodes in biological networks evolves more quickly than between distant nodes.

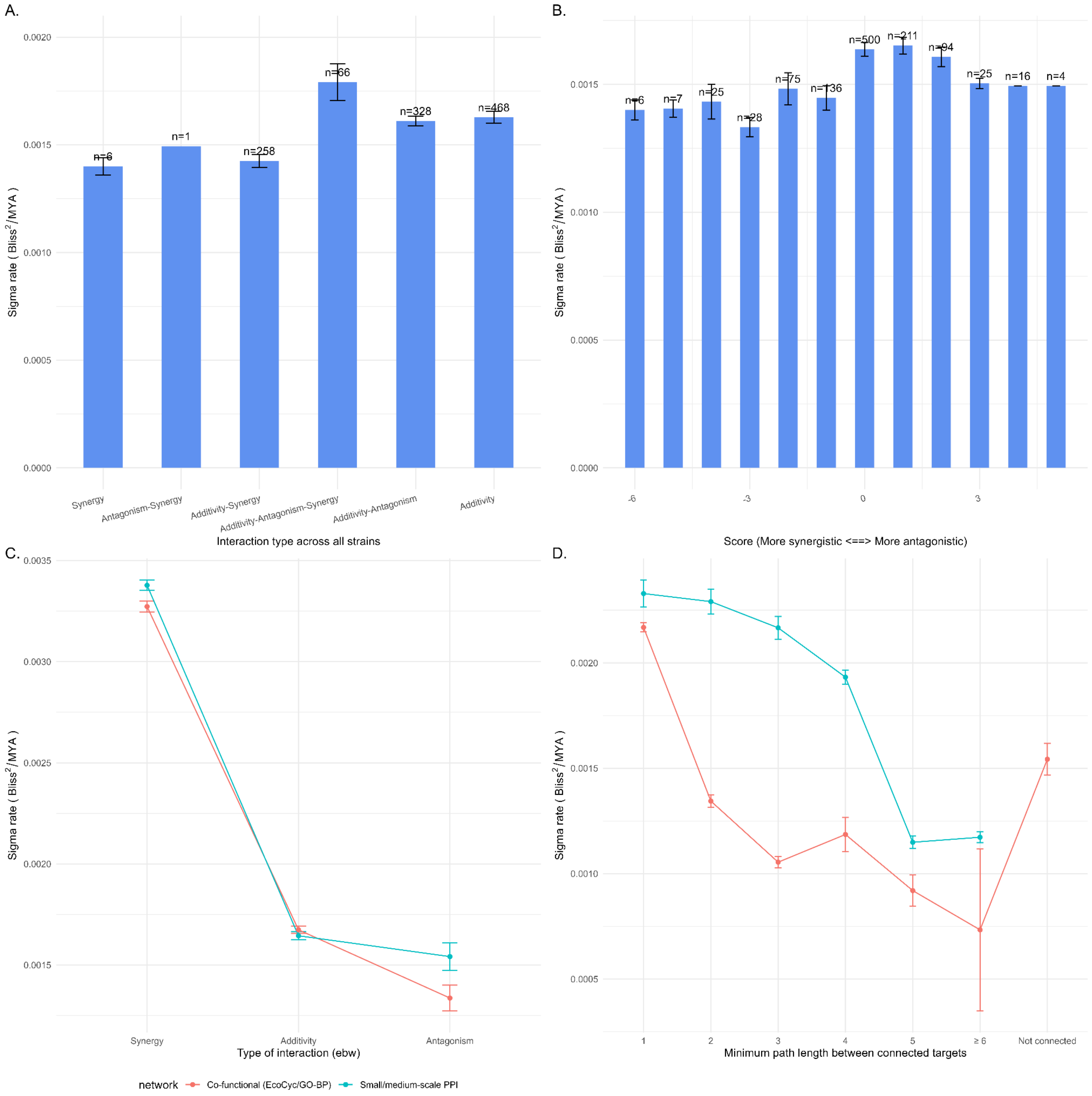

Supplement: msae098_Supplementary_Data [file msae098_supplementary_data.zip › GilGomez DDI-Supplementary Materials V2.pdf]
