# Supplementary material for: Wiring Between Close Nodes in Molecular Networks Evolves More Quickly Than Between Distant Nodes
Source: Mol Biol Evol. 2024 May 20;41(5):msae098. doi: 10.1093/molbev/msae098 (PMC11136681; doi:10.1093/molbev/msae098)
Supplement: msae098_Supplementary_Data [file msae098_supplementary_data.zip › SupDoc_S1-tSNE_results_largerange.pdf]

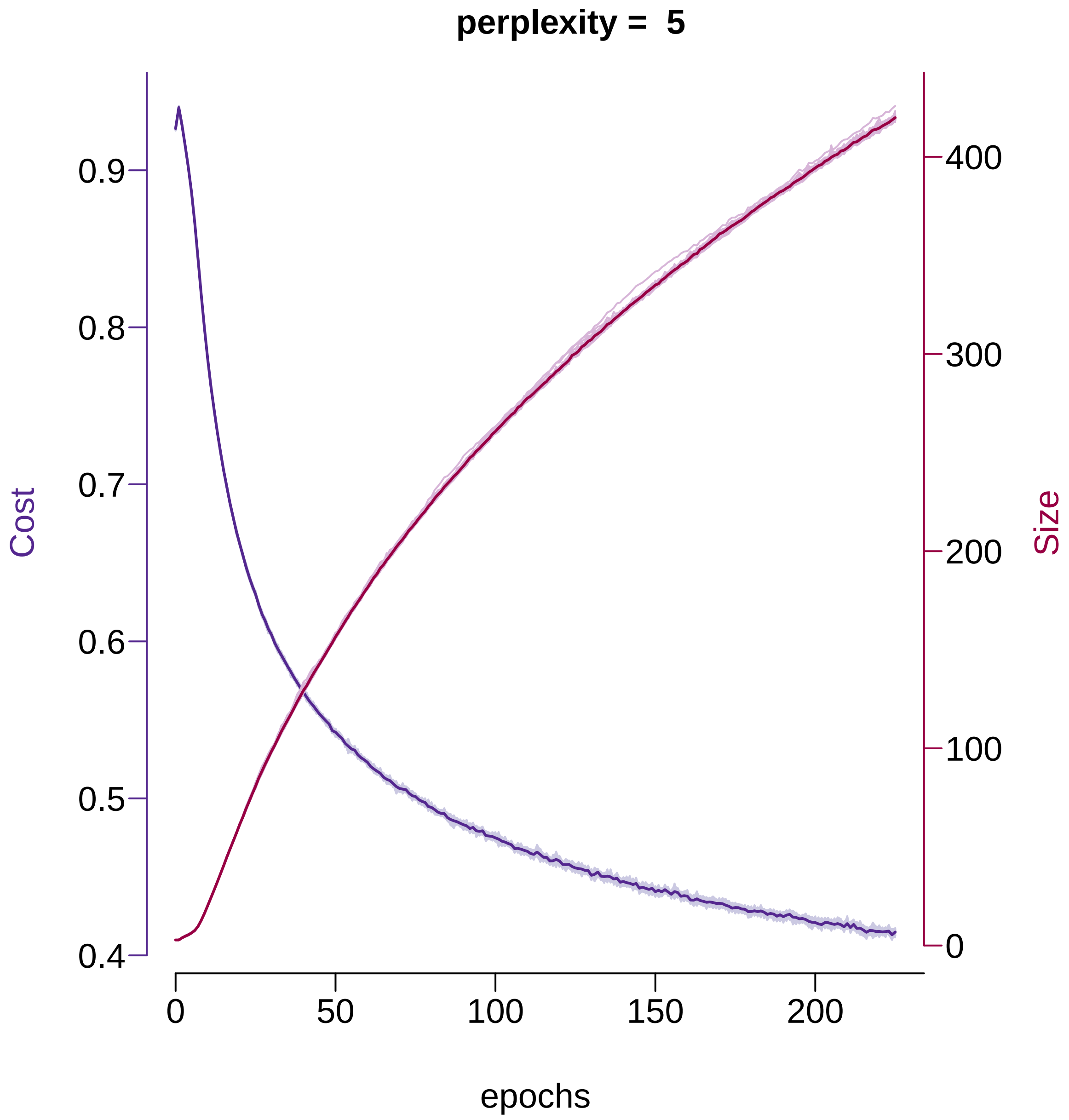
perplexity = 5
Cost
Size
epochs
0.9
0.8
0.7
0.6
0.5
0.4
400
300
200
100
0
0
50
100
150
200

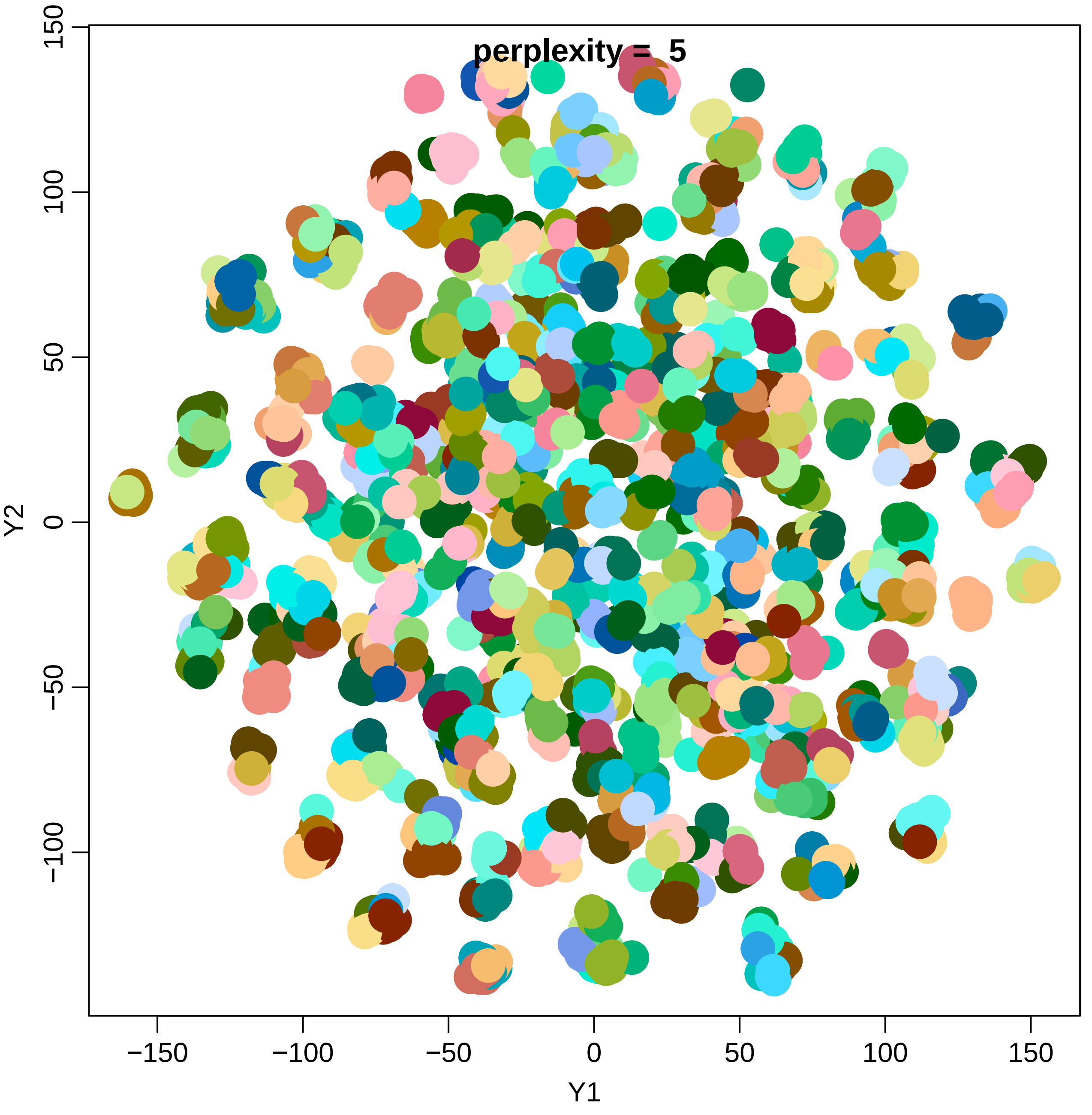

perplexity = 5
Y2
Y1
150
100
50
0
−50
−100
−150
−100
−50
0
50
100
150

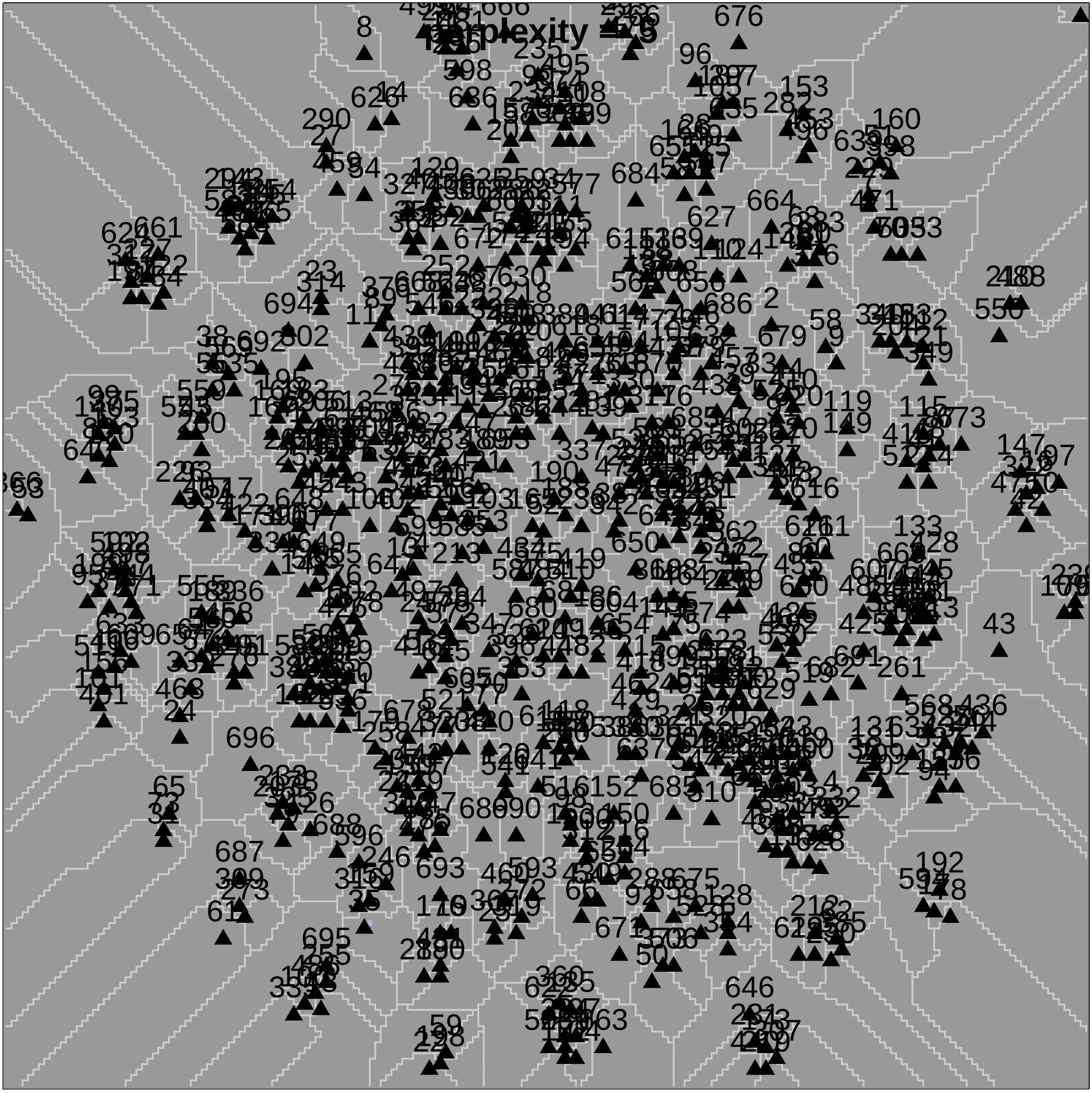

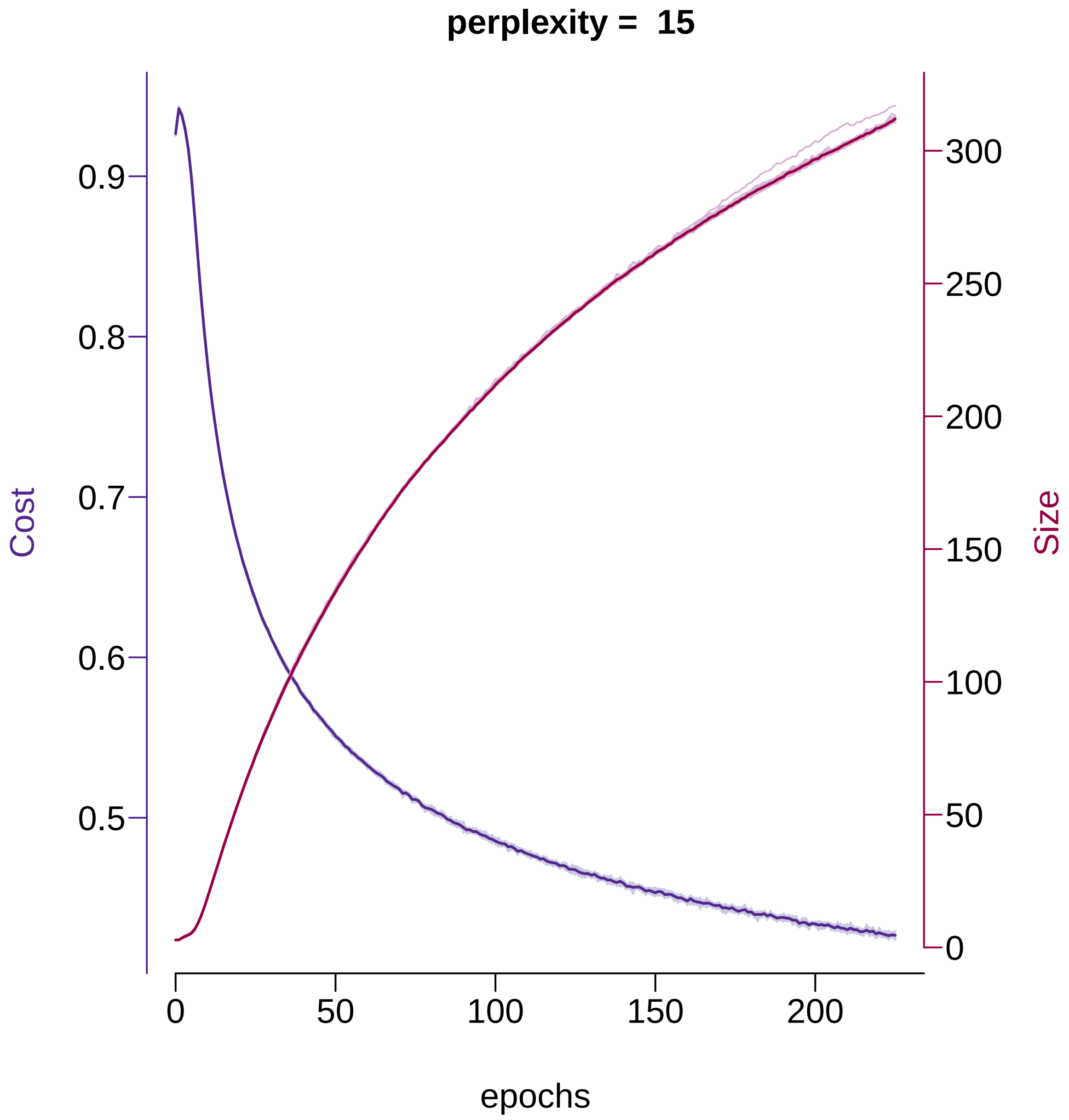
perplexity = 15
Cost
Size
epochs
0.9
0.8
0.7
0.6
0.5
300
250
200
150
100
50
0
0
50
100
150
200

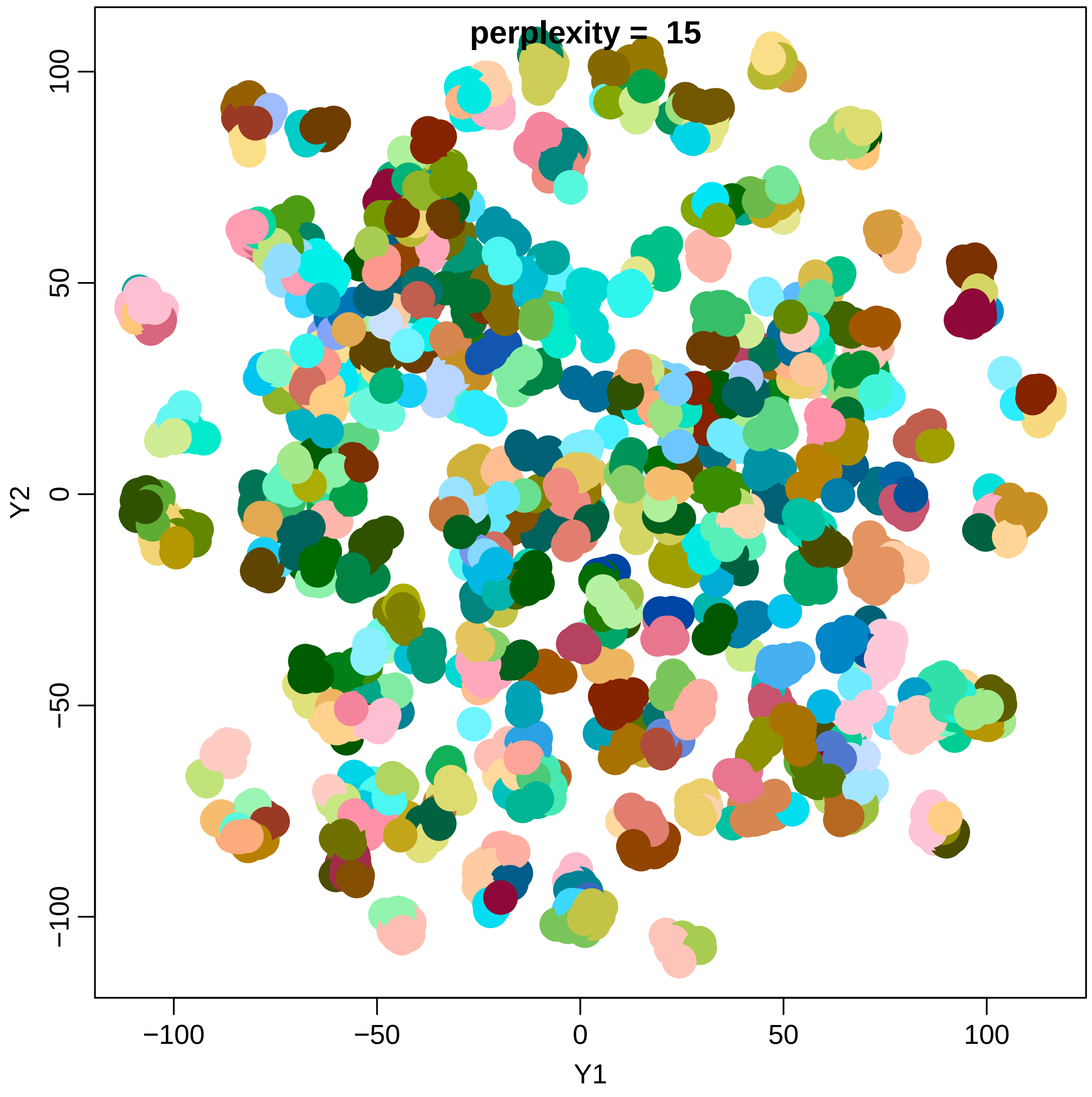
perplexity = 15
Y2
100
50
0
−50
−100
Y1
−100
−50
0
50
100

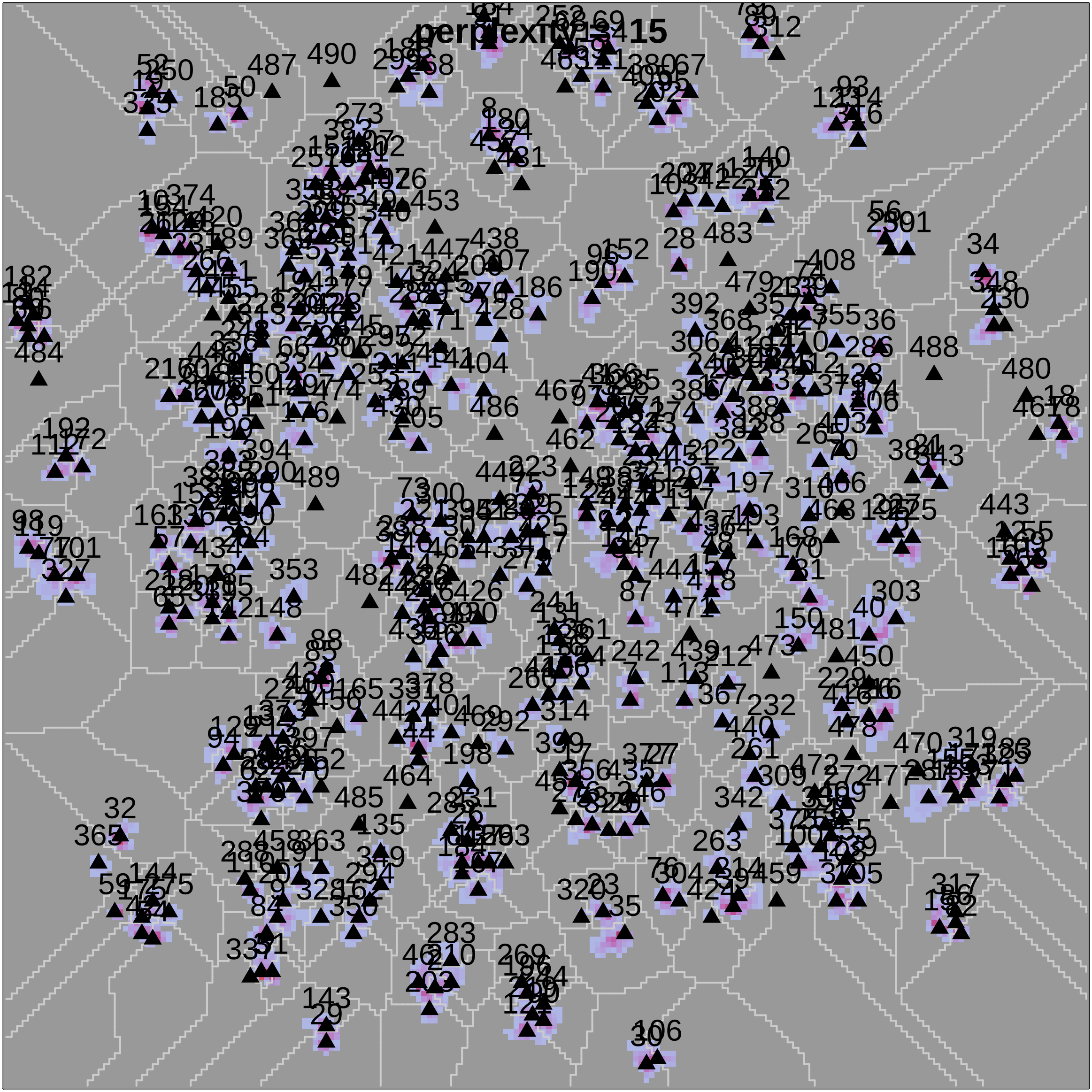
perplexity=15

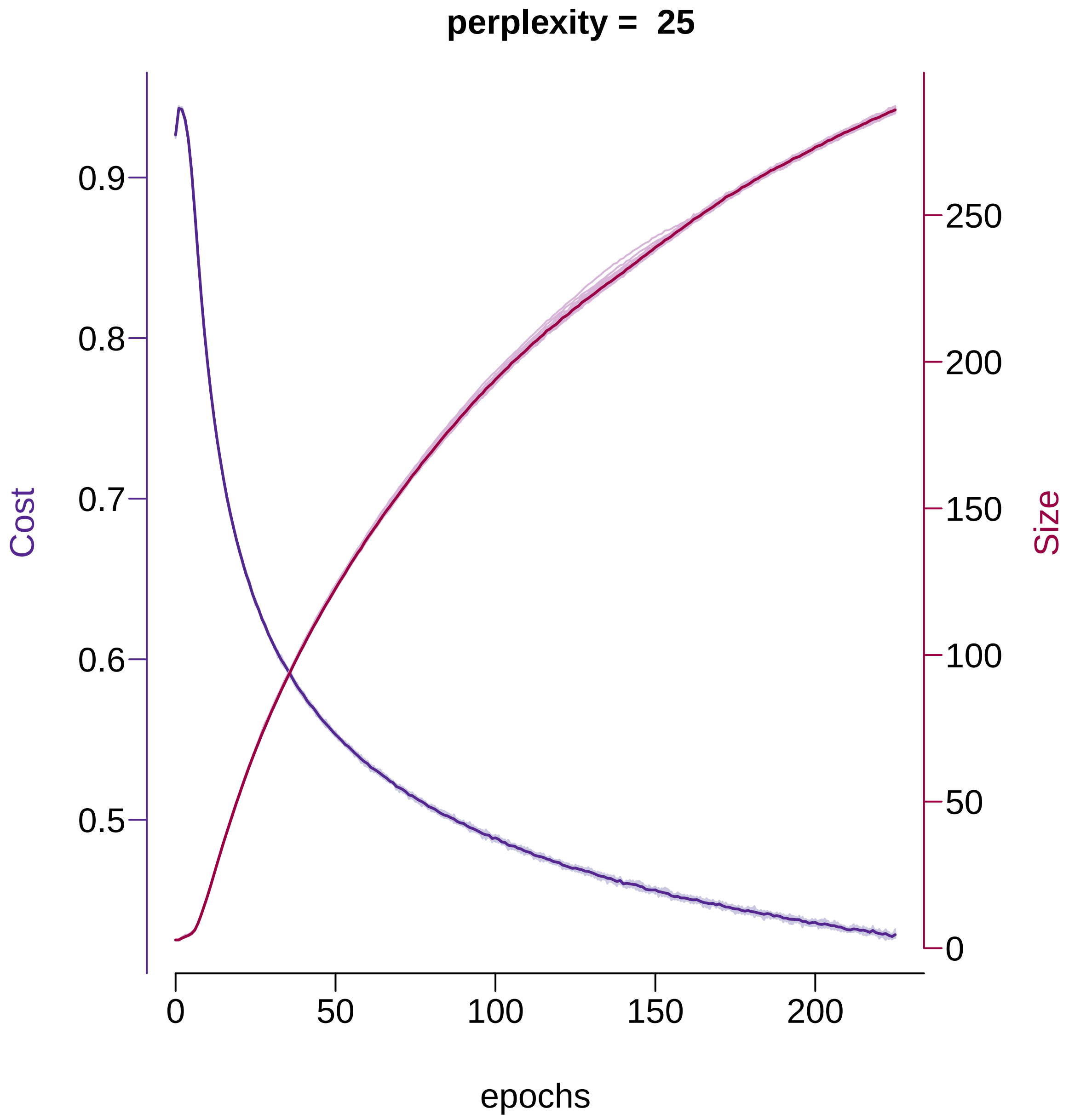
perplexity = 25
Cost
0.9
0.8
0.7
0.6
0.5
Size
250
200
150
100
50
0
0
50
100
150
200
epochs

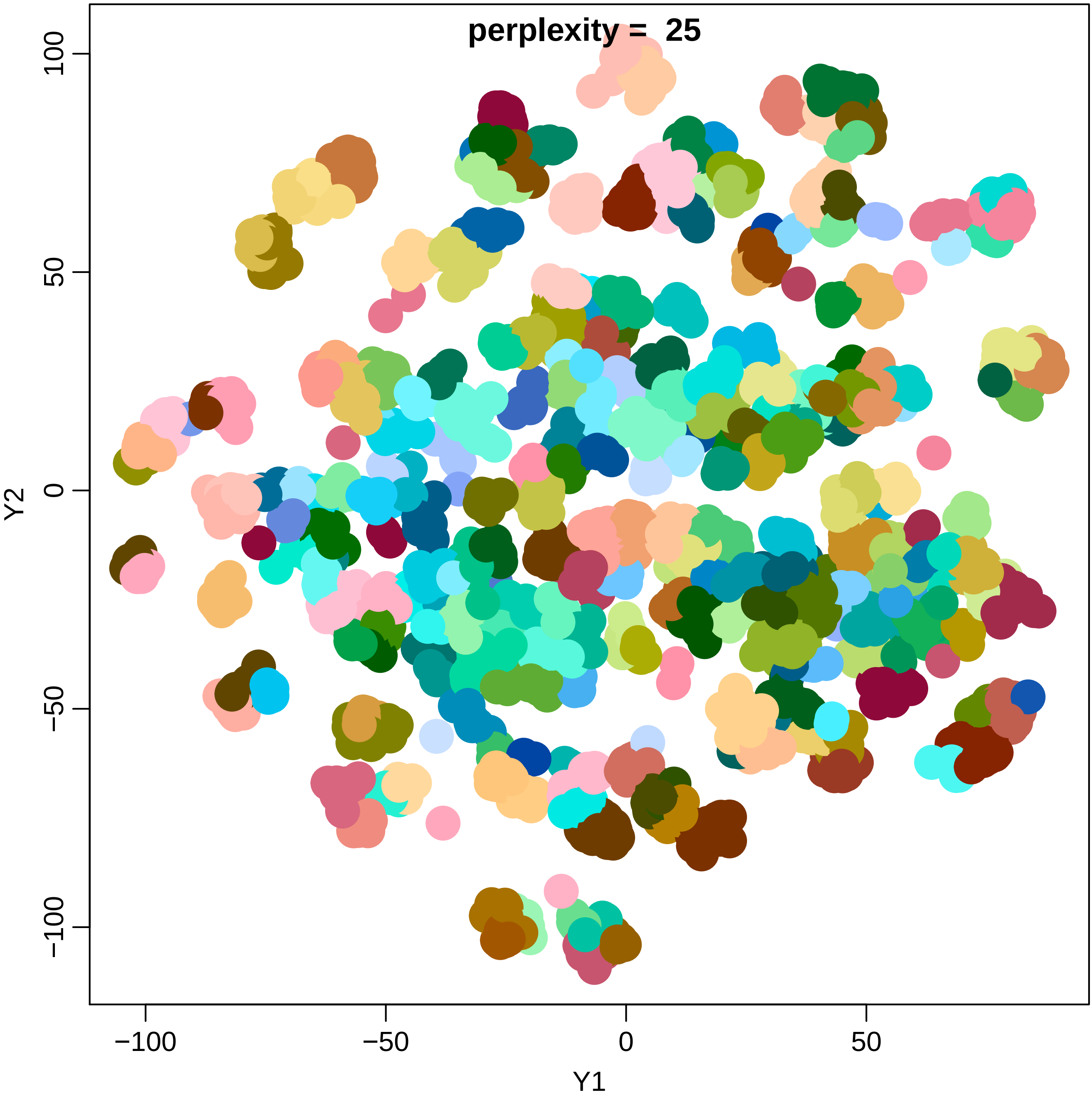
perplexity = 25
Y2
Y1
100
50
0
−50
−100
−100
−50
0
50

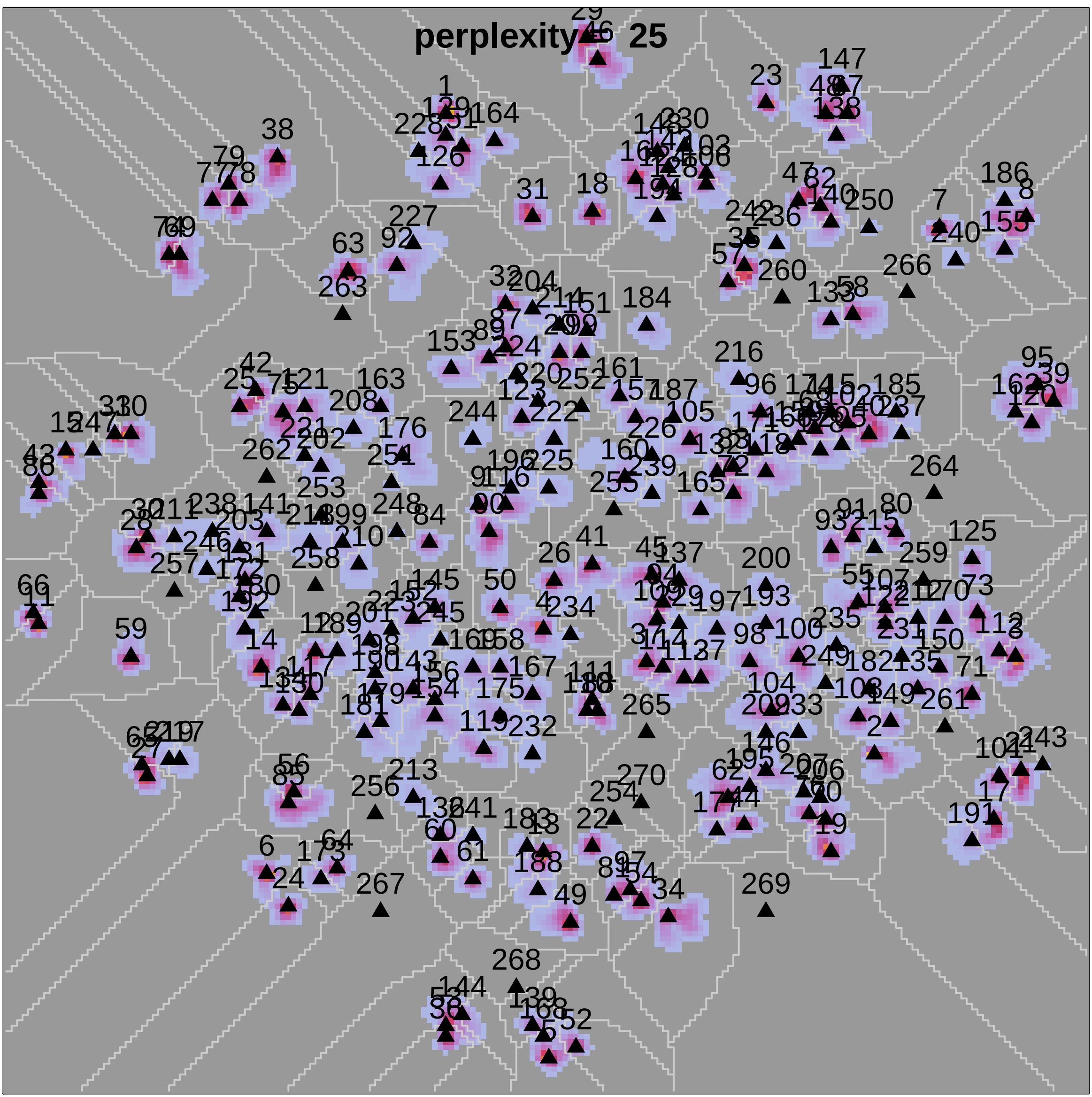
perplexity = 25

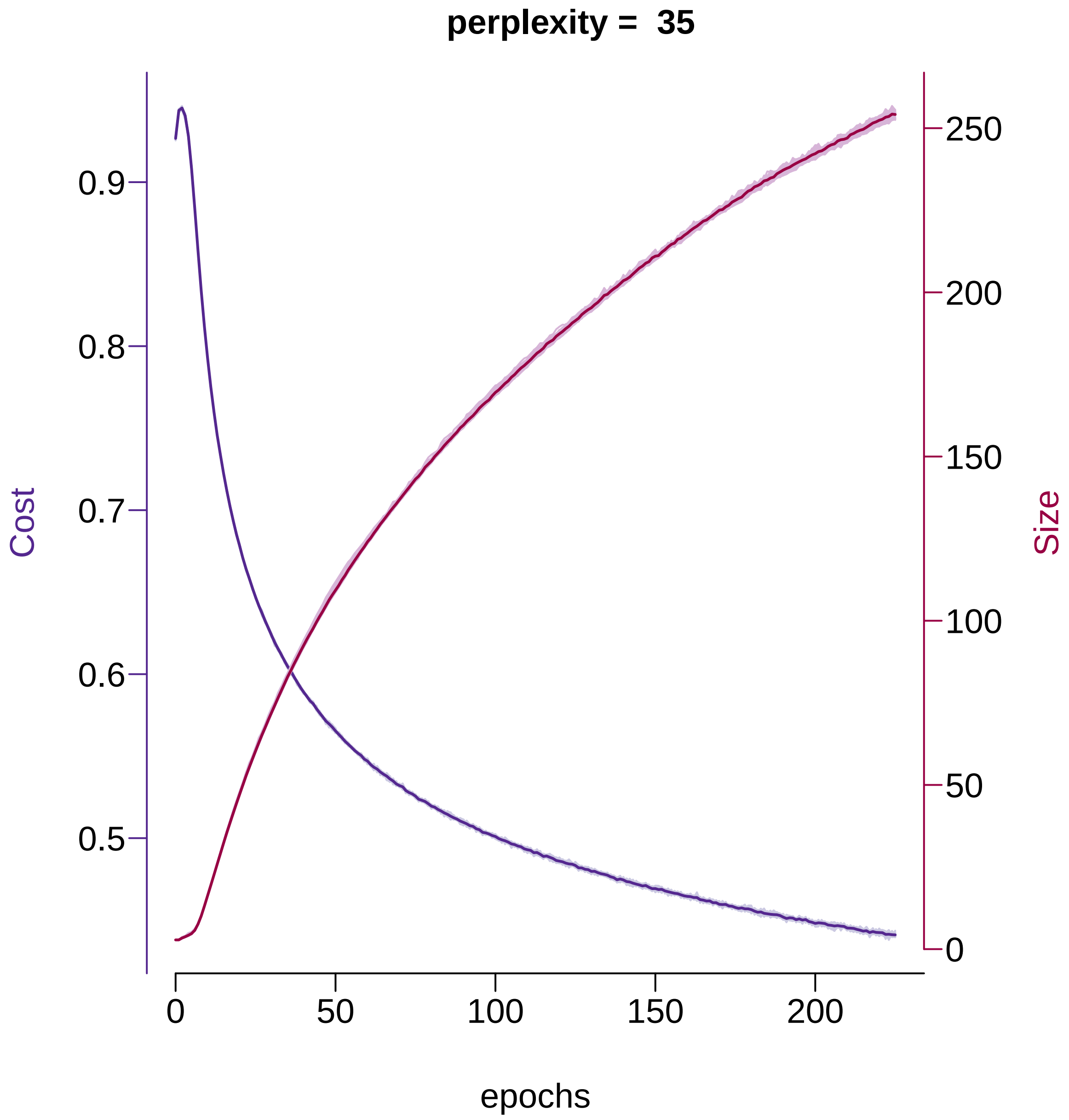
perplexity = 35
Cost
0.9
0.8
0.7
0.6
0.5
Size
250
200
150
100
50
0
0
50
100
150
200
epochs

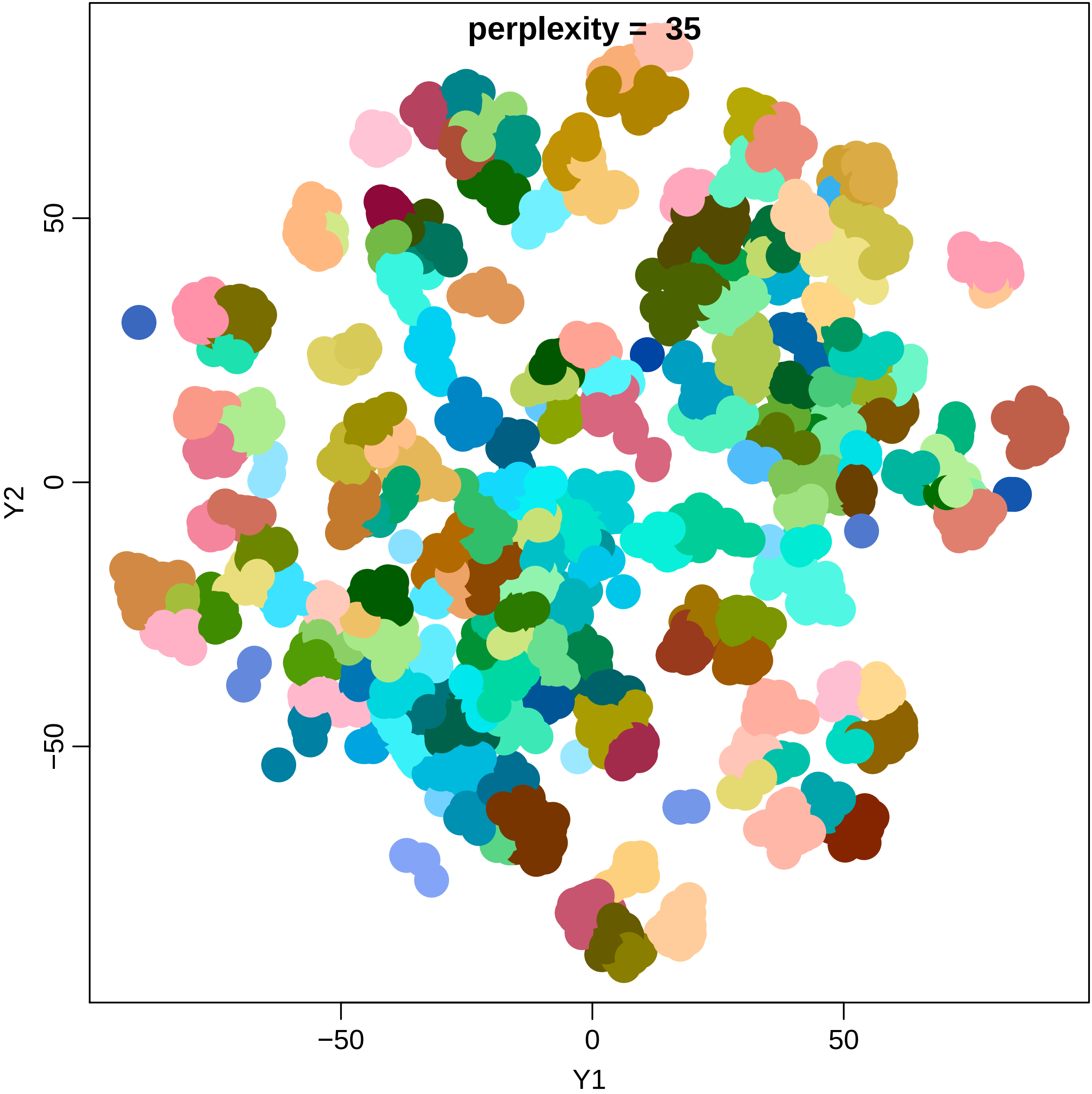
perplexity = 35
Y2
Y1
50
0
−50
−50
0
50

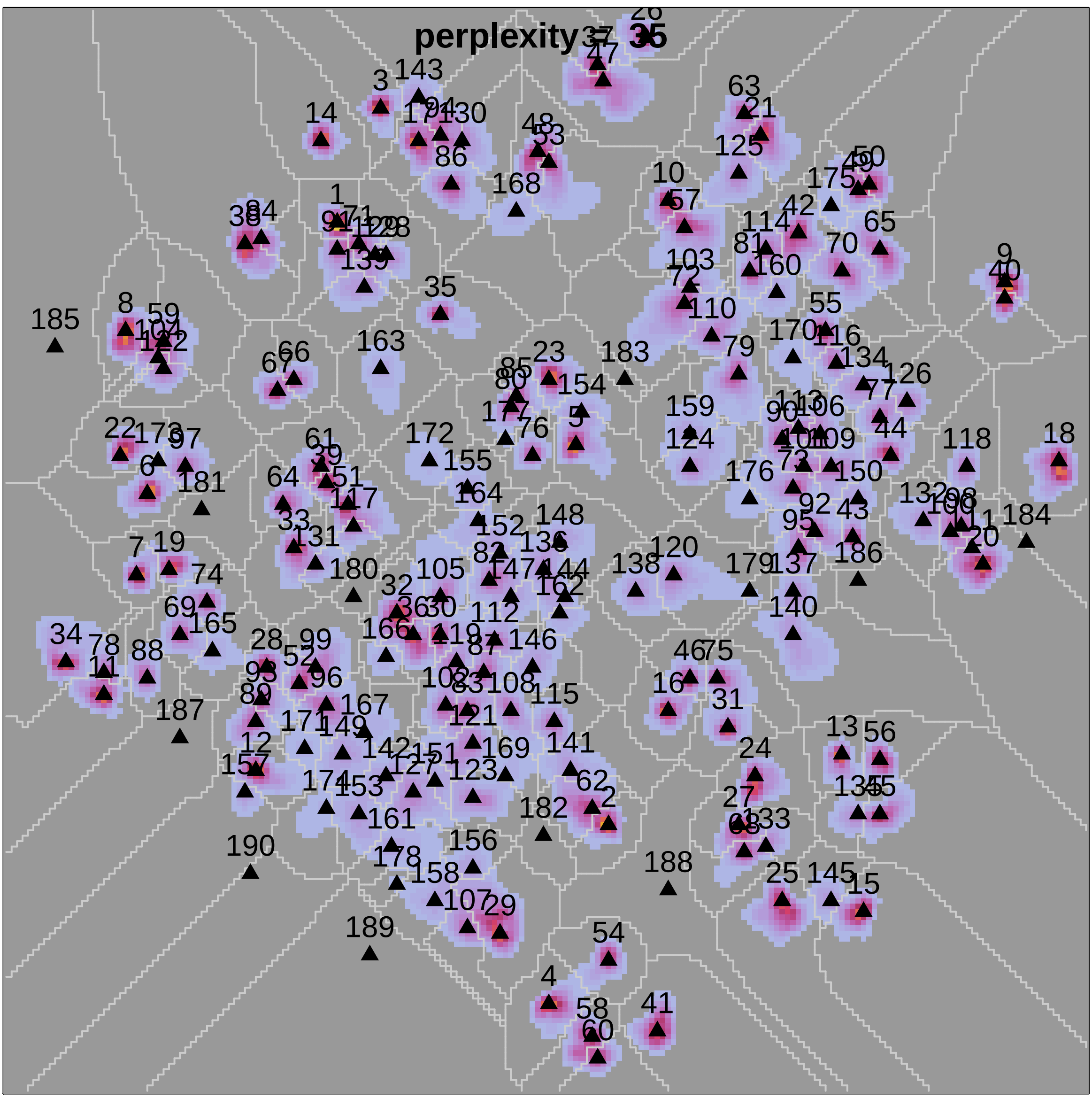
perplexity = 35

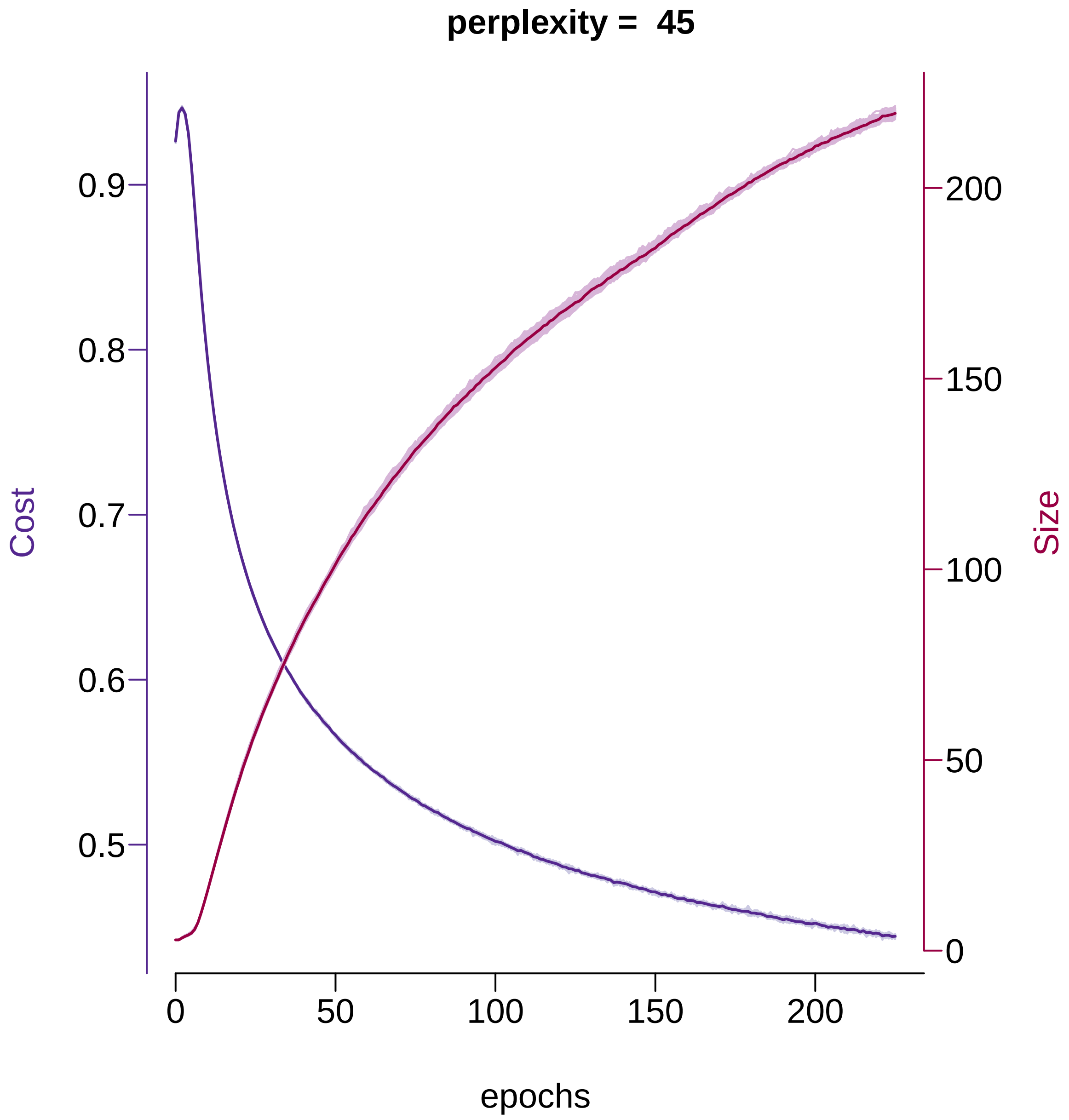
perplexity = 45
Cost
0.9
0.8
0.7
0.6
0.5
Size
200
150
100
50
0
0
50
100
150
200
epochs

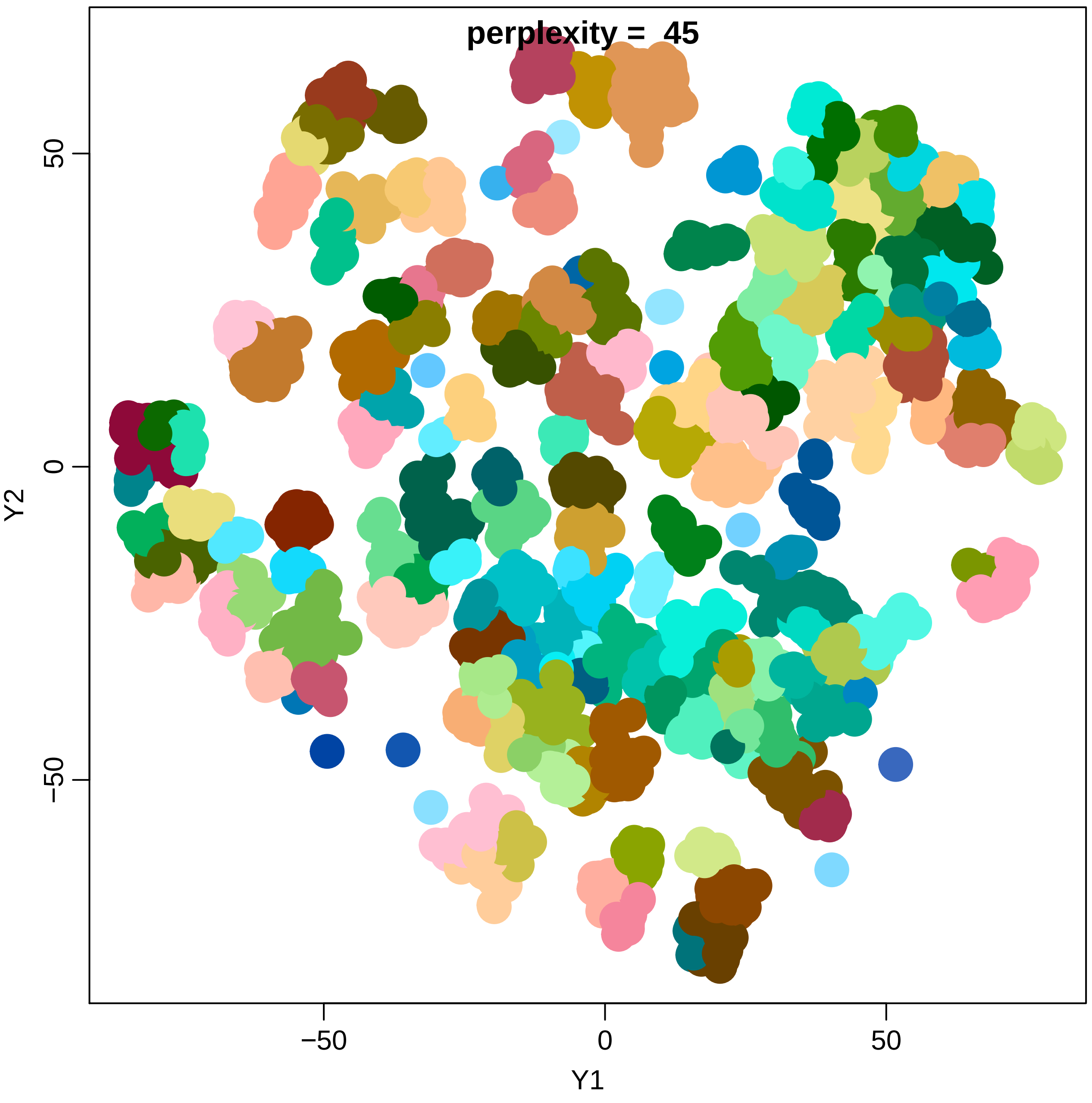

perplexity = 45
Y2
Y1
50
0
−50
−50
0
50

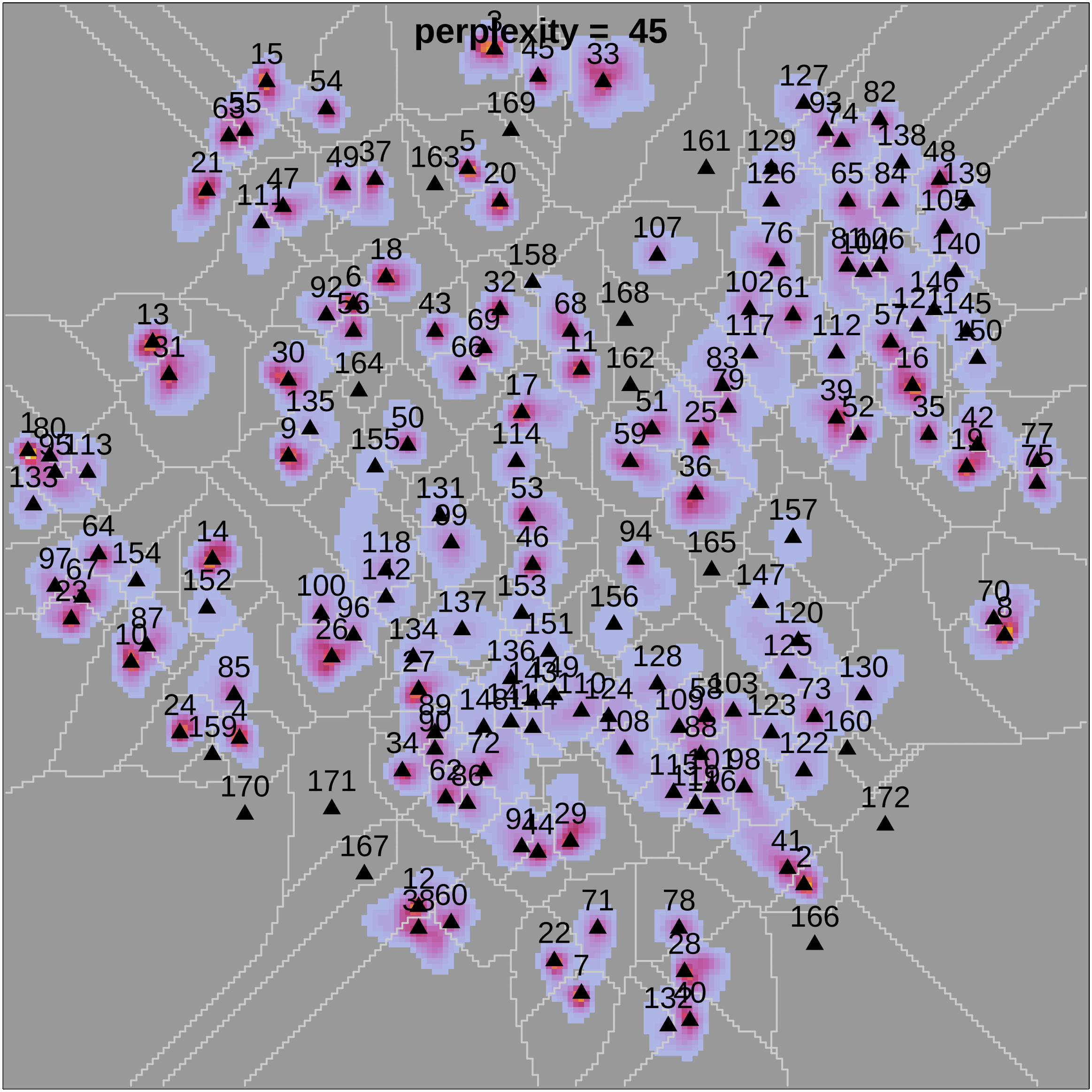
perplexity = 45

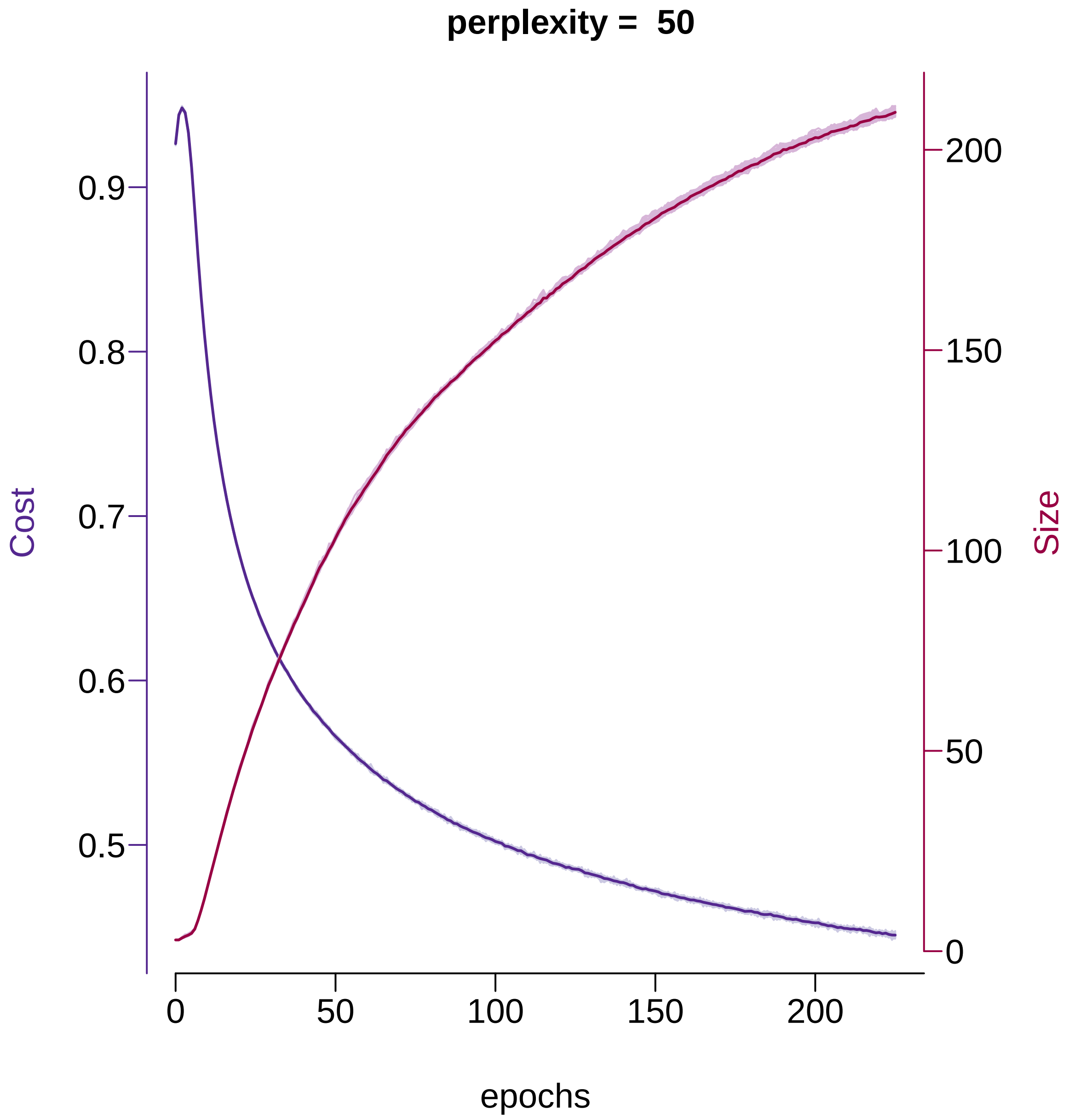
perplexity = 50
Cost
Size
epochs
0.9
0.8
0.7
0.6
0.5
200
150
100
50
0
0
50
100
150
200

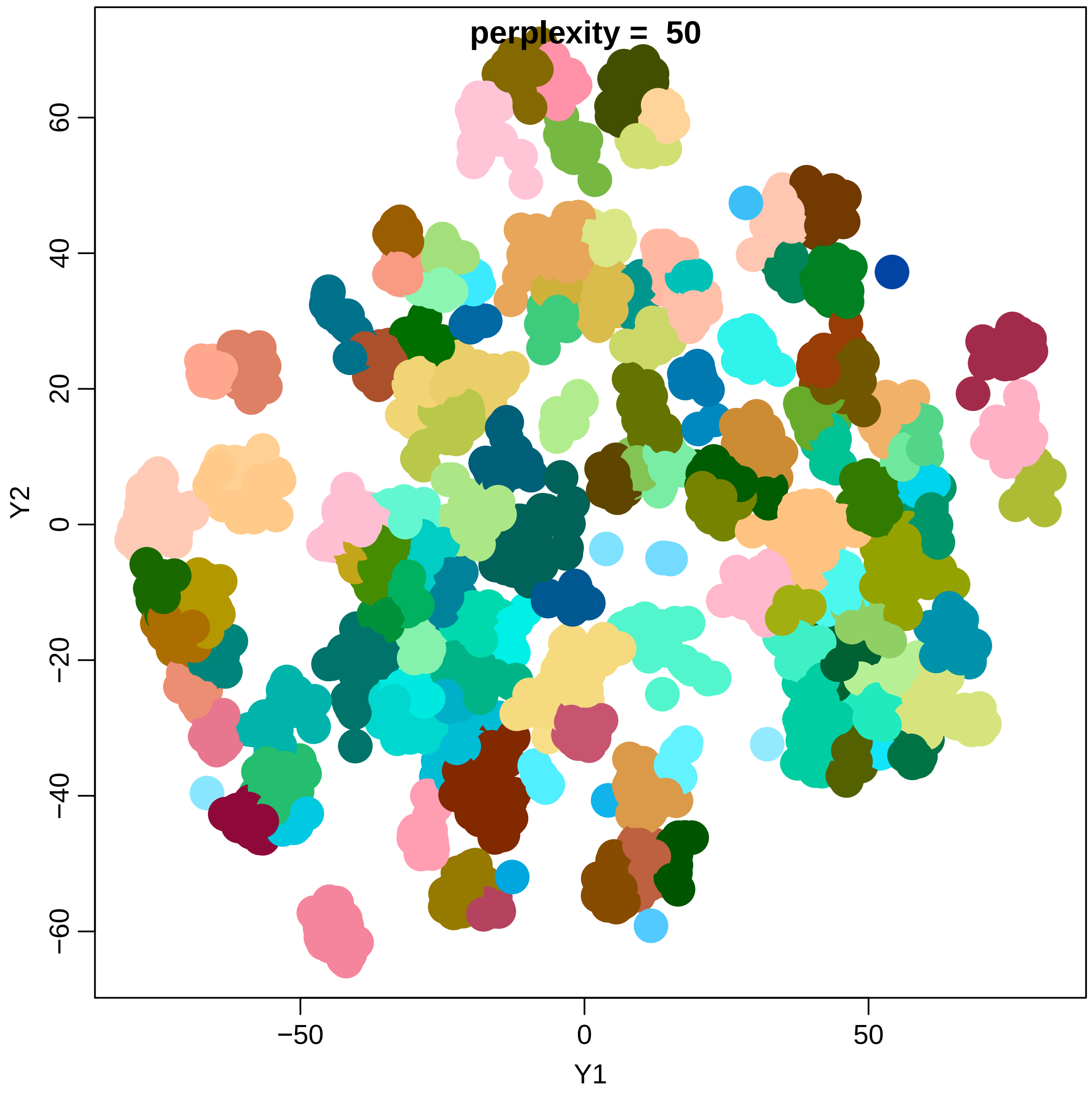
perplexity = 50
Y2
60
40
20
0
−20
−40
−60
Y1
−50
0
50

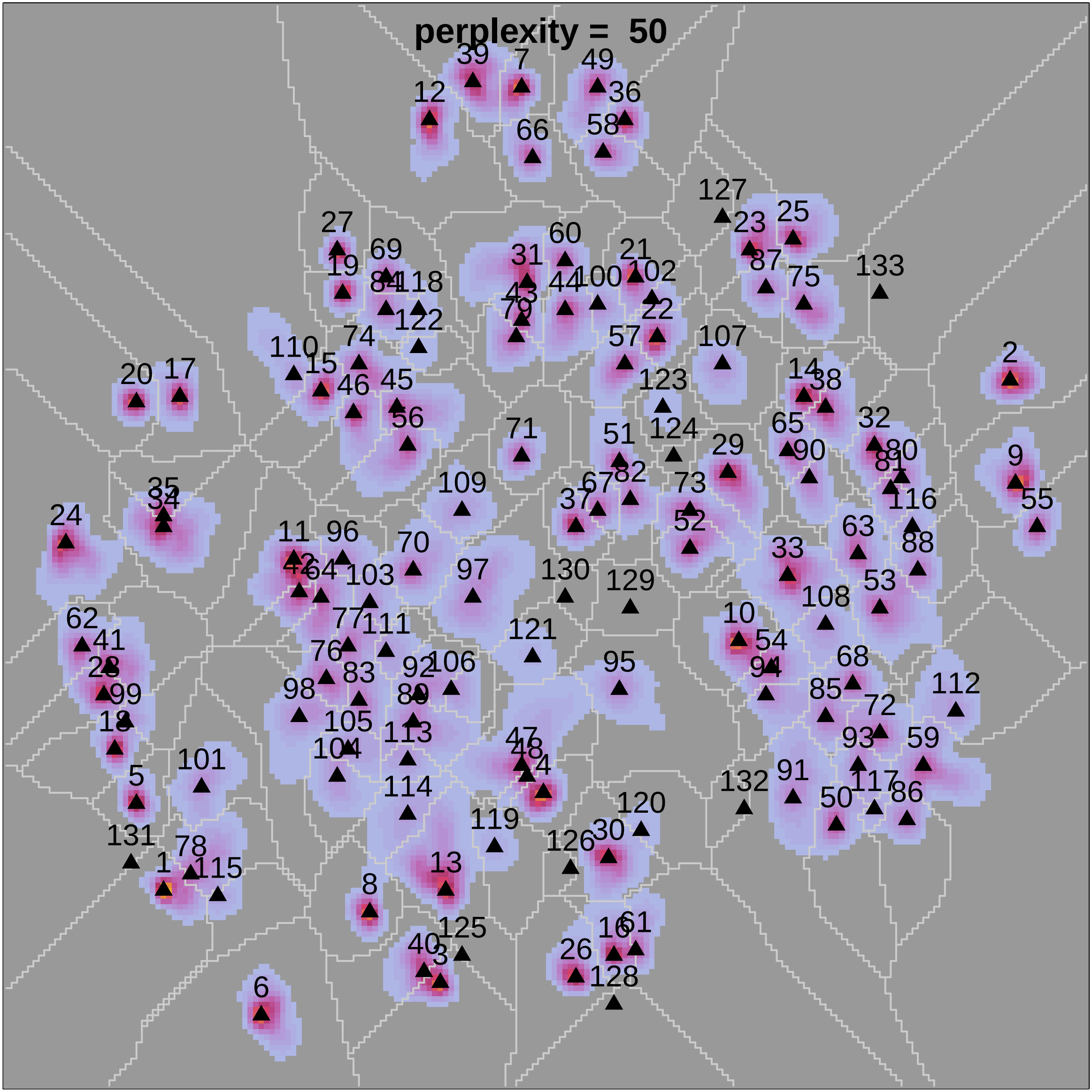
perplexity = 50

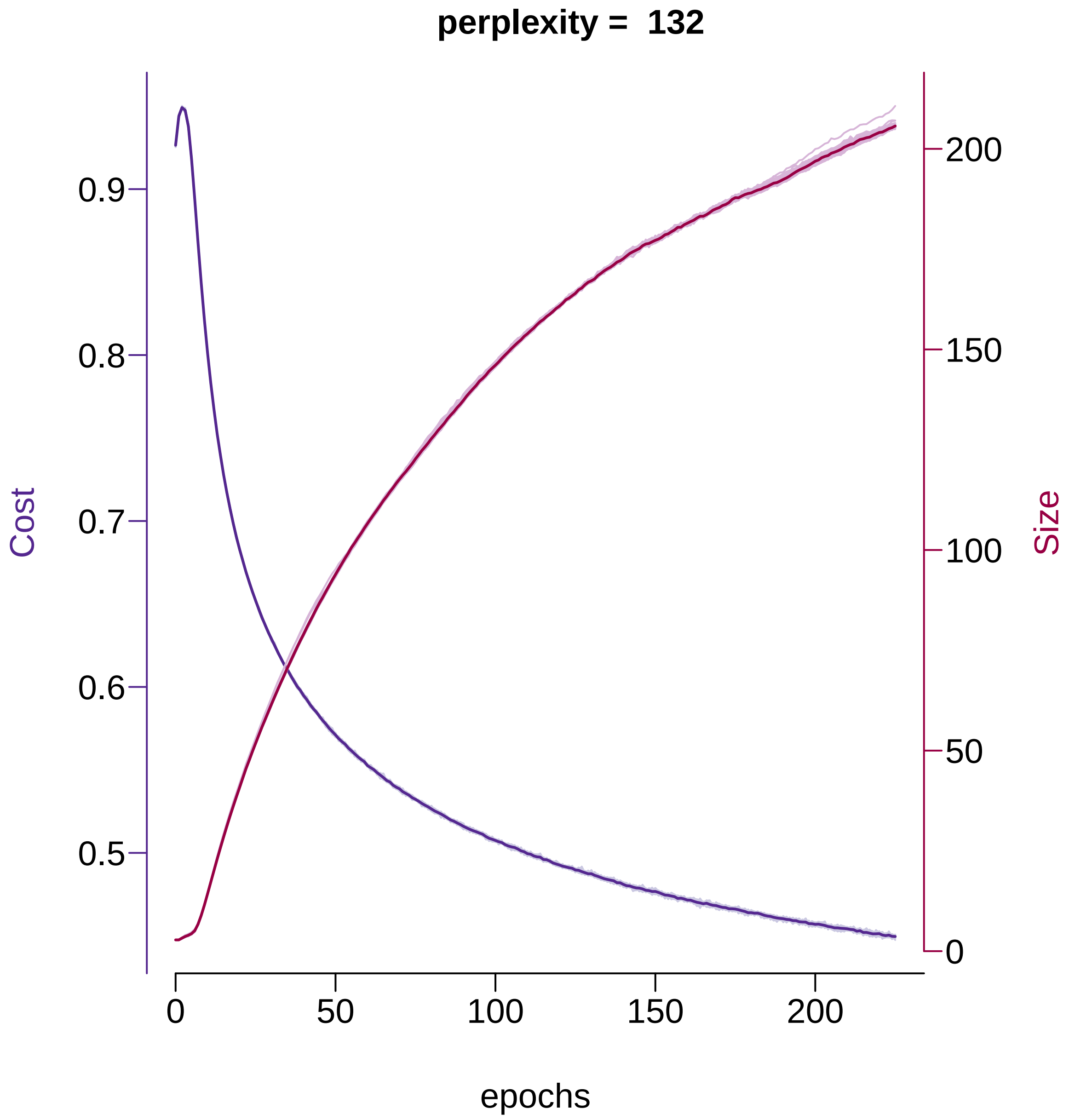
perplexity = 132
Cost
0.9
0.8
0.7
0.6
0.5
Size
200
150
100
50
0
0
50
100
150
200
epochs

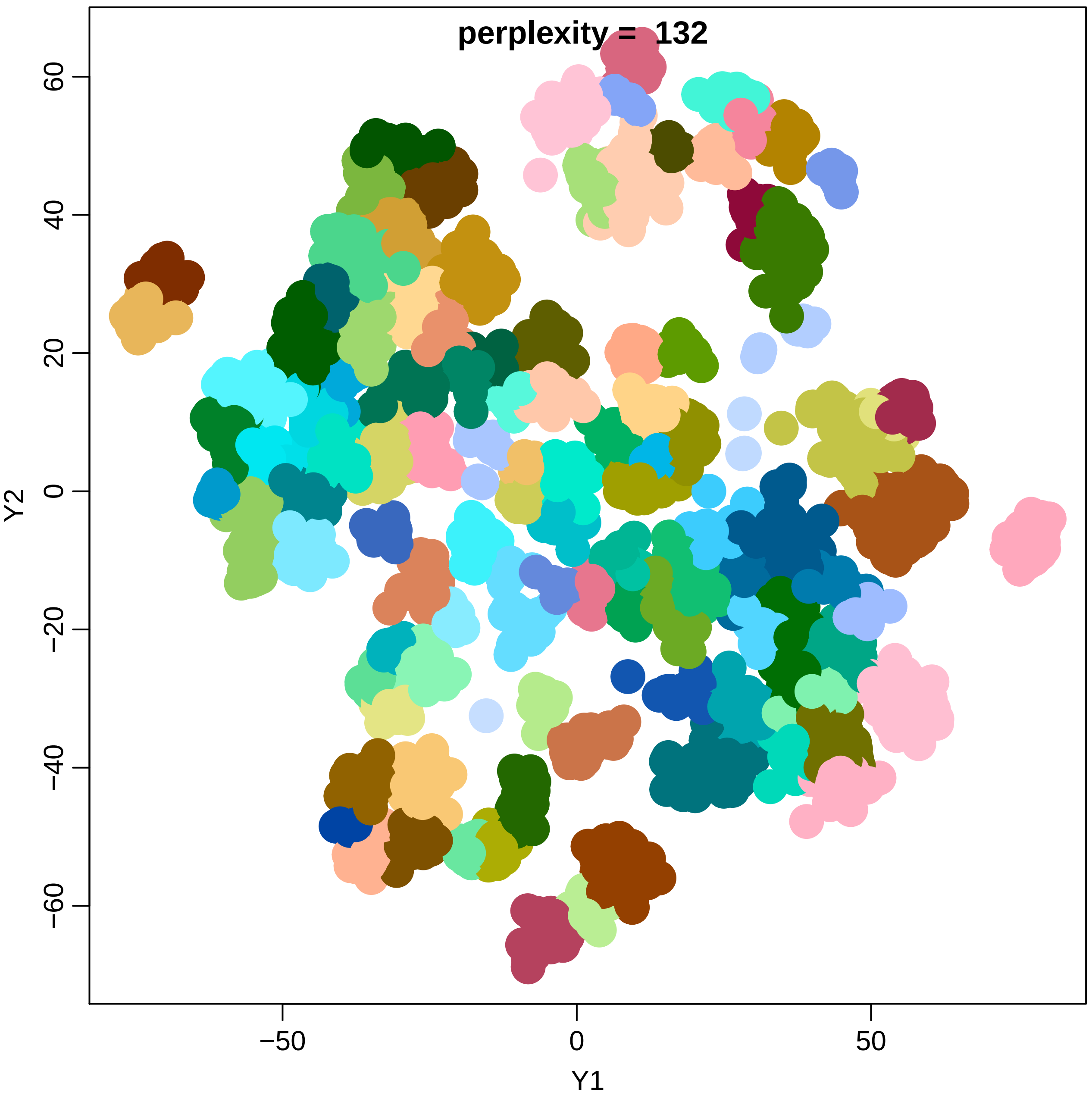
perplexity = 132
Y2
Y1
60
40
20
0
−20
−40
−60
−50
0
50

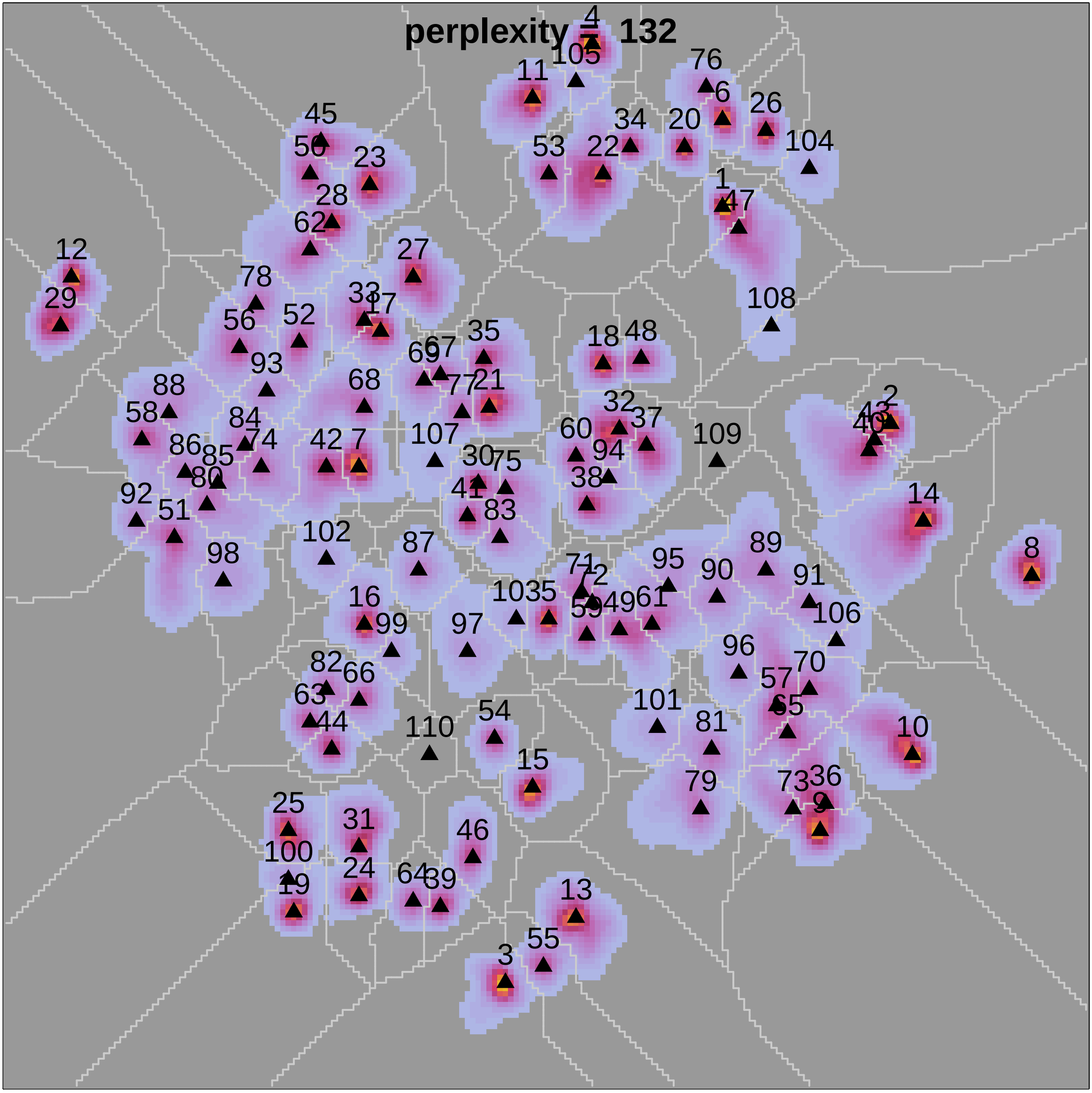

perplexity = 132
4
105
11
76
6
26
34
20
45
50
23
53
22
104
1
47
28
62
12
27
78
108
29
33
17
56
52
35
18
48
67
69
93
88
68
77
21
2
58
32
37
43
40
84
74
42
7
107
60
109
86
85
30
75
94
80
38
41
92
83
14
51
102
8
87
98
95
71
72
90
89
91
16
103
5
59
49
61
99
97
106
96
82
70
66
57
63
65
101
44
54
81
110
10
15
79
36
73
25
9
31
46
100
24
64
39
19
13
55
3

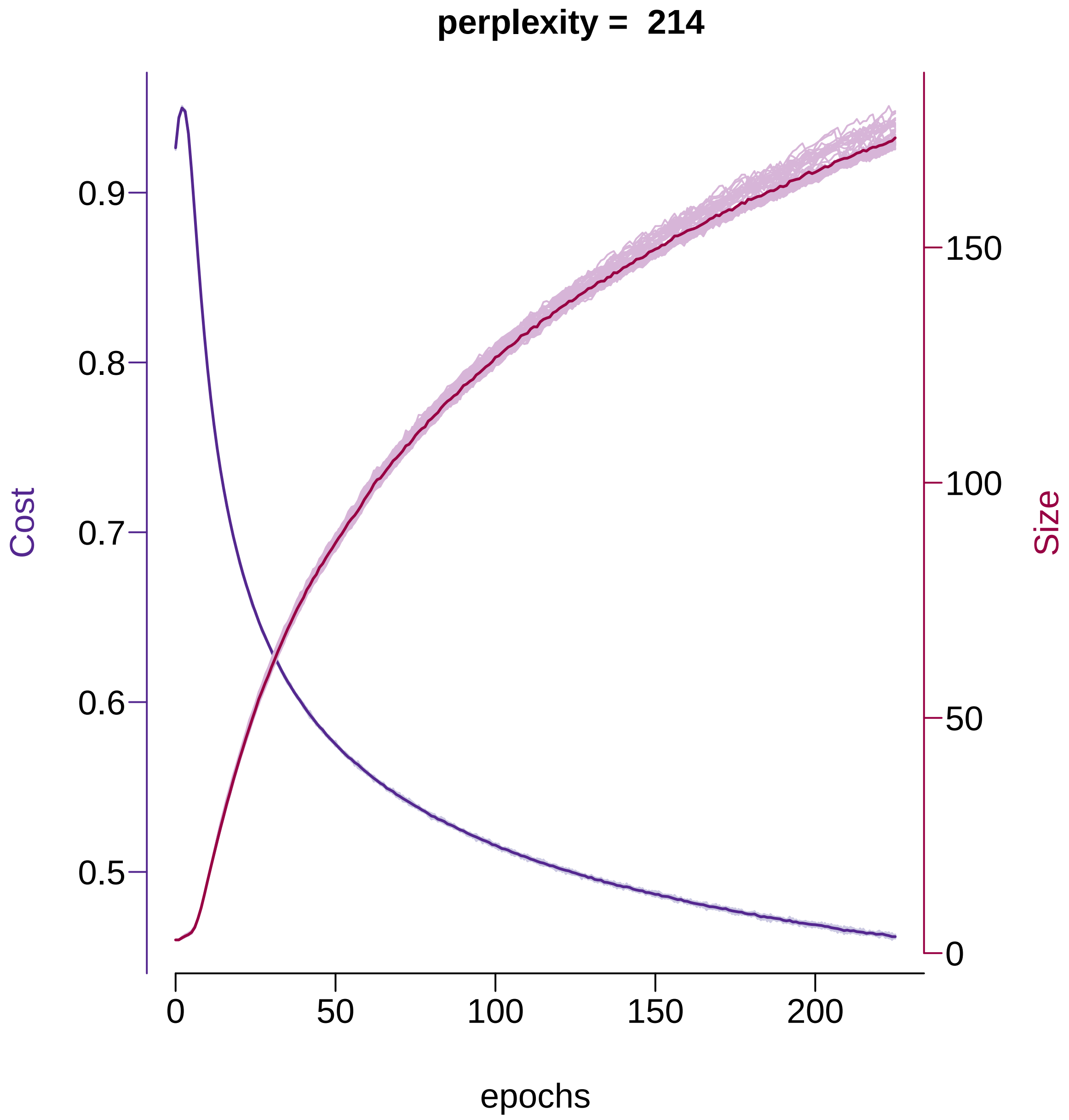

perplexity = 214
Cost
0.9
0.8
0.7
0.6
0.5
Size
150
100
50
0
0
50
100
150
200
epochs

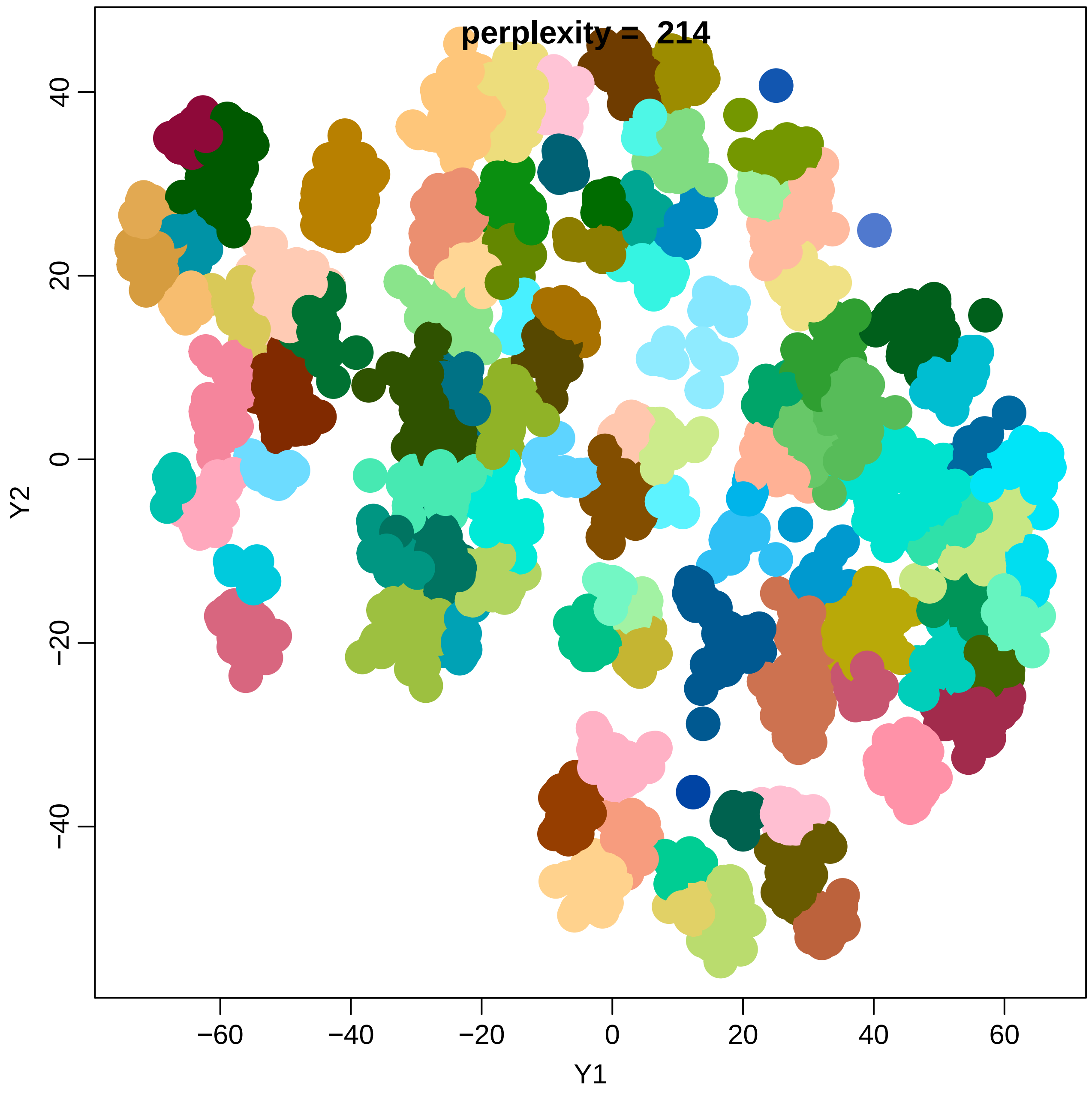

perplexity = 214
Y2
40
20
0
−20
−40
Y1
−60
−40
−20
0
20
40
60

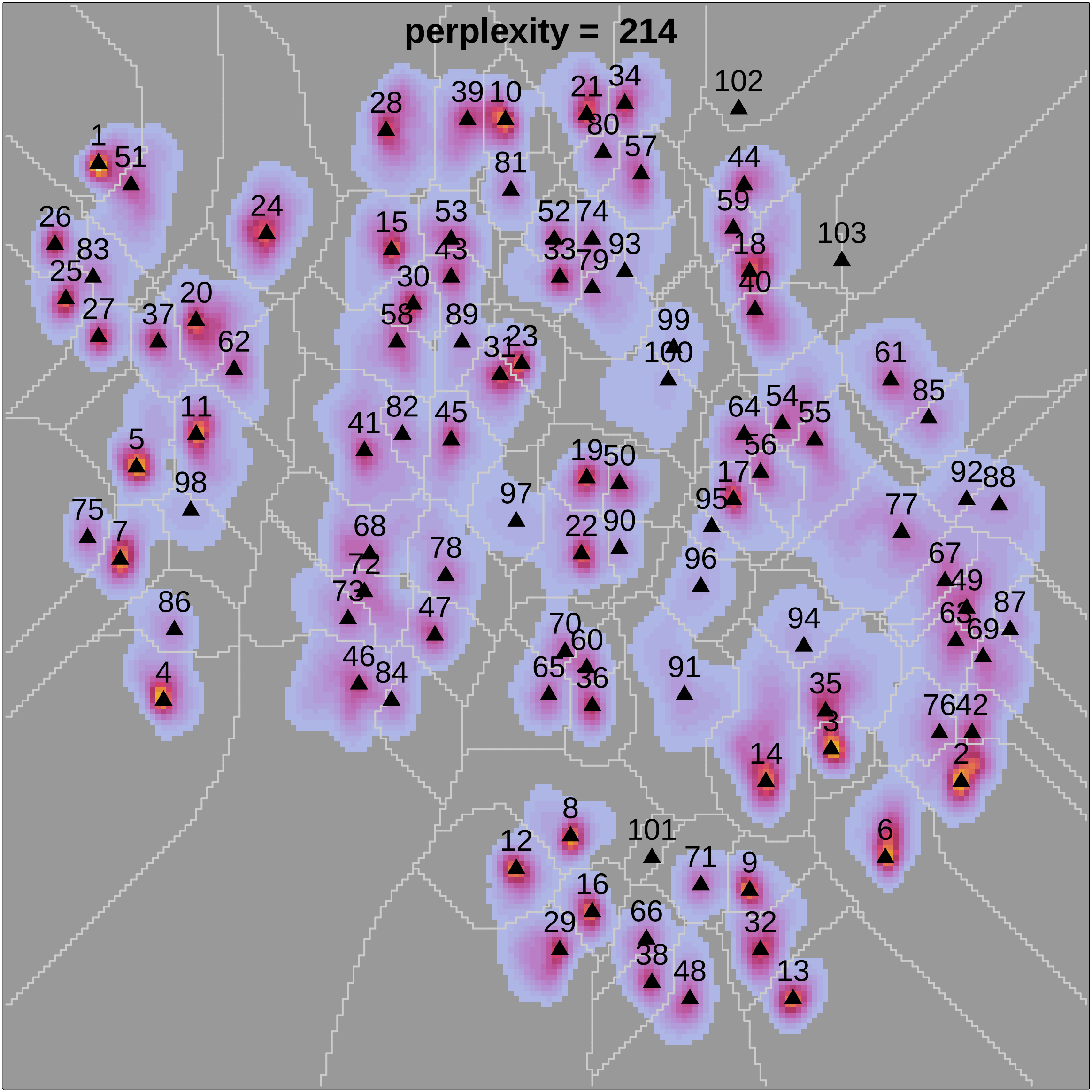
perplexity = 214

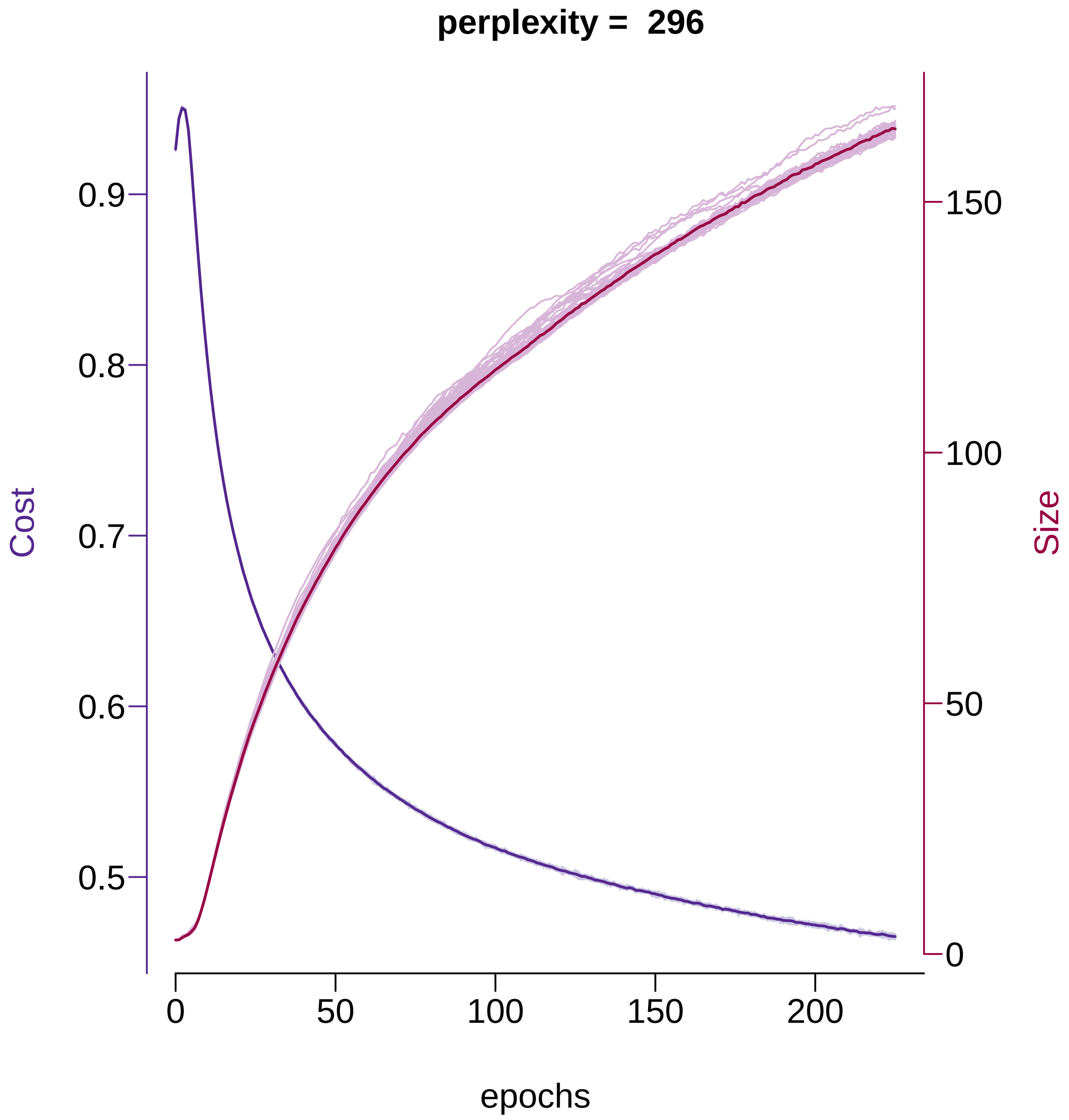
perplexity = 296
Cost
0.9
0.8
0.7
0.6
0.5
Size
150
100
50
0
0
50
100
150
200
epochs

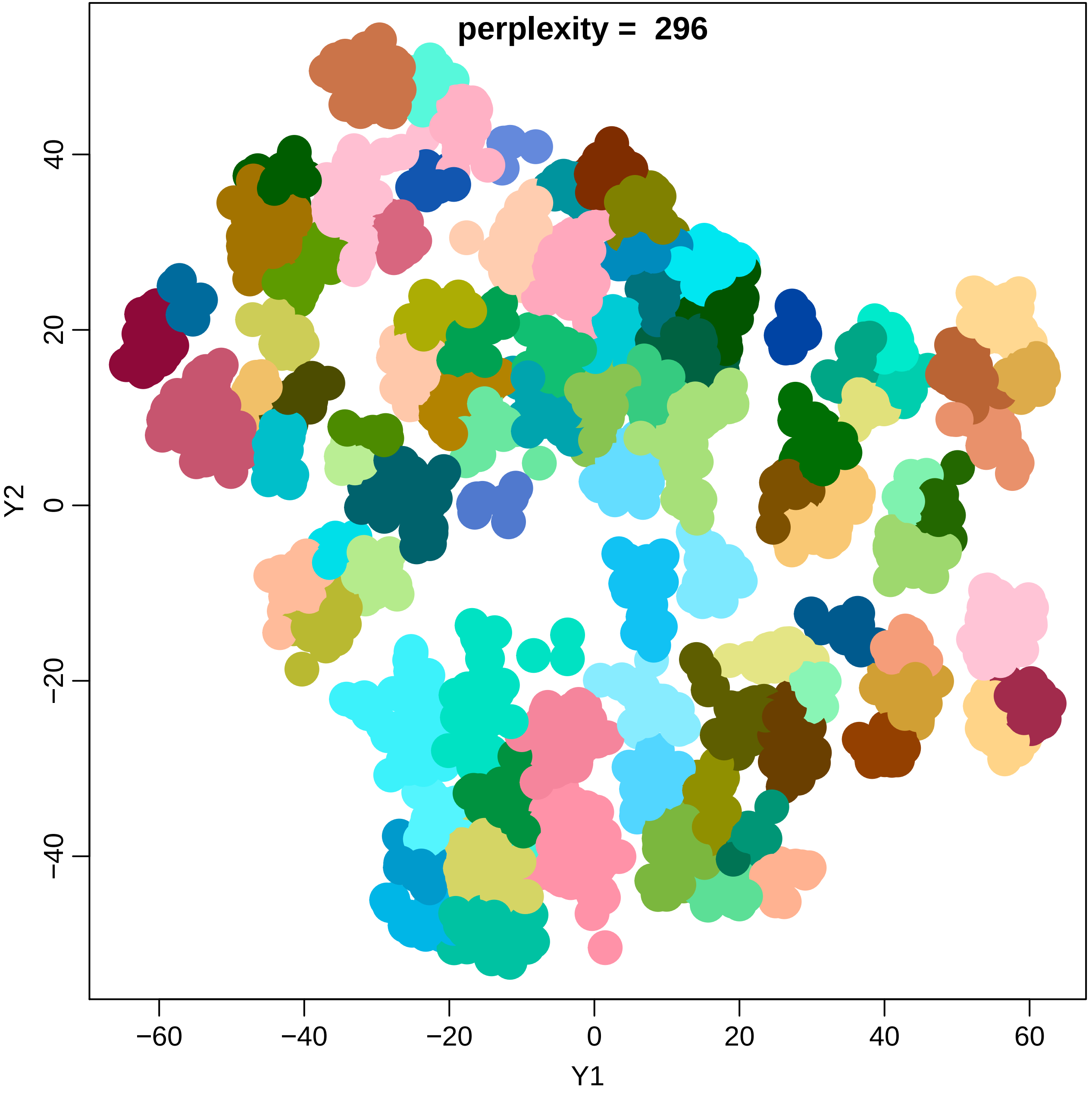
perplexity = 296
Y2
40
20
0
−20
−40
Y1
−60
−40
−20
0
20
40
60

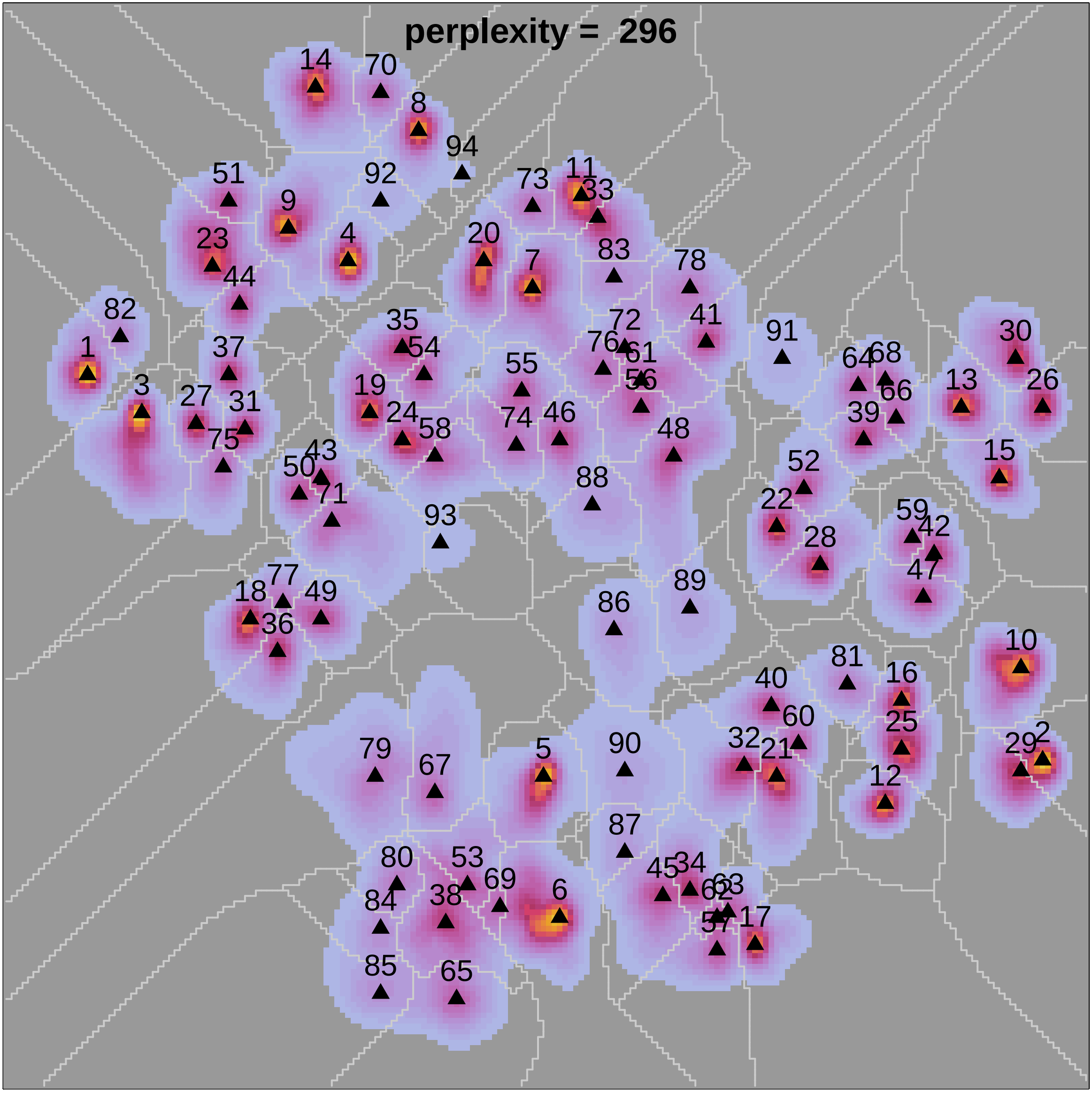
perplexity = 296
14
70
8
94
51
92
9
73
11
33
23
4
20
7
83
78
44
82
35
72
41
91
1
37
54
76
61
64
68
30
55
13
26
3
19
66
27
31
24
74
46
39
75
58
48
43
15
50
52
71
88
22
93
59
42
28
47
77
89
18
49
86
36
10
81
40
16
60
25
79
67
5
90
32
21
29
2
12
87
80
53
45
34
69
6
63
84
38
17
85
65

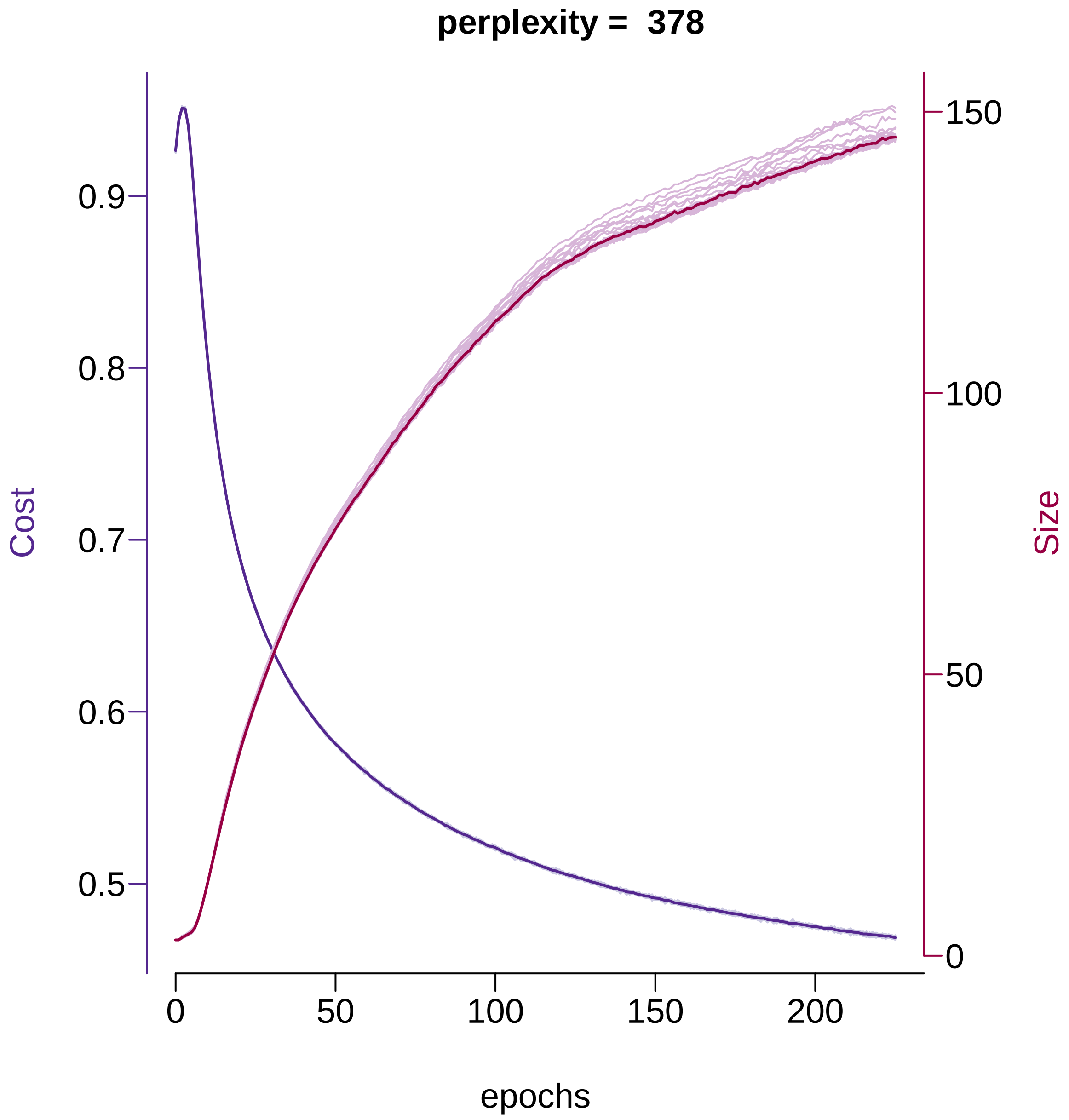
perplexity = 378
0.9
0.8
0.7
0.6
0.5
Cost
150
100
50
0
Size
0
50
100
150
200
epochs

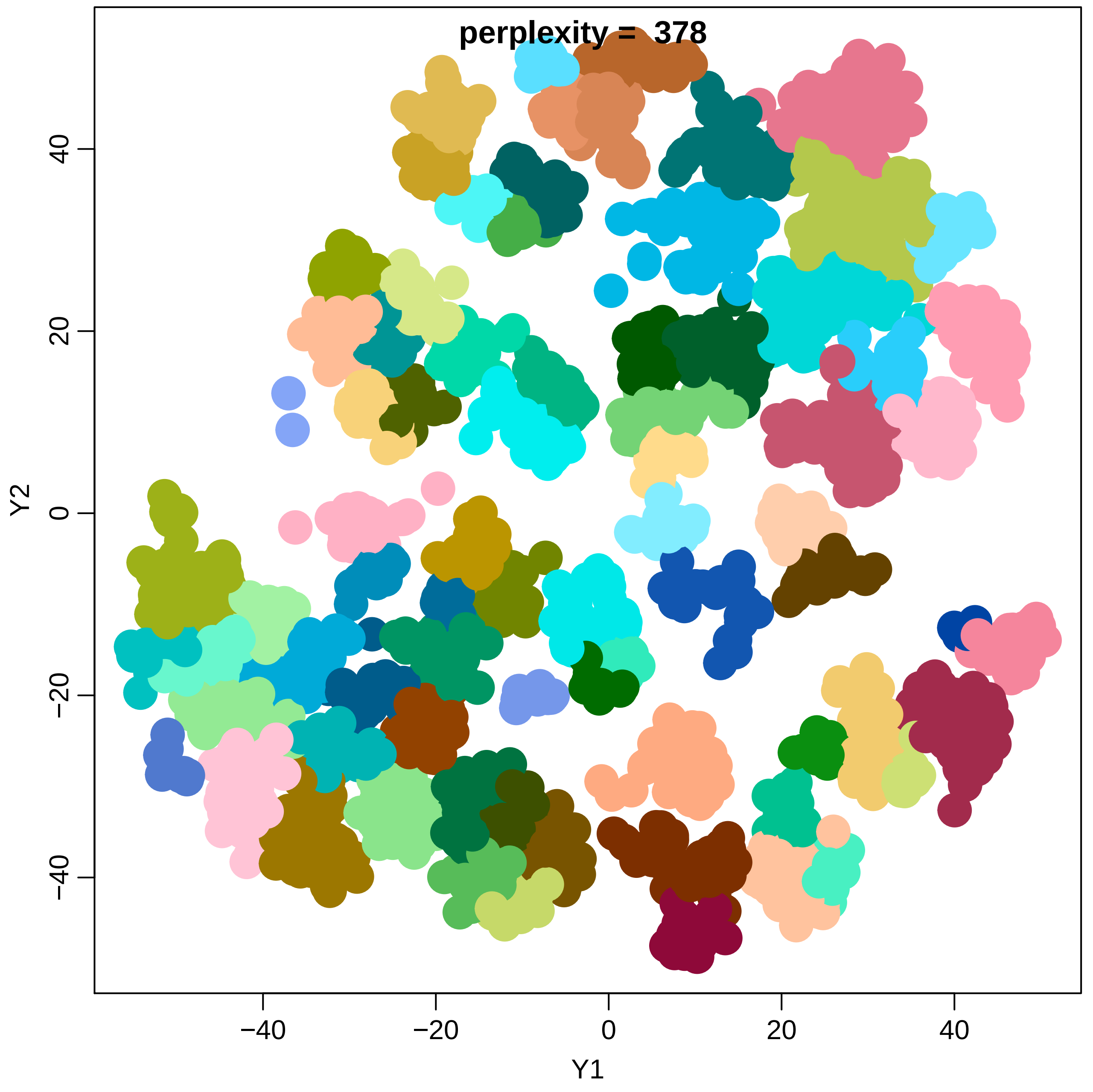
perplexity = 378
Y1
Y2
−40
−20
0
20
40
−40
−20
0
20
40

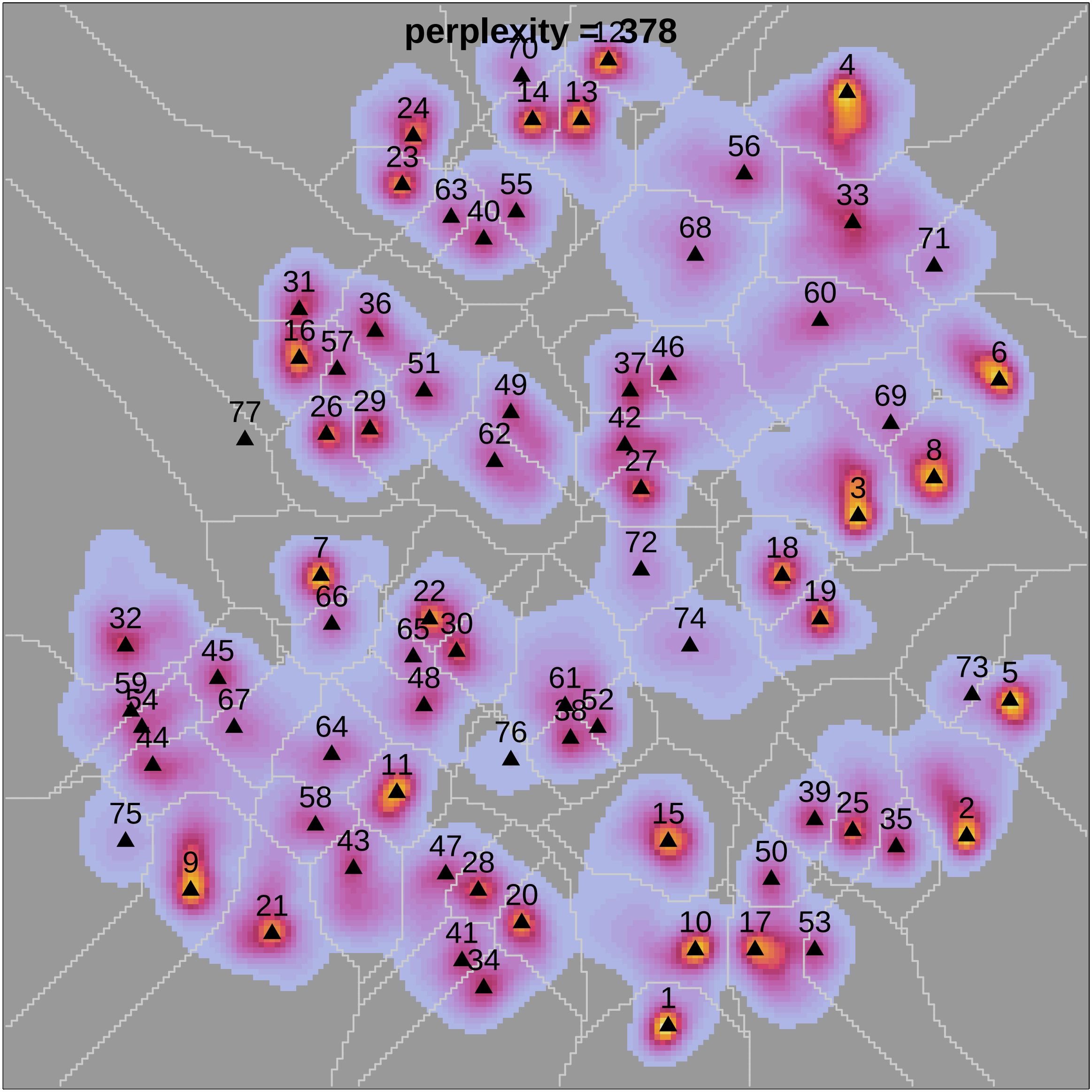
perplexity = 378

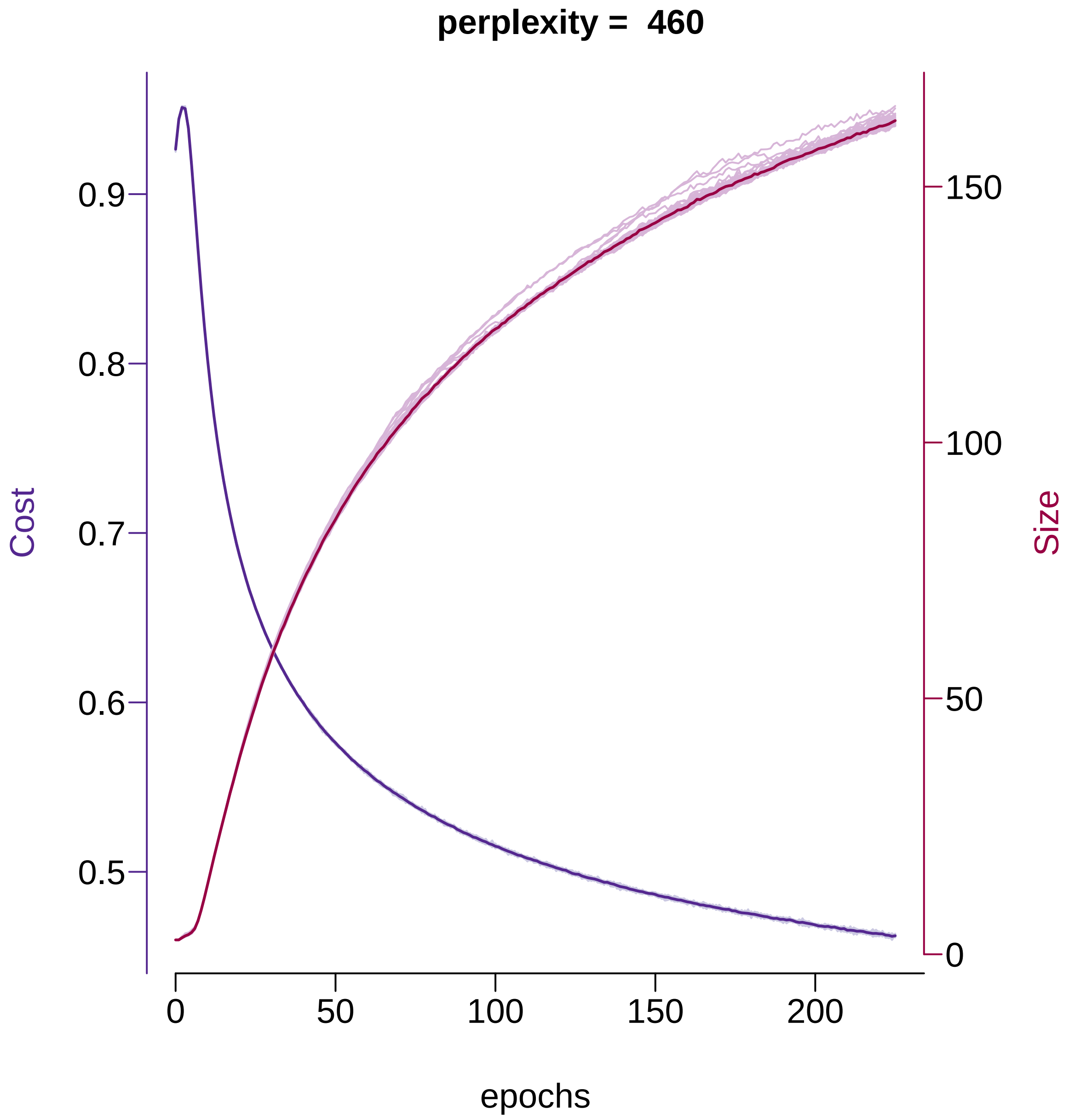
perplexity = 460
Cost
Size
epochs
0.9
0.8
0.7
0.6
0.5
150
100
50
0
0
50
100
150
200

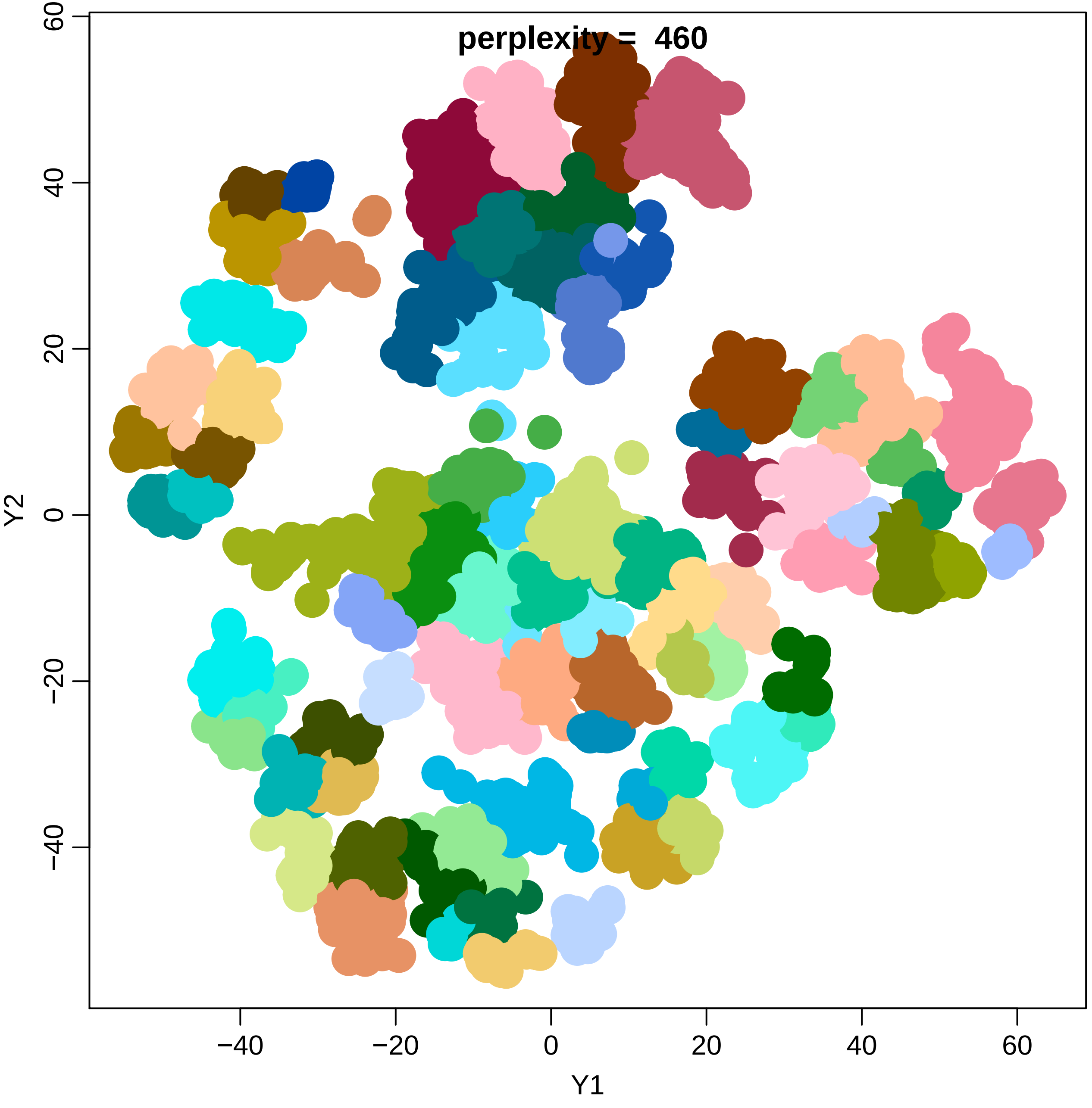
perplexity = 460
Y2
60
40
20
0
−20
−40
Y1
−40
−20
0
20
40
60

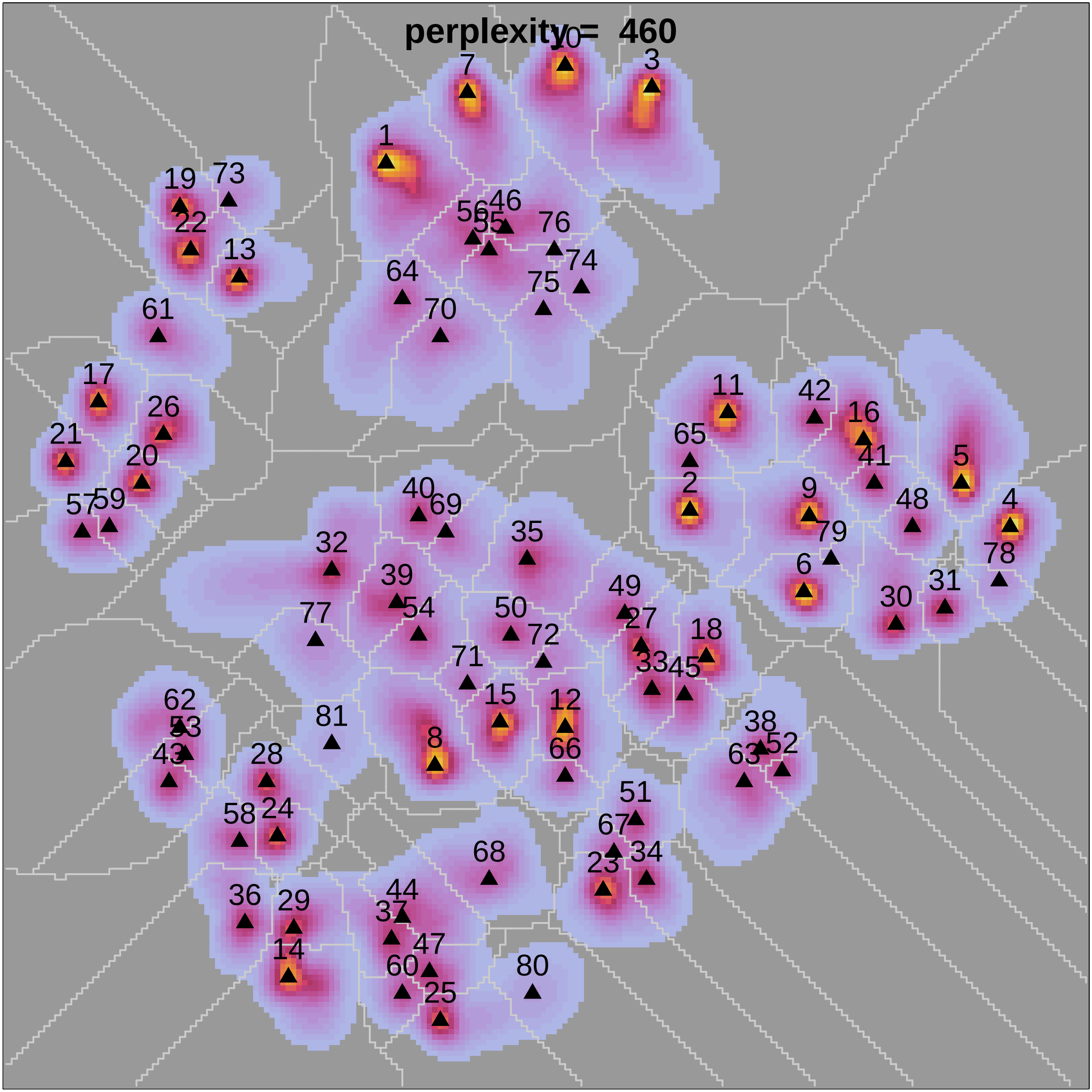
perplexity = 460
10
7
3
1
19
73
22
13
56
46
55
76
74
64
75
70
61
17
26
21
20
57
59
11
42
16
65
41
5
2
9
48
4
79
78
6
31
30
40
69
35
32
39
54
50
49
27
72
18
77
71
33
45
15
12
62
53
43
81
8
66
38
52
63
28
51
58
24
67
34
23
68
36
29
44
37
47
14
60
80
25

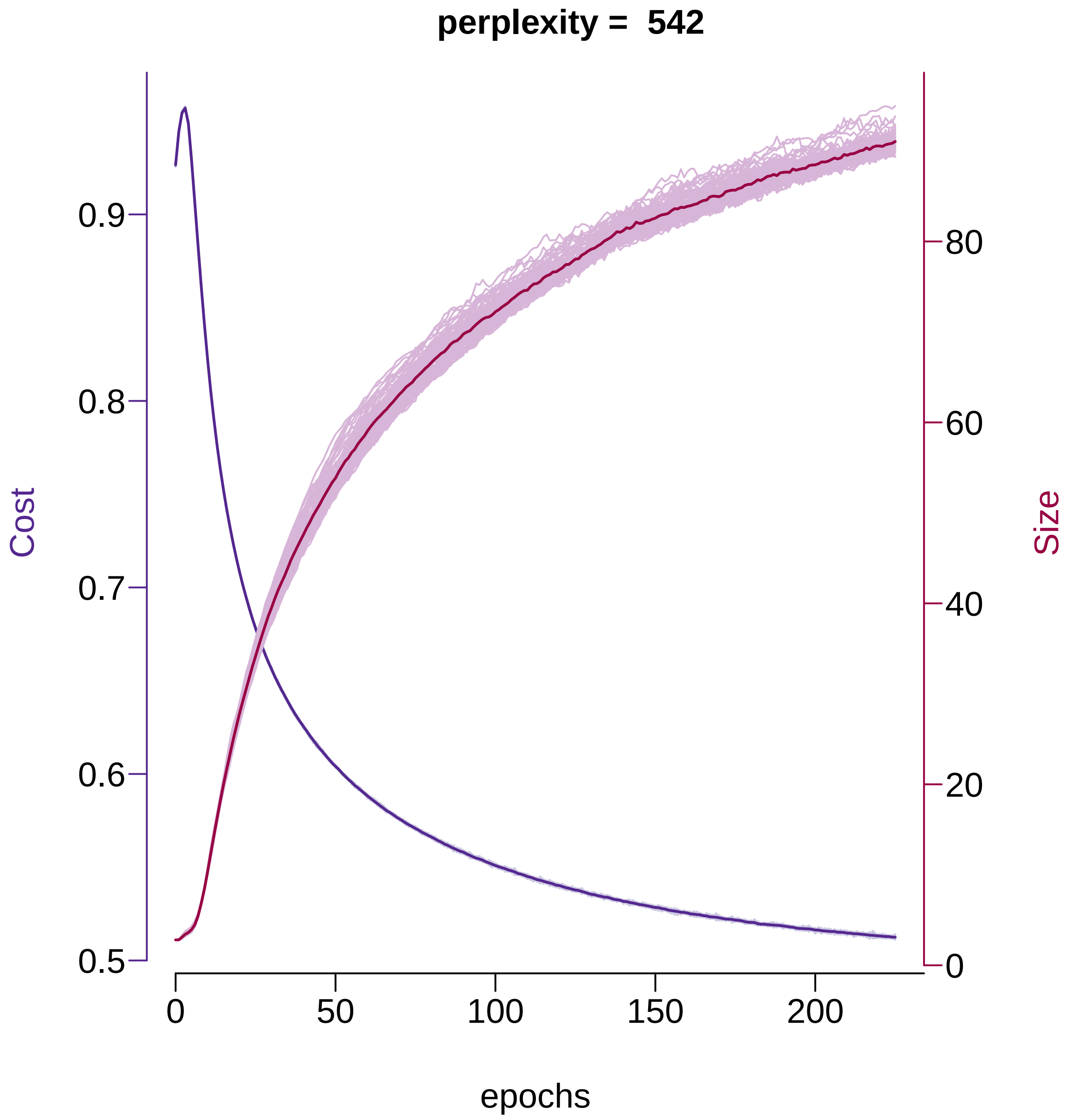
perplexity = 542
Cost
Size
epochs
0.9
0.8
0.7
0.6
0.5
80
60
40
20
0
0
50
100
150
200

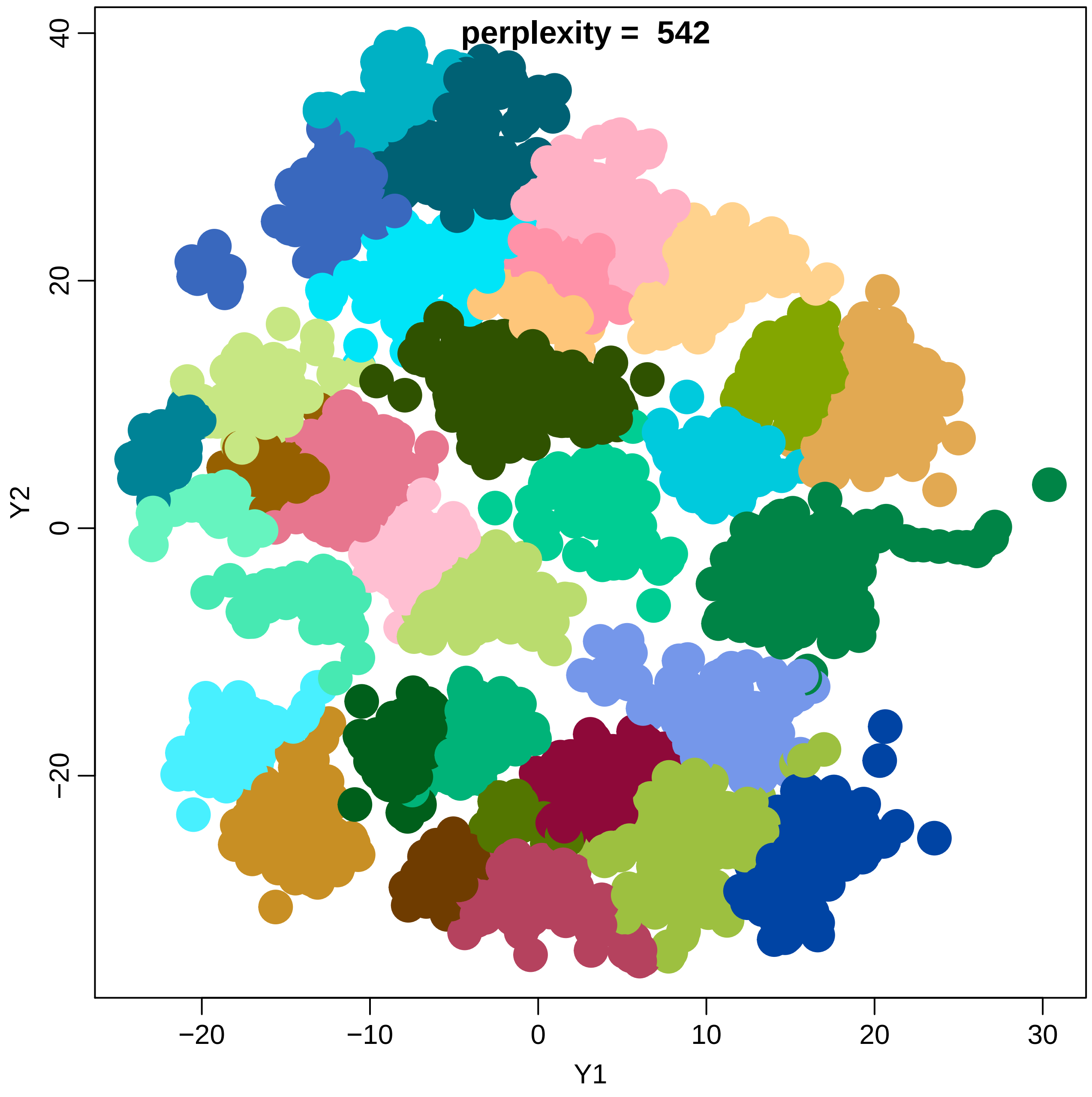
perplexity = 542
Y2
Y1
40
20
0
−20
−20
−10
0
10
20
30

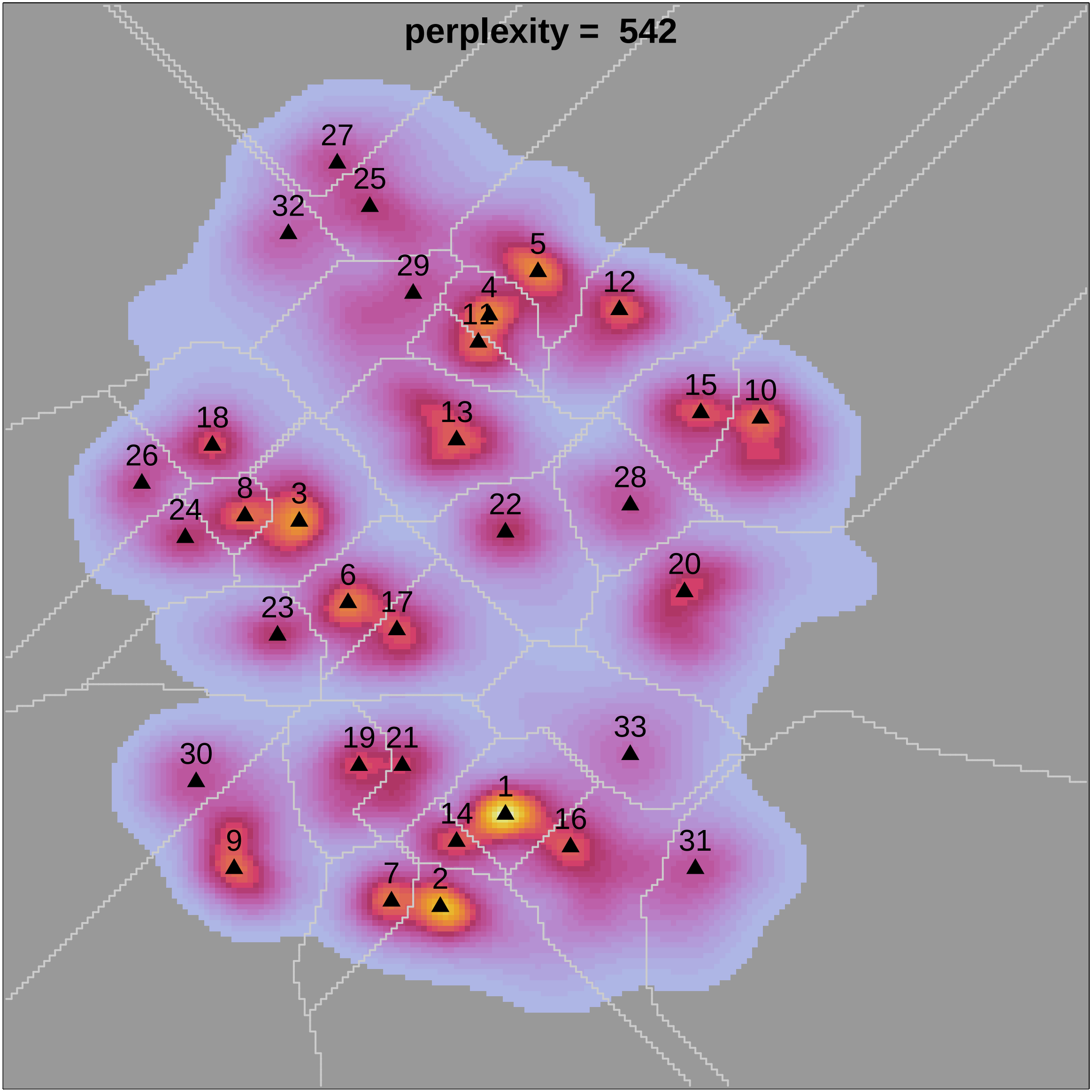
perplexity = 542
27
25
32
29
5
4
11
12
15
10
18
13
26
8
3
24
22
28
6
23
17
20
30
19
21
33
1
14
16
9
31
7
2

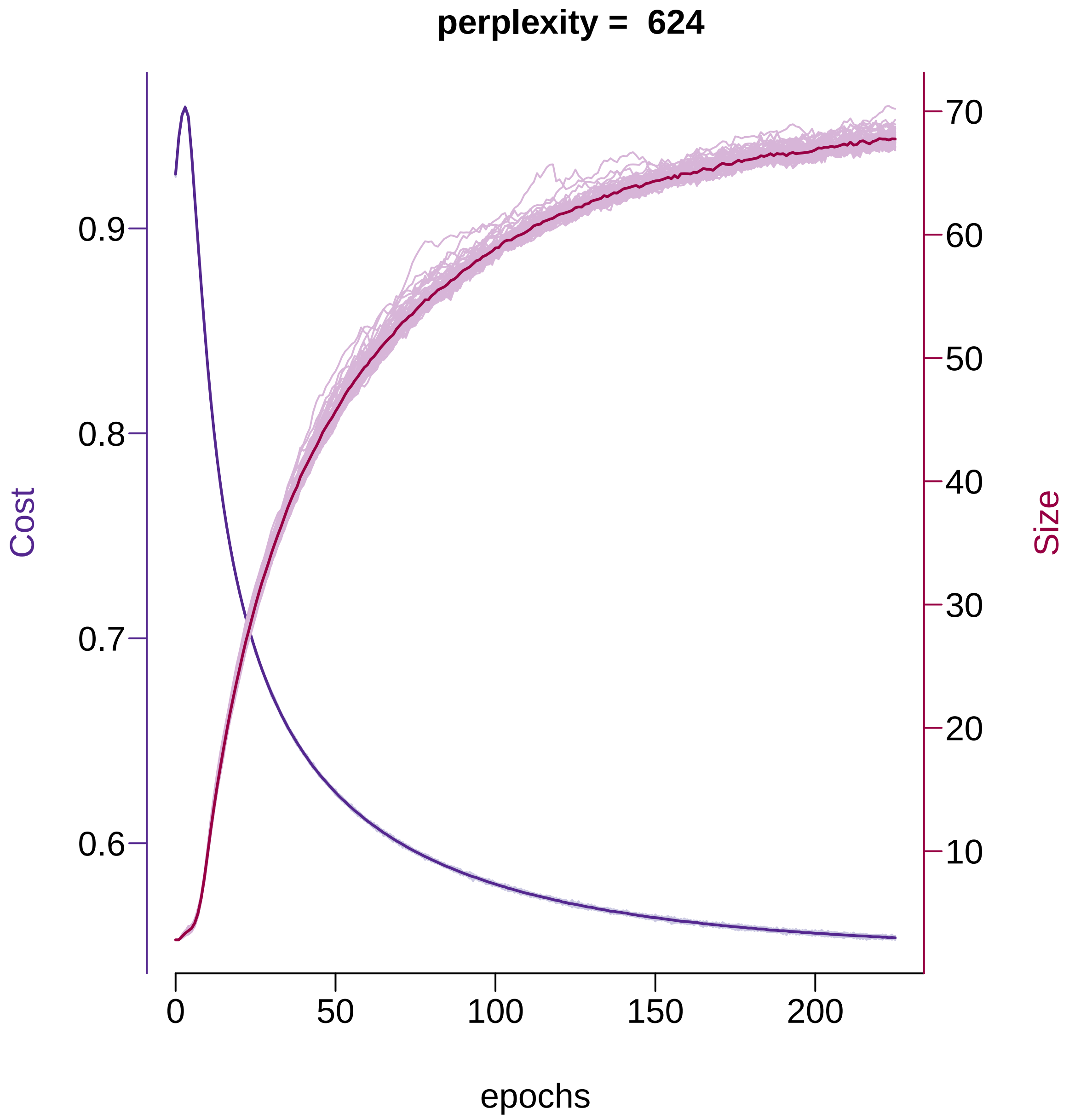
perplexity = 624
Cost
0.9
0.8
0.7
0.6
Size
70
60
50
40
30
20
10
0
50
100
150
200
epochs

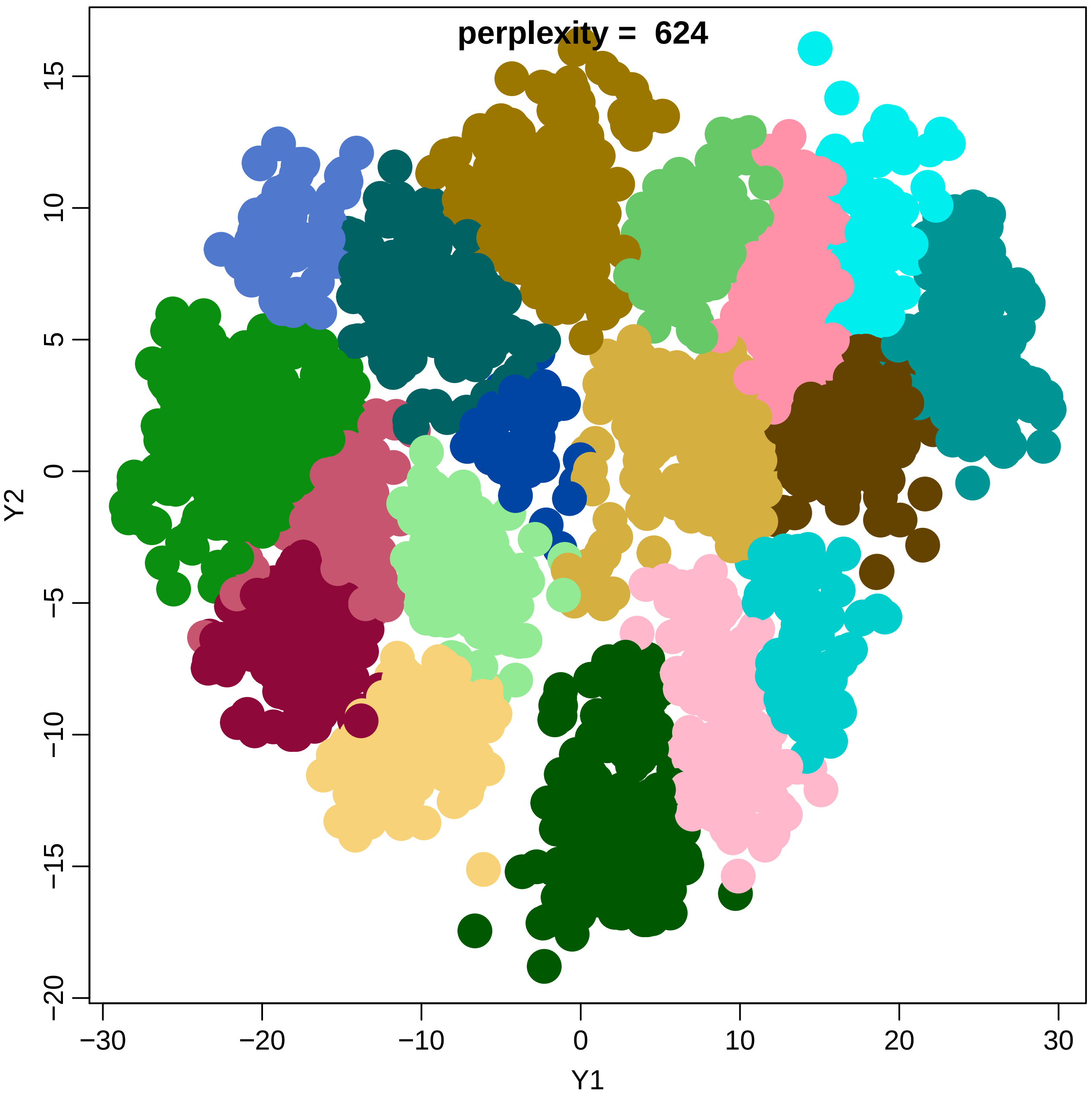

perplexity = 624
Y2
Y1
15
10
5
0
−5
−10
−15
−20
−30
−20
−10
0
10
20
30

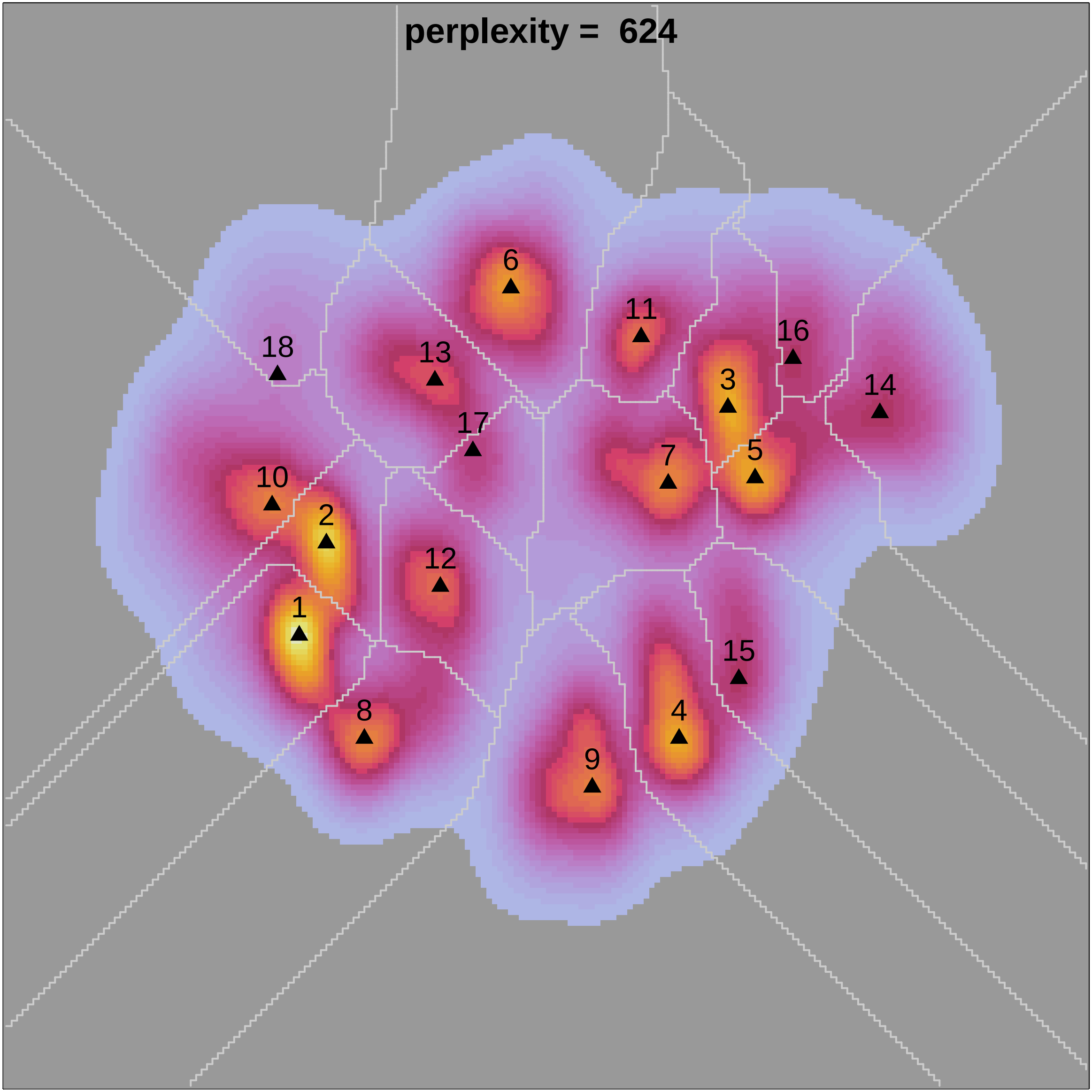
perplexity = 624
6
11
16
18
13
3
14
17
7
5
10
2
12
1
15
8
4
9

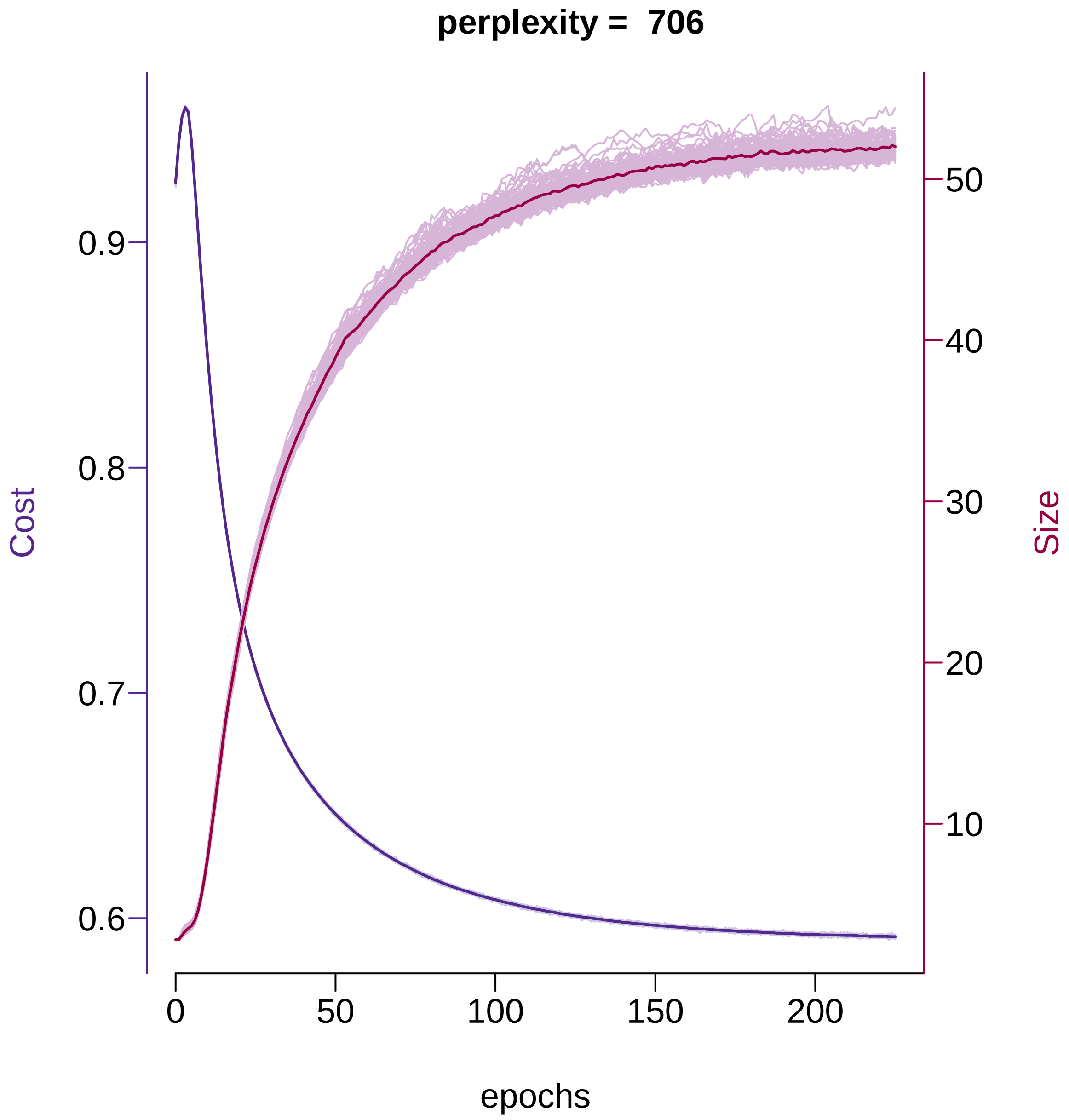

perplexity = 706
Cost
0.9
0.8
0.7
0.6
Size
50
40
30
20
10
0
50
100
150
200
epochs

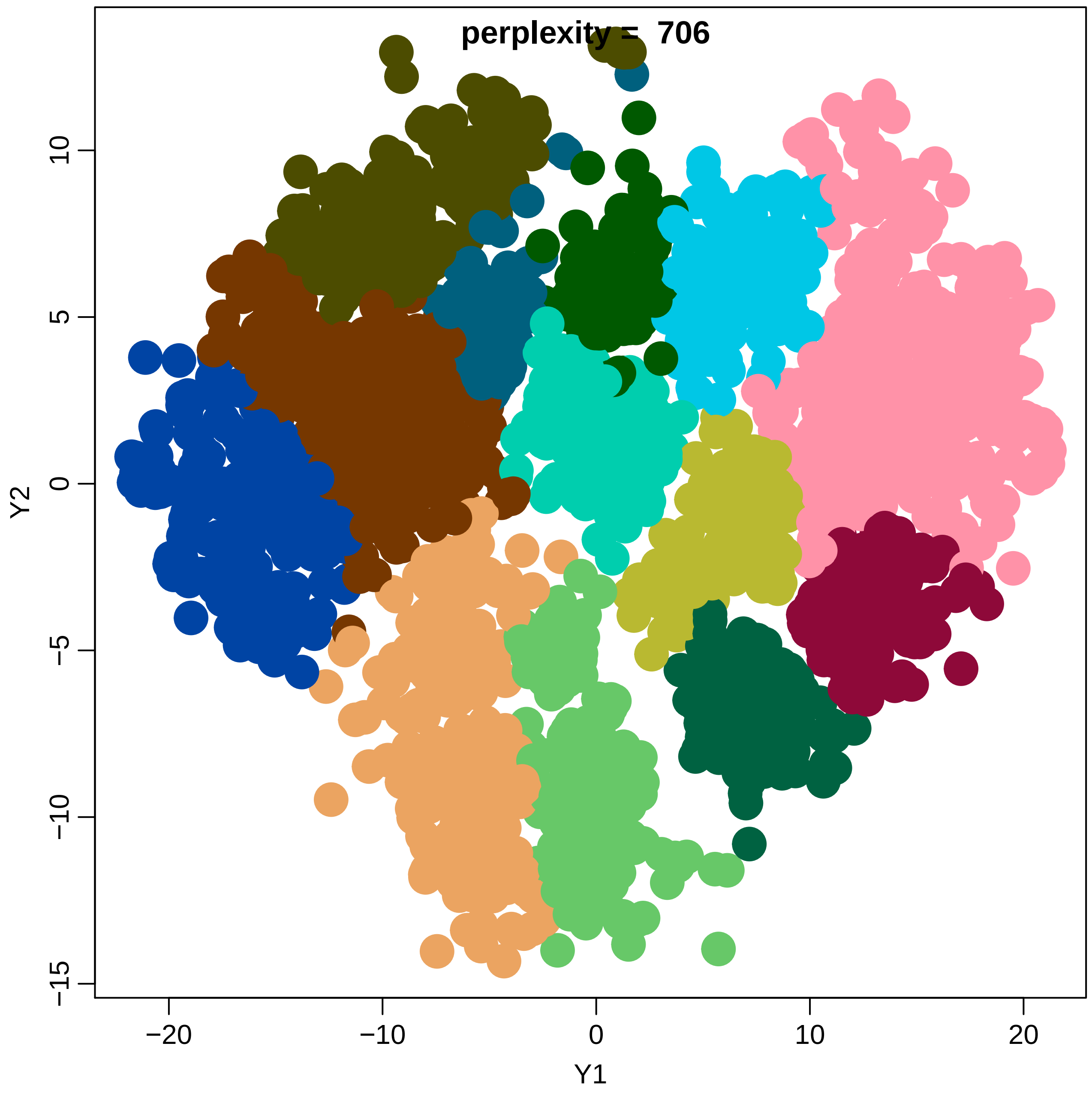
perplexity = 706
Y2
10
5
0
−5
−10
−15
Y1
−20
−10
0
10
20

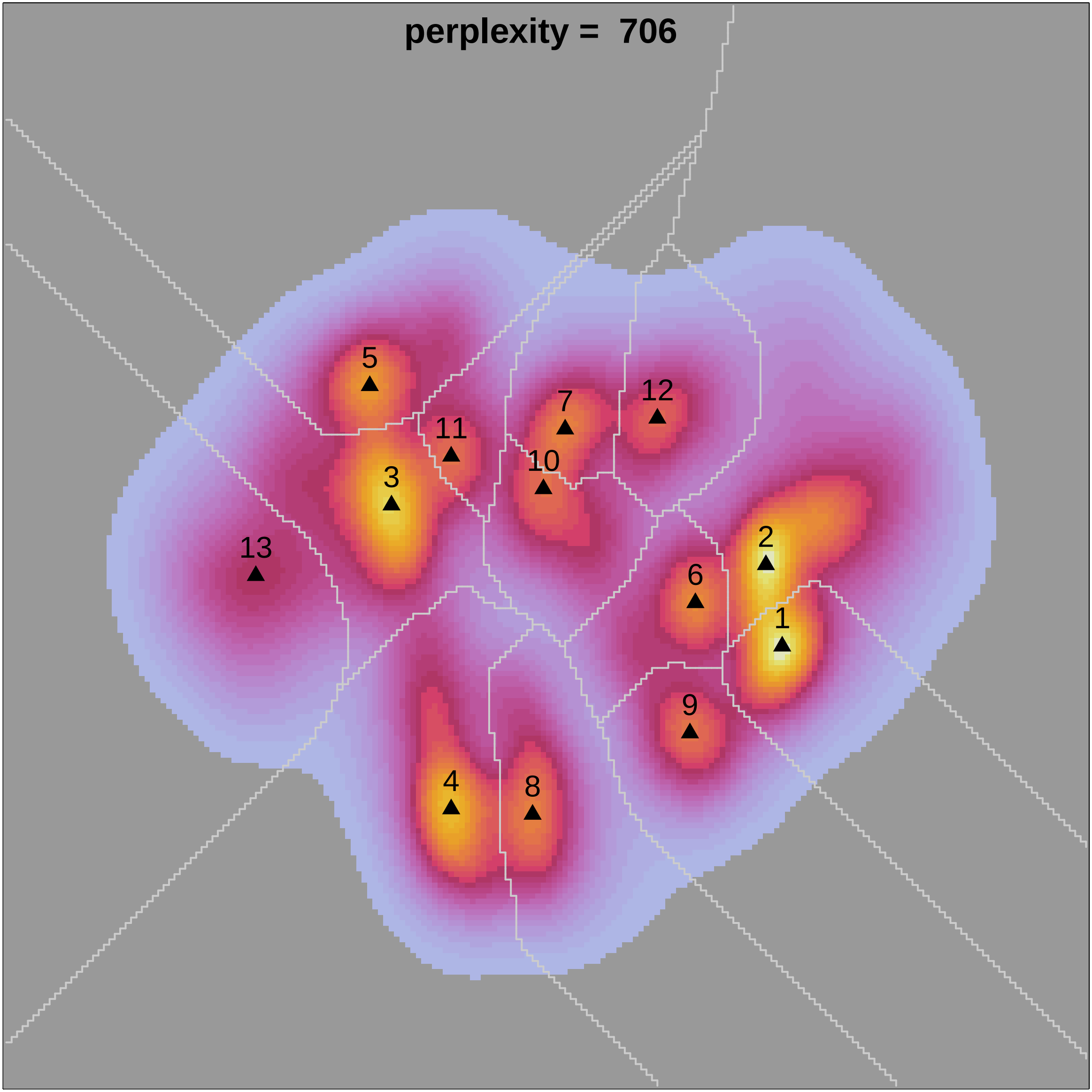
perplexity = 706
1
2
3
4
5
6
7
8
9
10
11
12
13

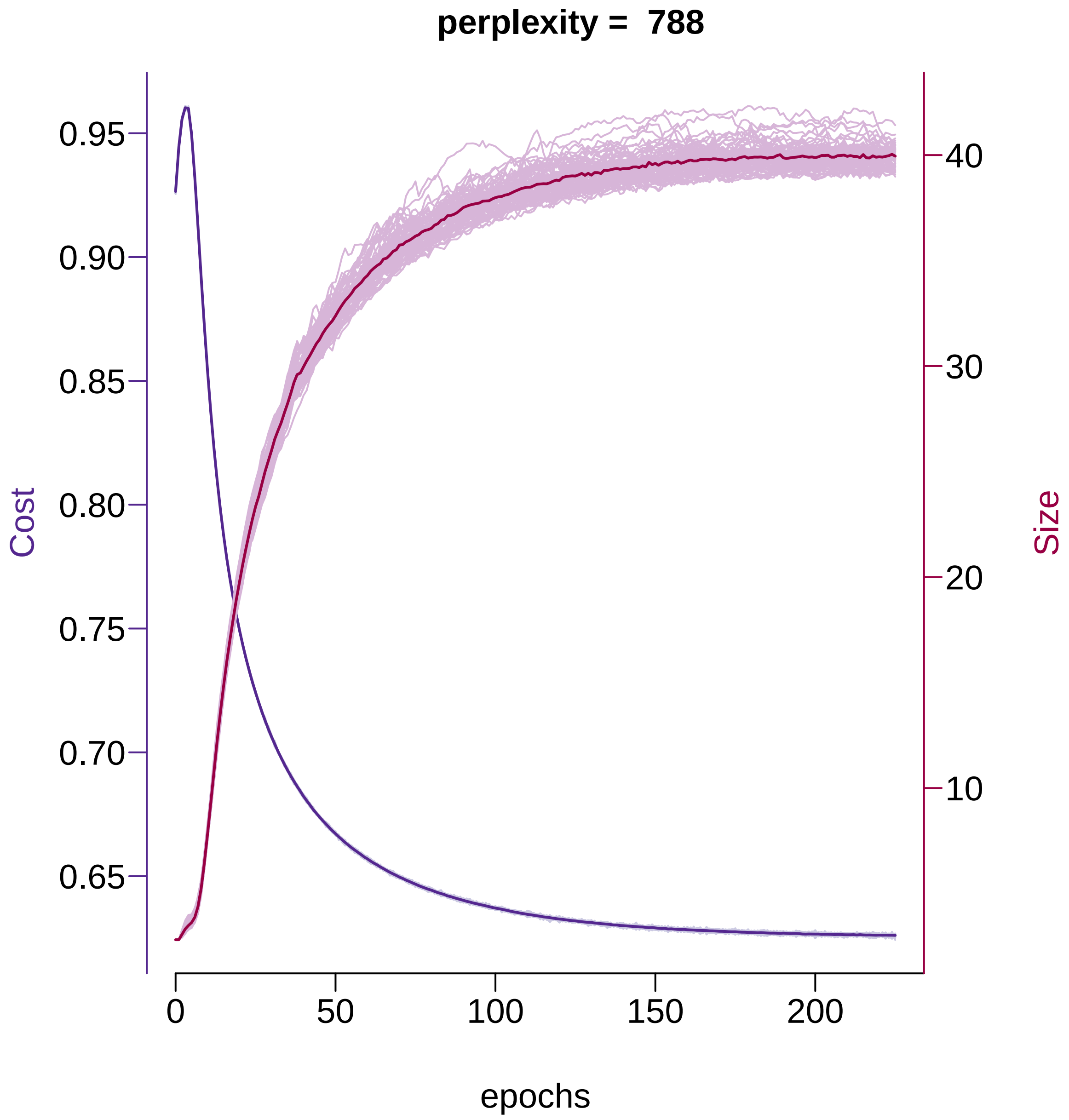

perplexity = 788
Cost
Size
epochs
0.95
0.90
0.85
0.80
0.75
0.70
0.65
40
30
20
10
0
50
100
150
200

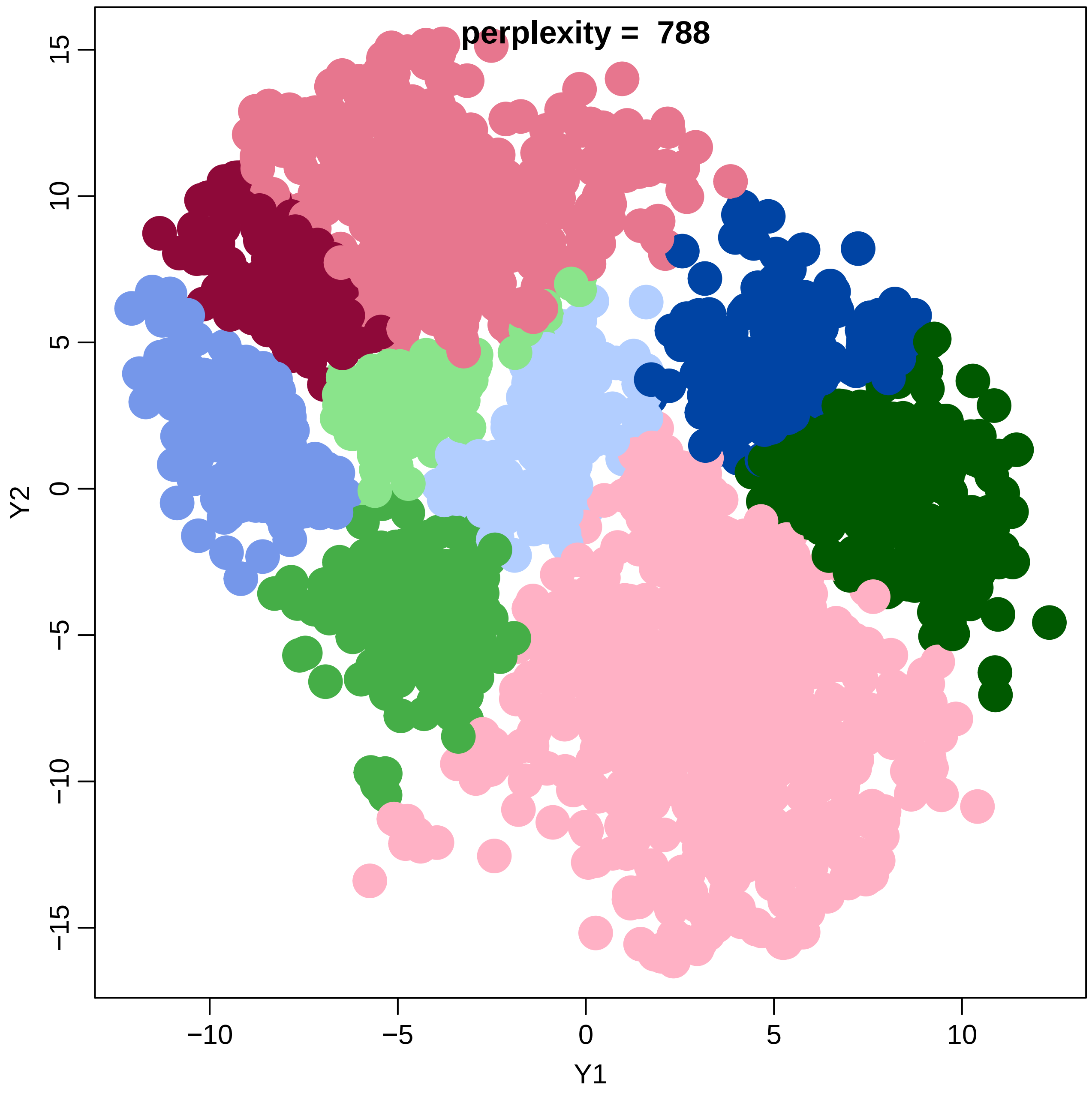
perplexity = 788
Y2
15
10
5
0
−5
−10
−15
Y1
−10
−5
0
5
10

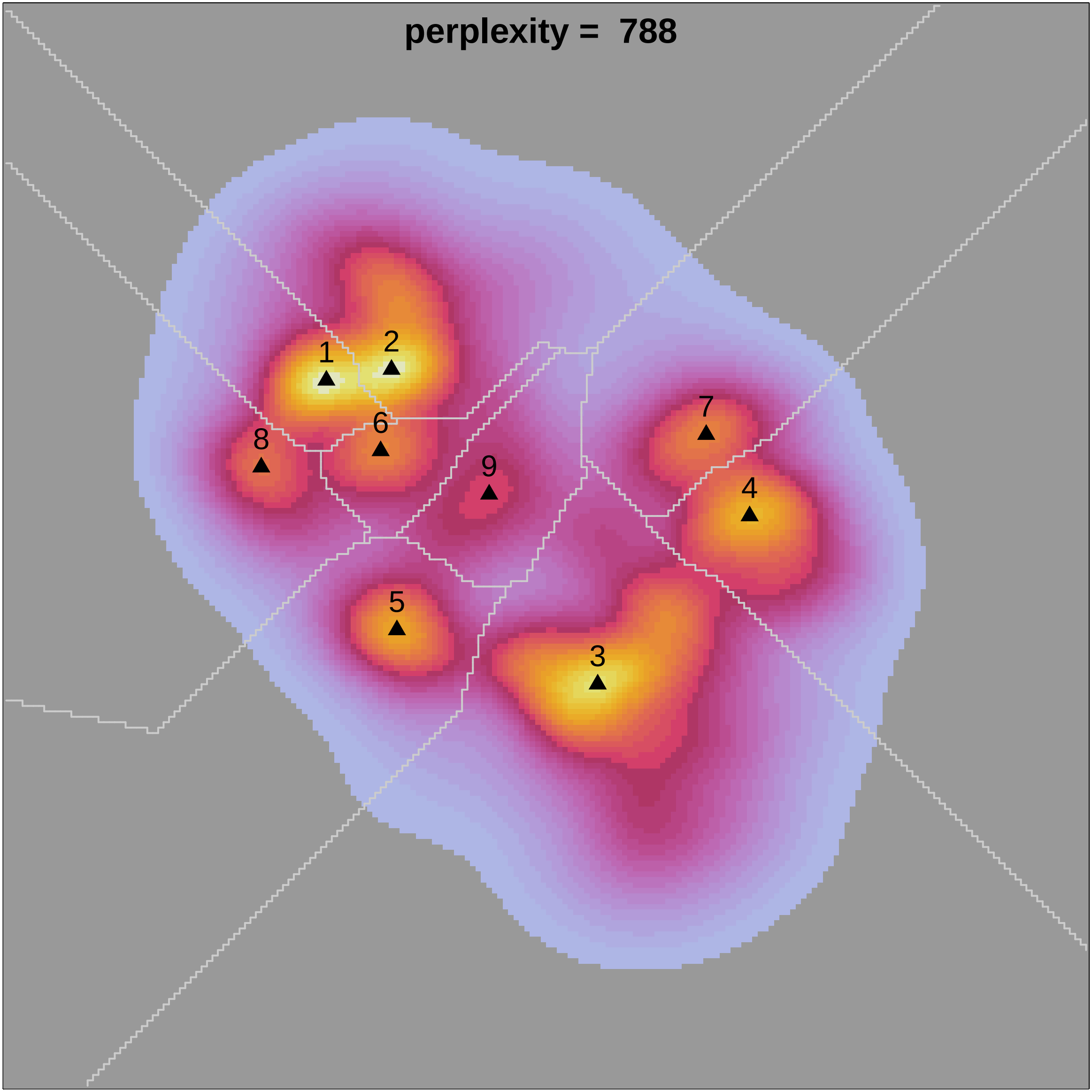
perplexity = 788
1
2
6
8
9
7
4
5
3

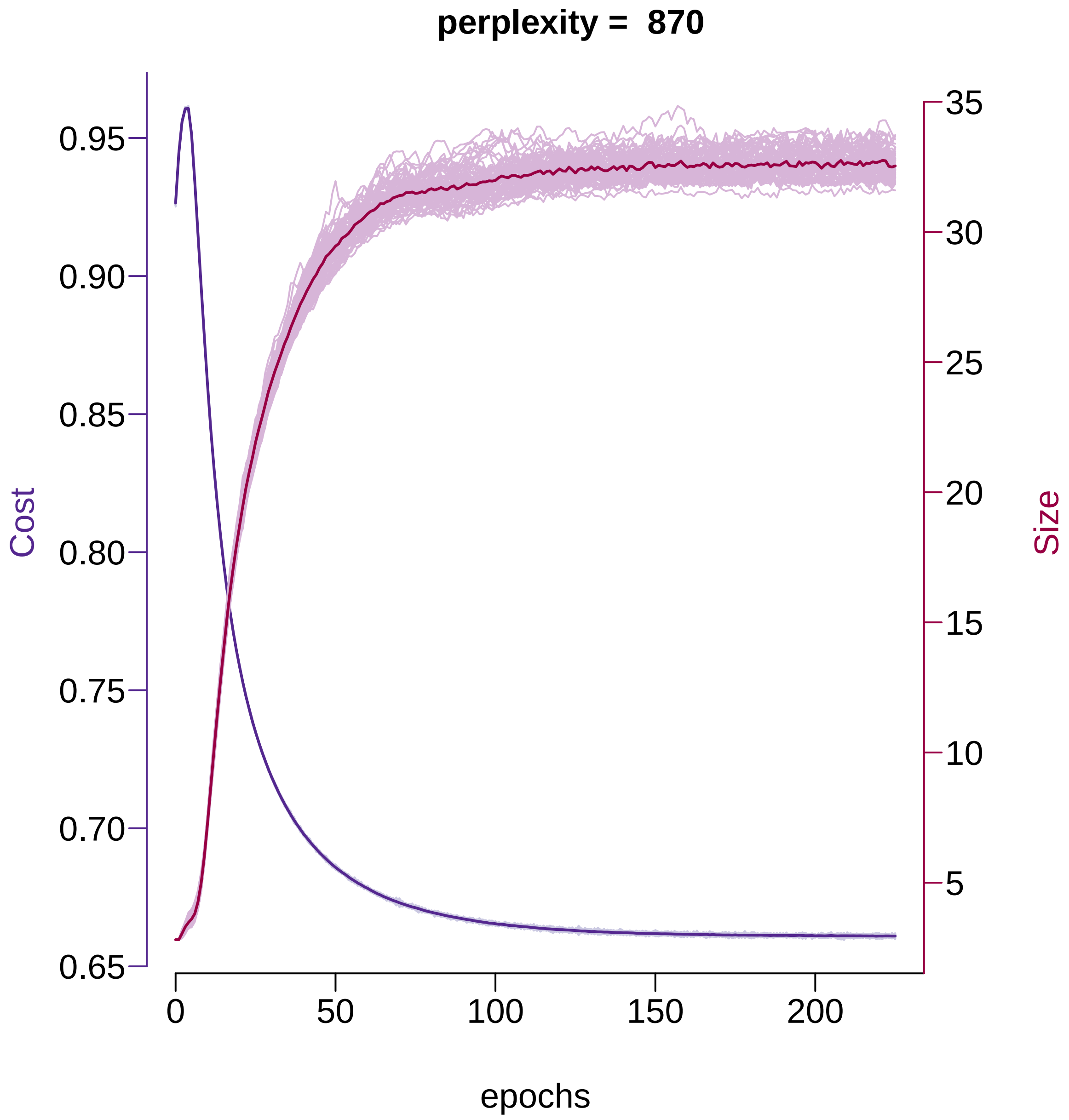
perplexity = 870
Cost
Size
epochs
0.95
0.90
0.85
0.80
0.75
0.70
0.65
35
30
25
20
15
10
5
0
50
100
150
200

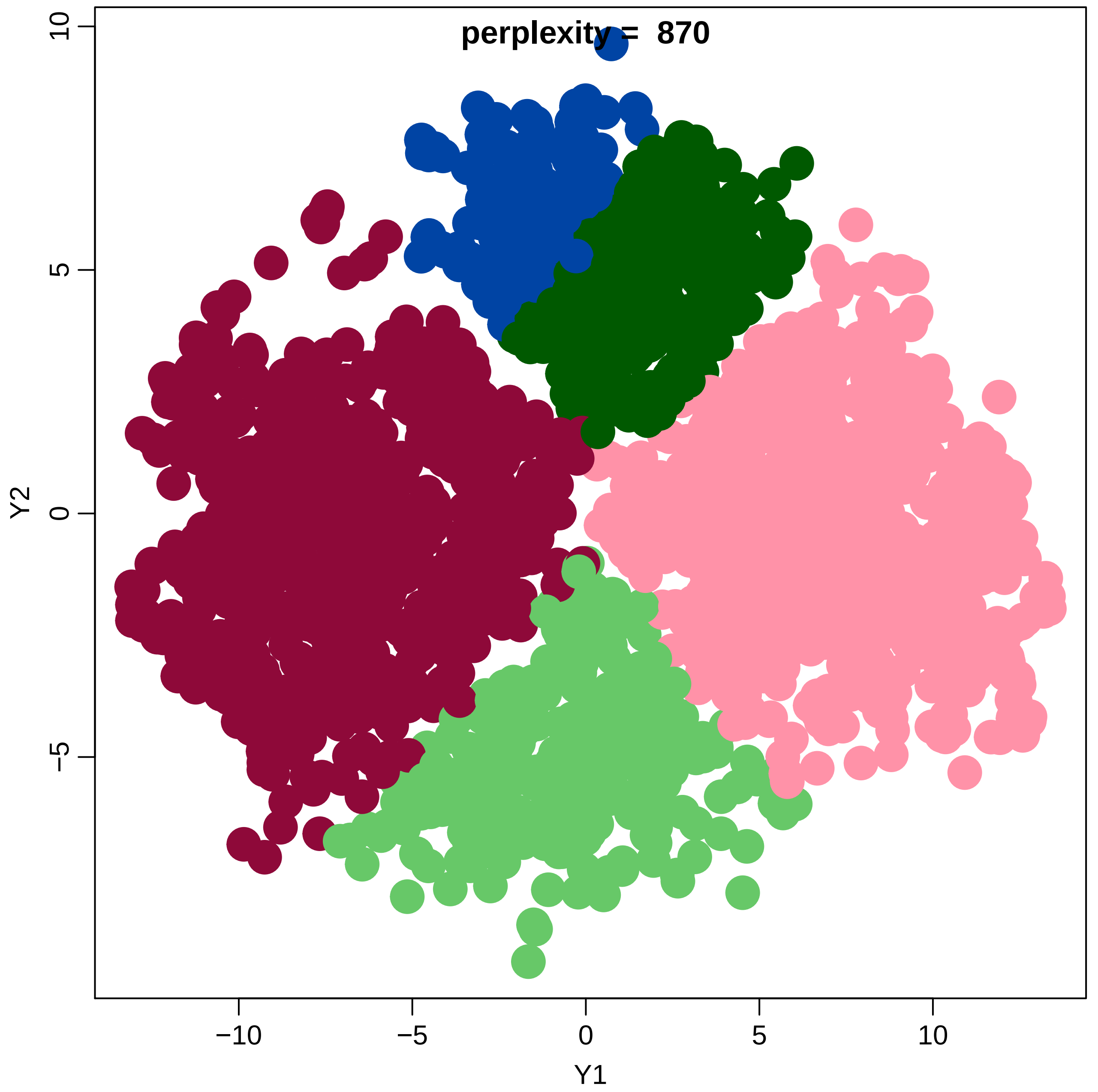

perplexity = 870
Y2
Y1
10
5
0
−5
−10
−5
0
5
10

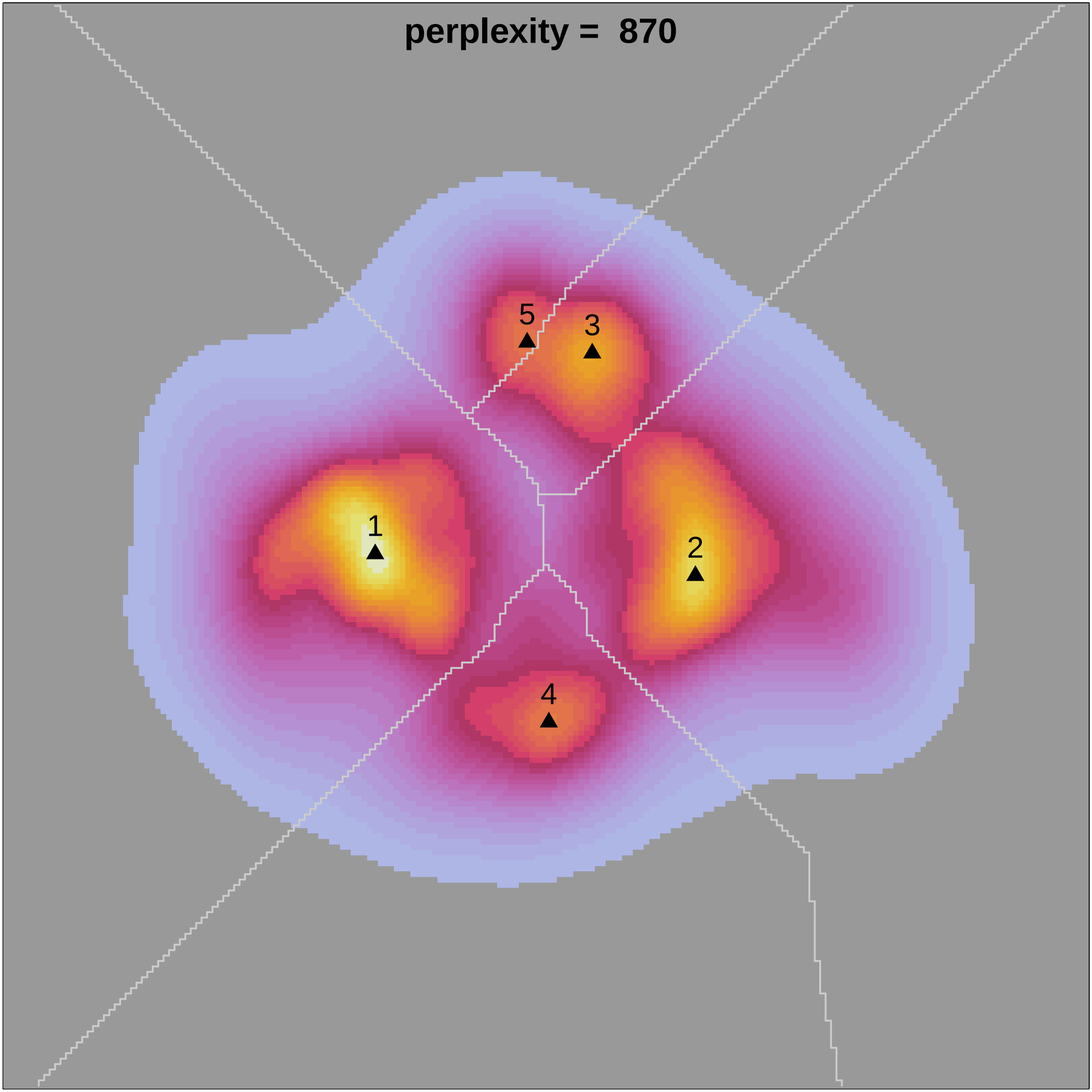
perplexity = 870
1
2
3
4
5

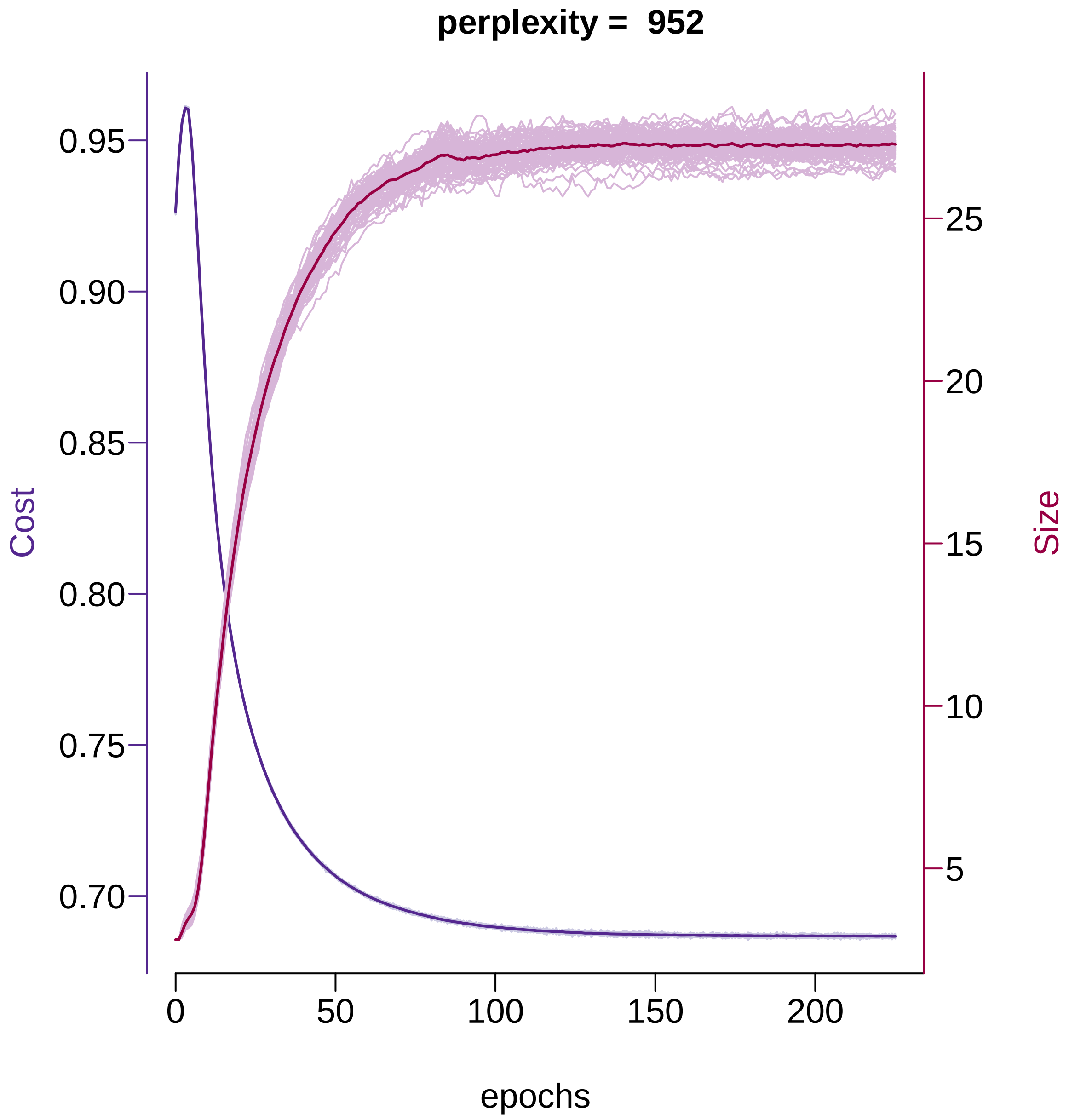

perplexity = 952
Cost
Size
epochs
0.95
0.90
0.85
0.80
0.75
0.70
25
20
15
10
5
0
50
100
150
200

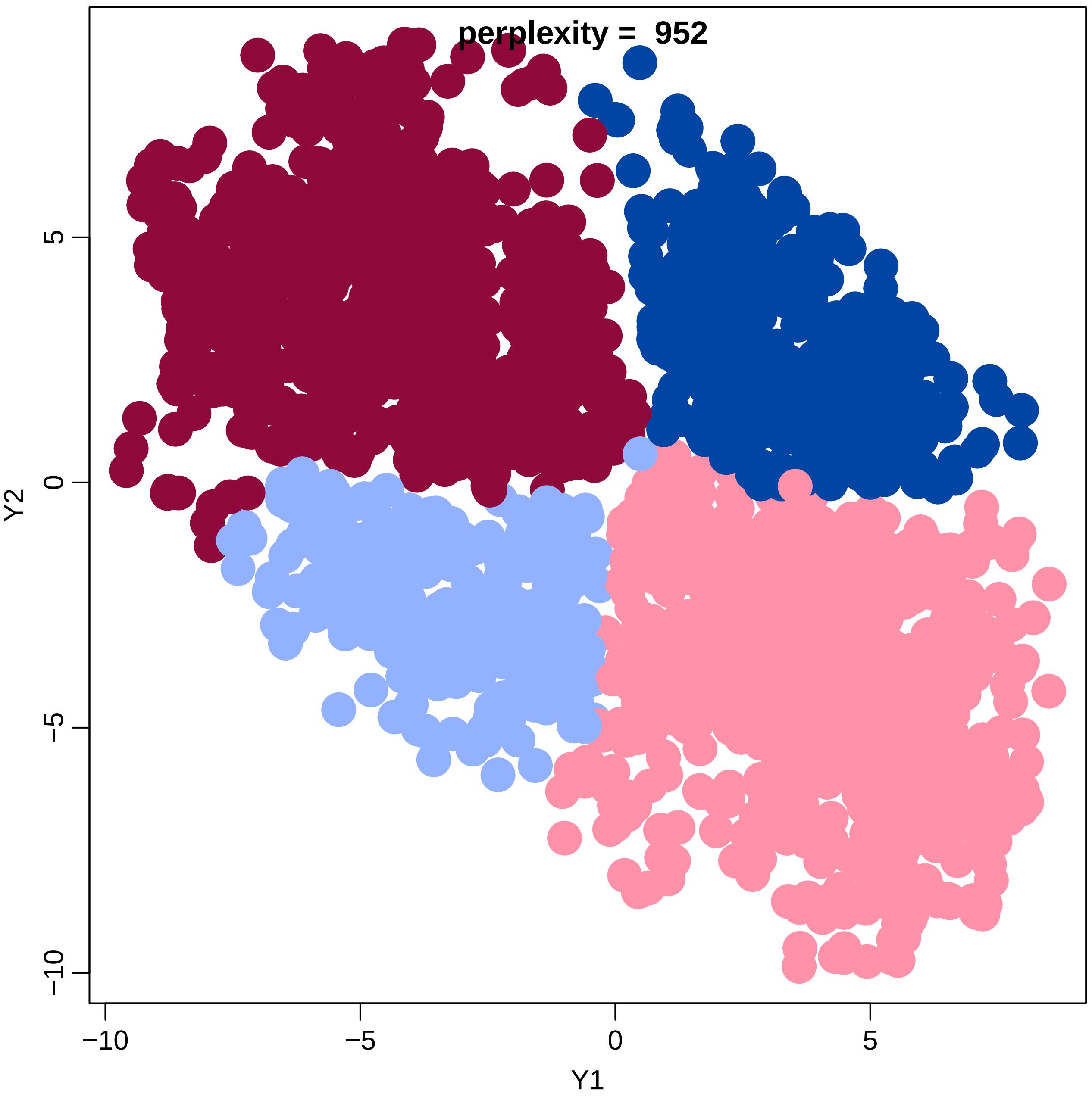
perplexity = 952
Y1
Y2
−10
−5
0
5
−10
−5
0
5

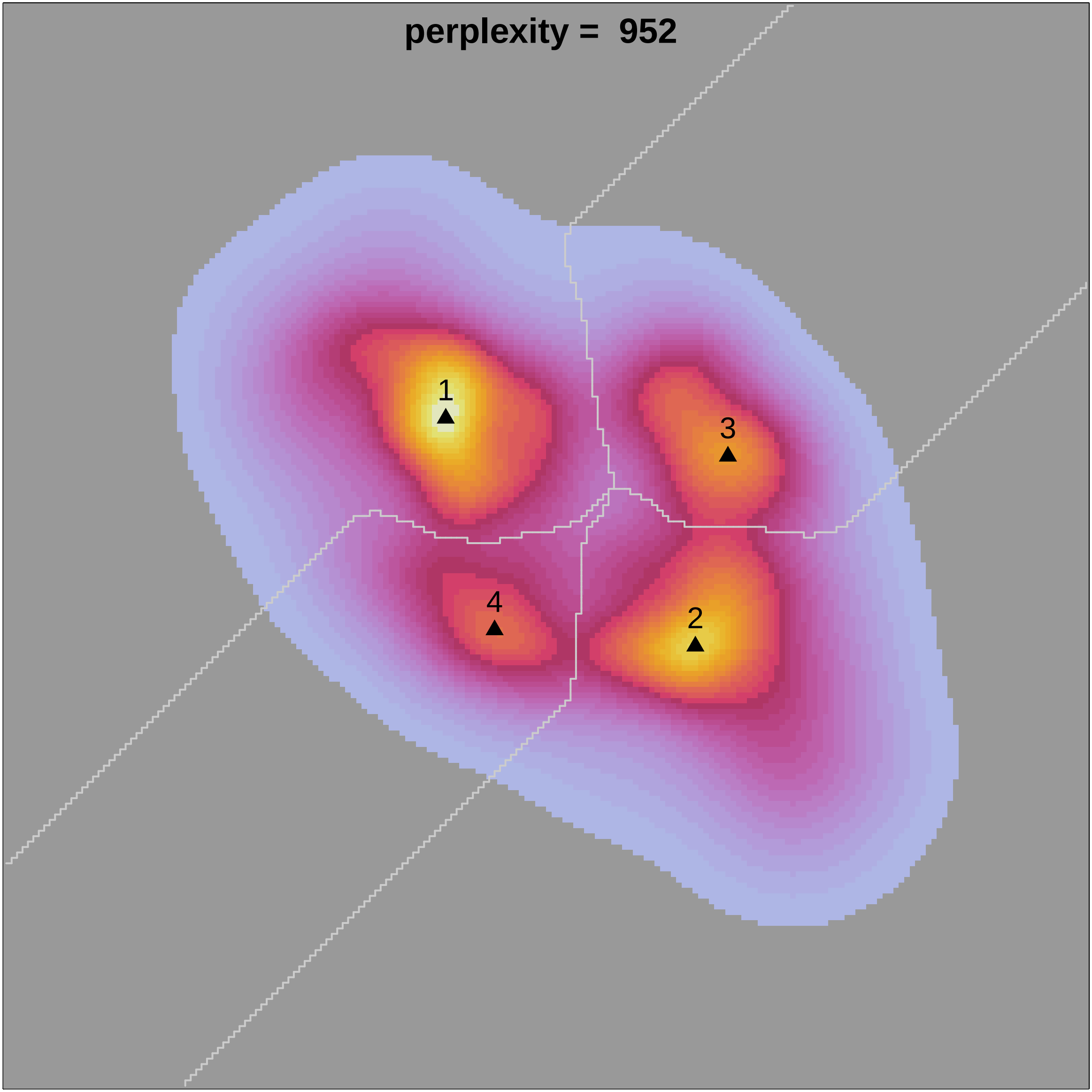
perplexity = 952
1
2
3
4

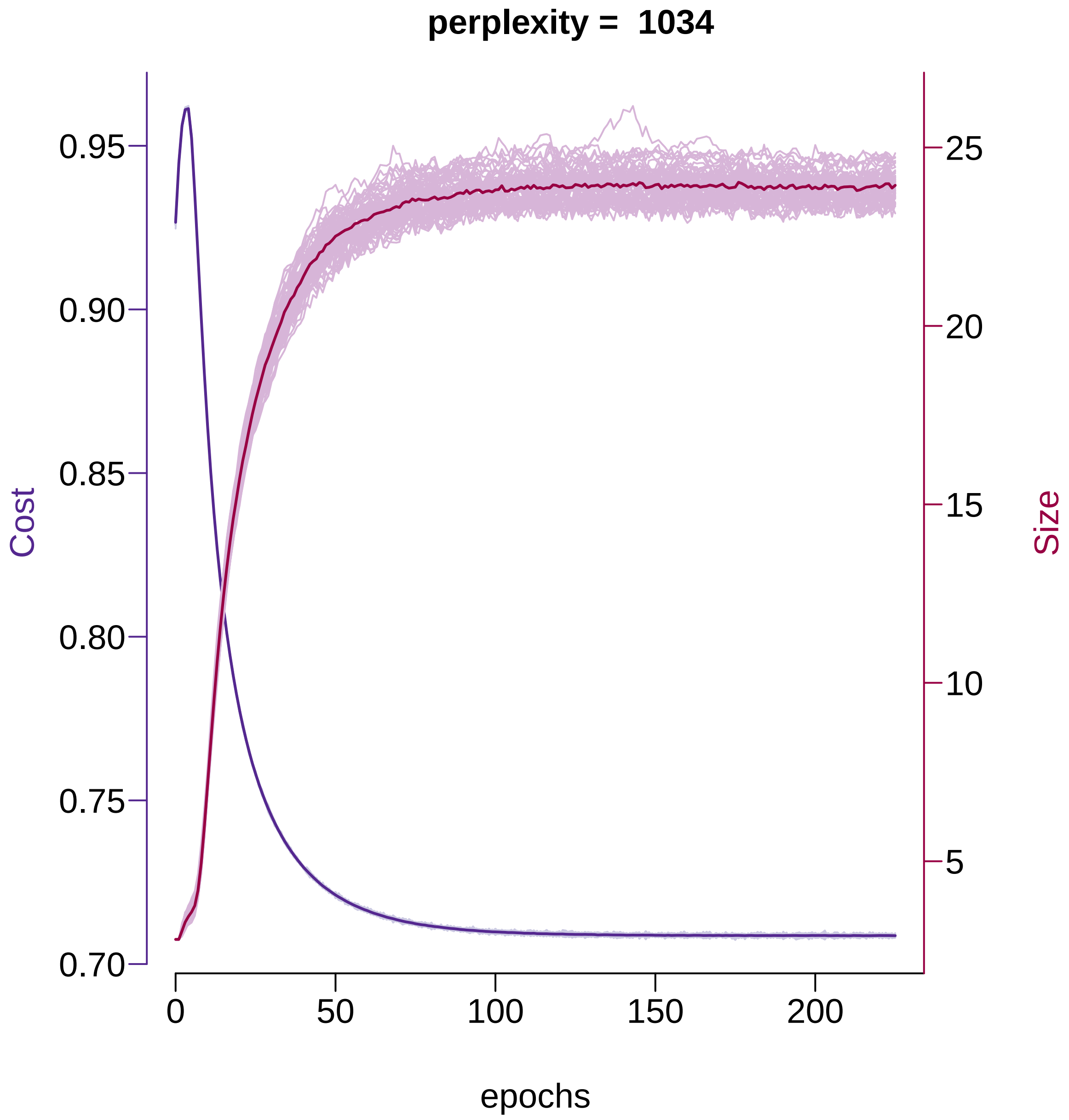

perplexity = 1034
Cost
0.95
0.90
0.85
0.80
0.75
0.70
Size
25
20
15
10
5
0
50
100
150
200
epochs

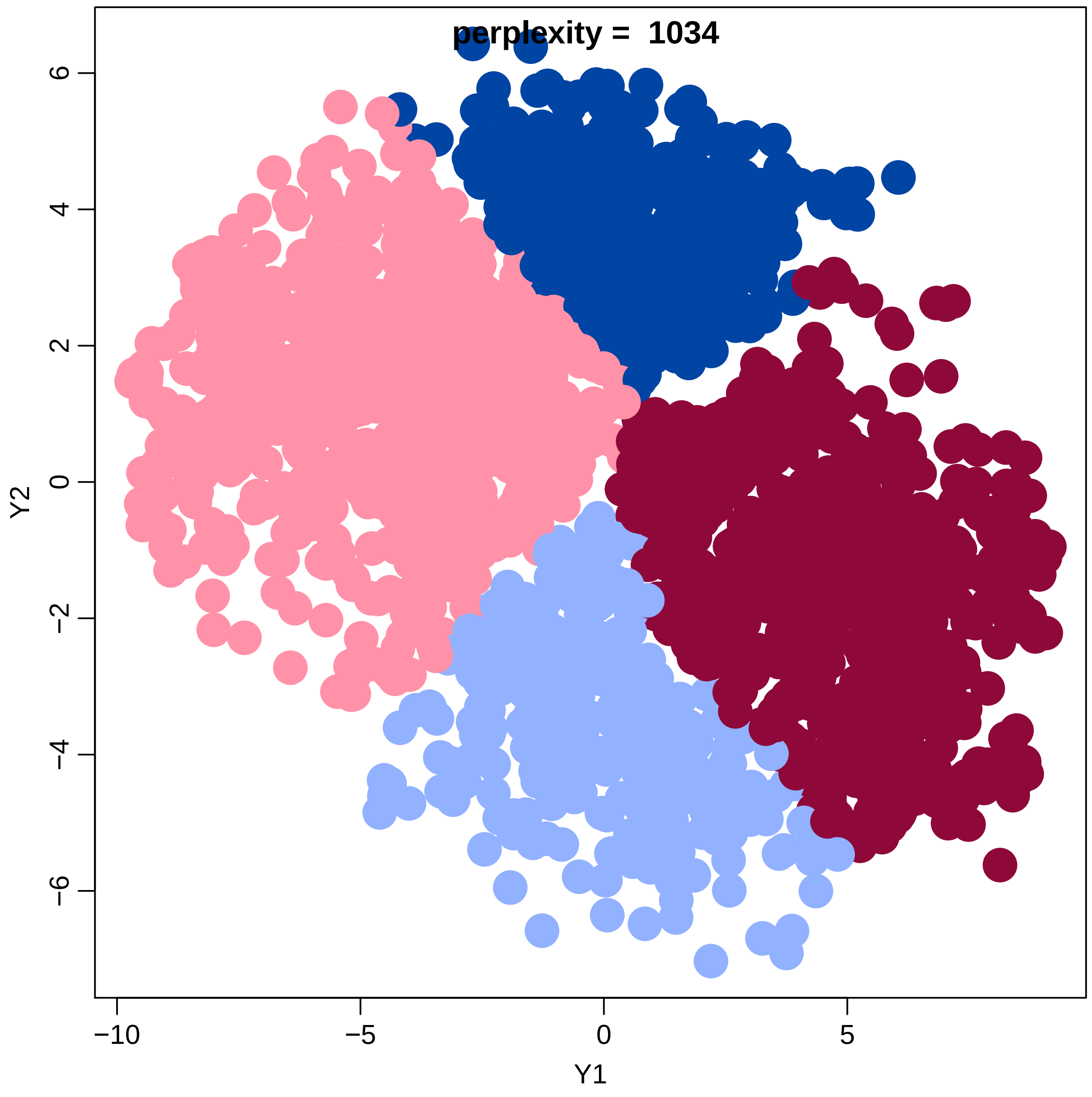
perplexity = 1034
Y2
Y1
6
4
2
0
−2
−4
−6
−10
−5
0
5

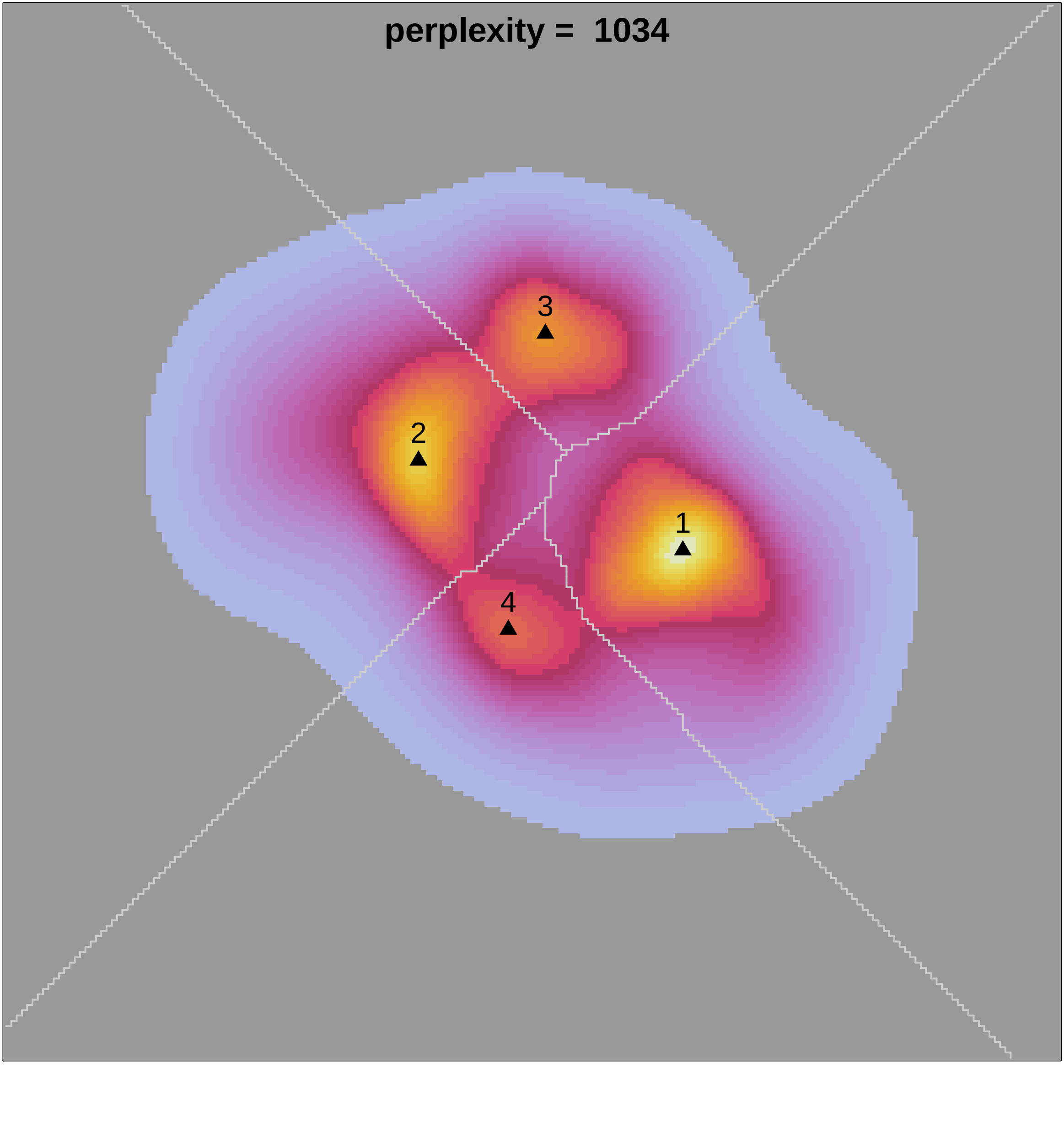
perplexity = 1034
3
2
1
4

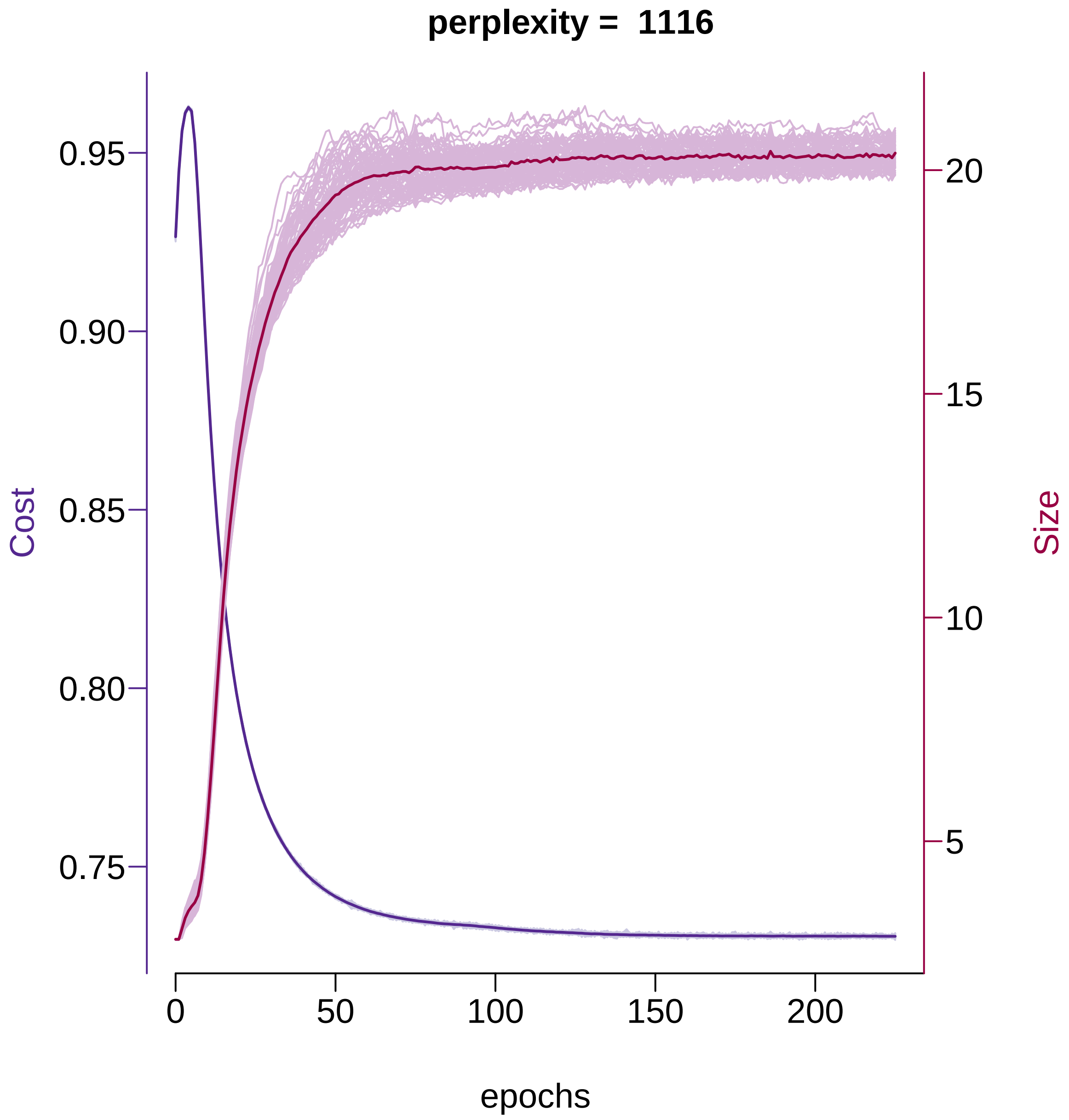
perplexity = 1116
Cost
0.95
0.90
0.85
0.80
0.75
Size
20
15
10
5
0
50
100
150
200
epochs

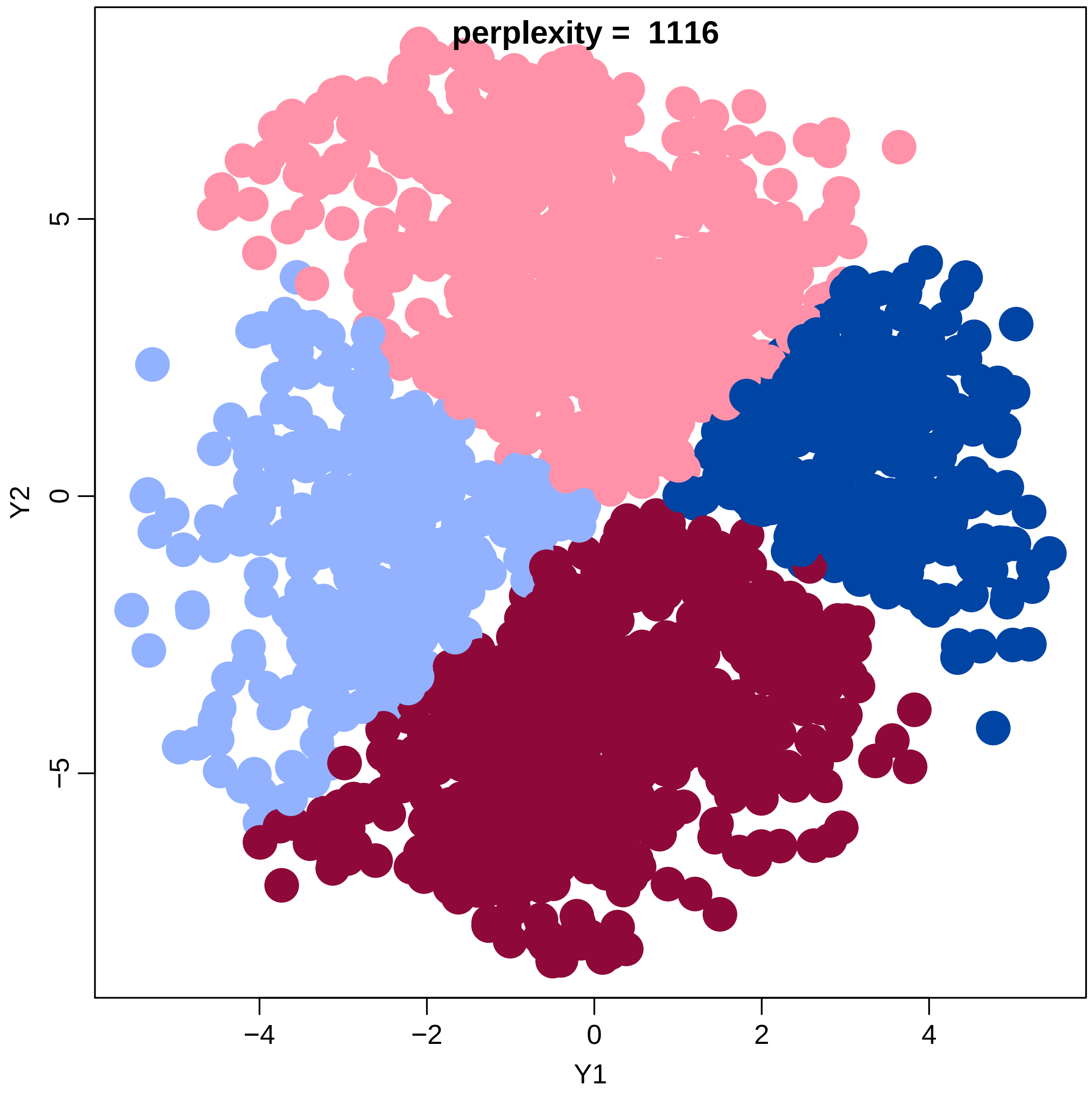
perplexity = 1116
Y2
5
0
−5
−4
−2
0
2
4
Y1

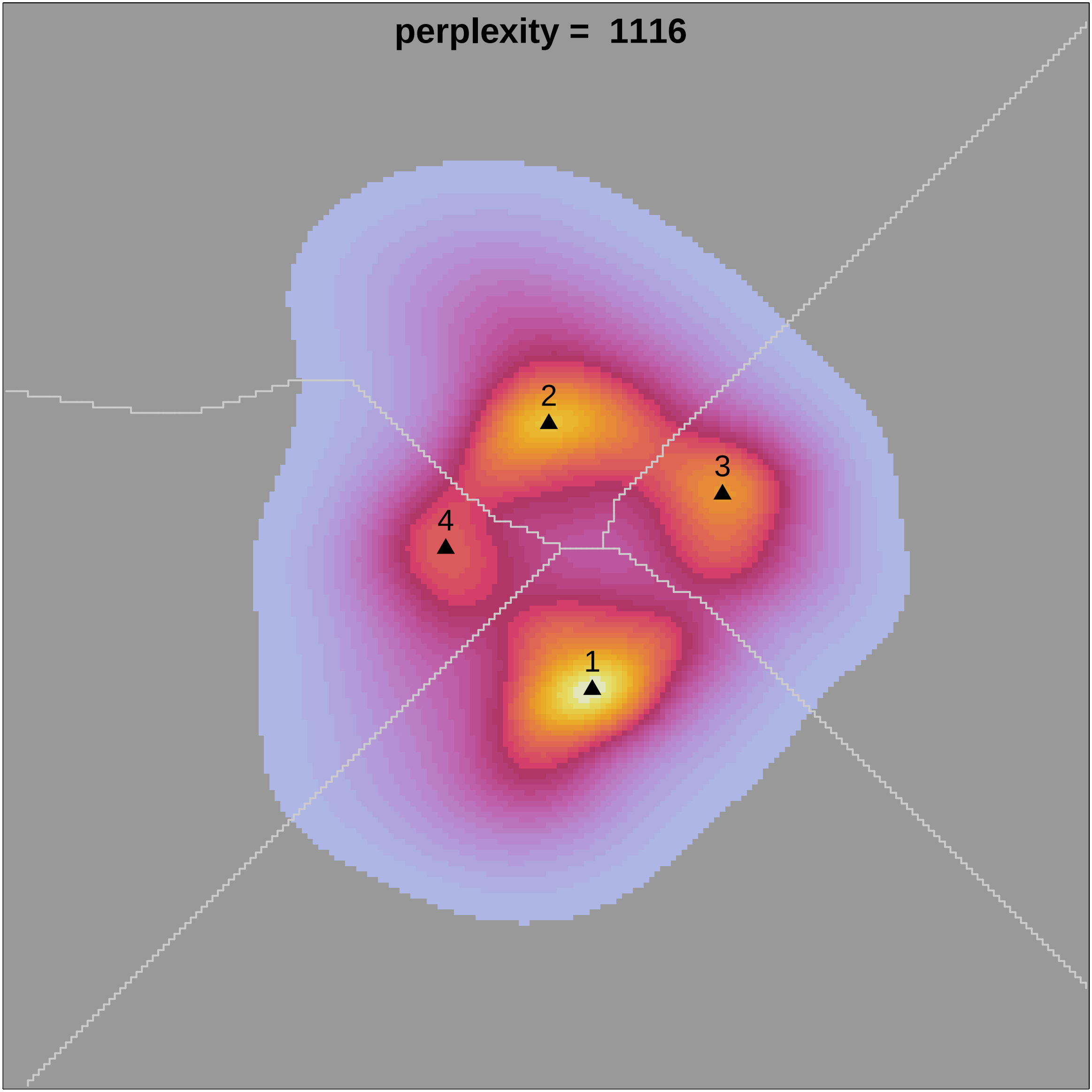
perplexity = 1116
2
3
4
1

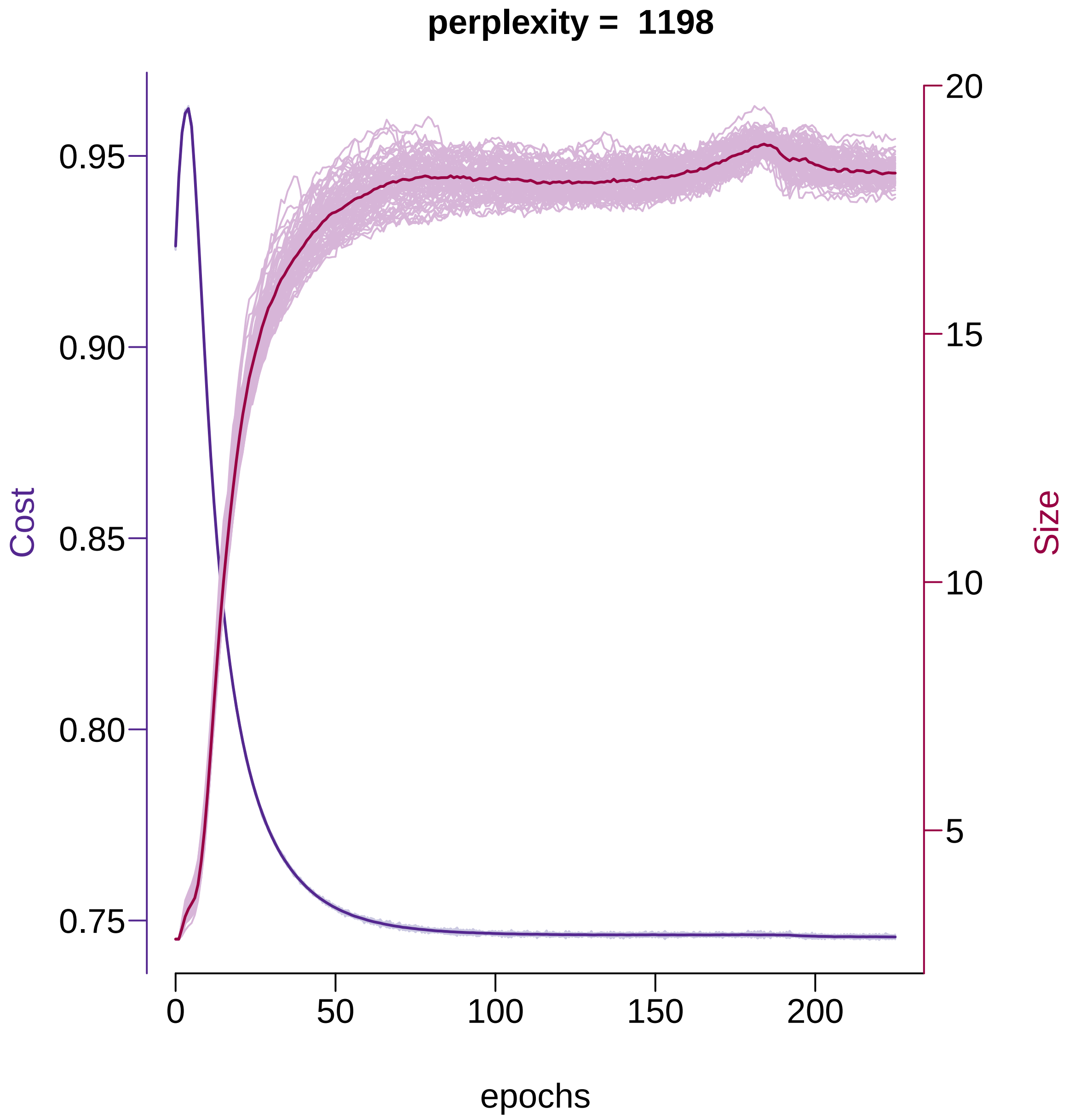
perplexity = 1198
Cost
0.95
0.90
0.85
0.80
0.75
Size
20
15
10
5
0
50
100
150
200
epochs

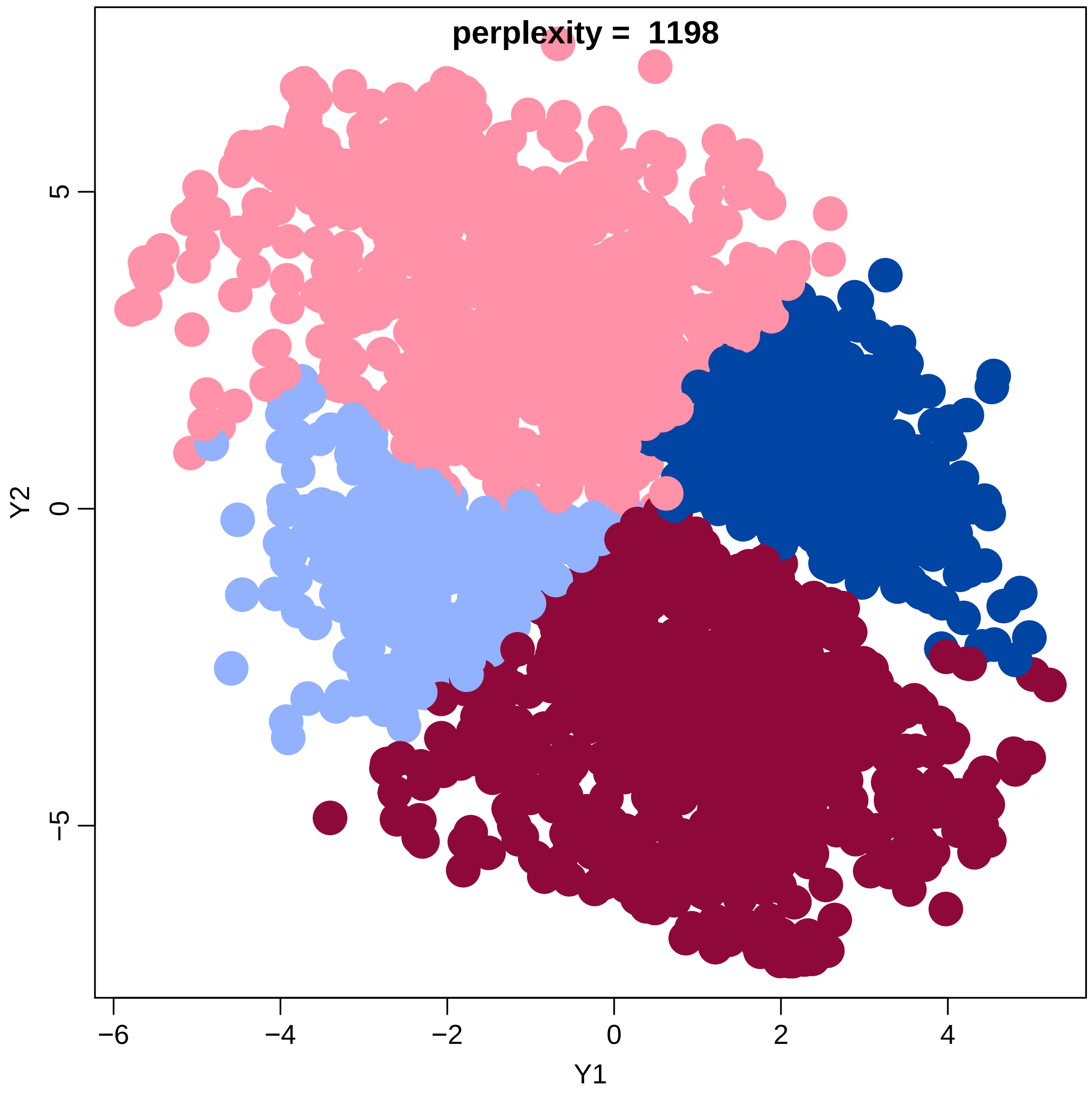
perplexity = 1198
Y2
5
0
−5
−6
−4
−2
0
2
4
Y1

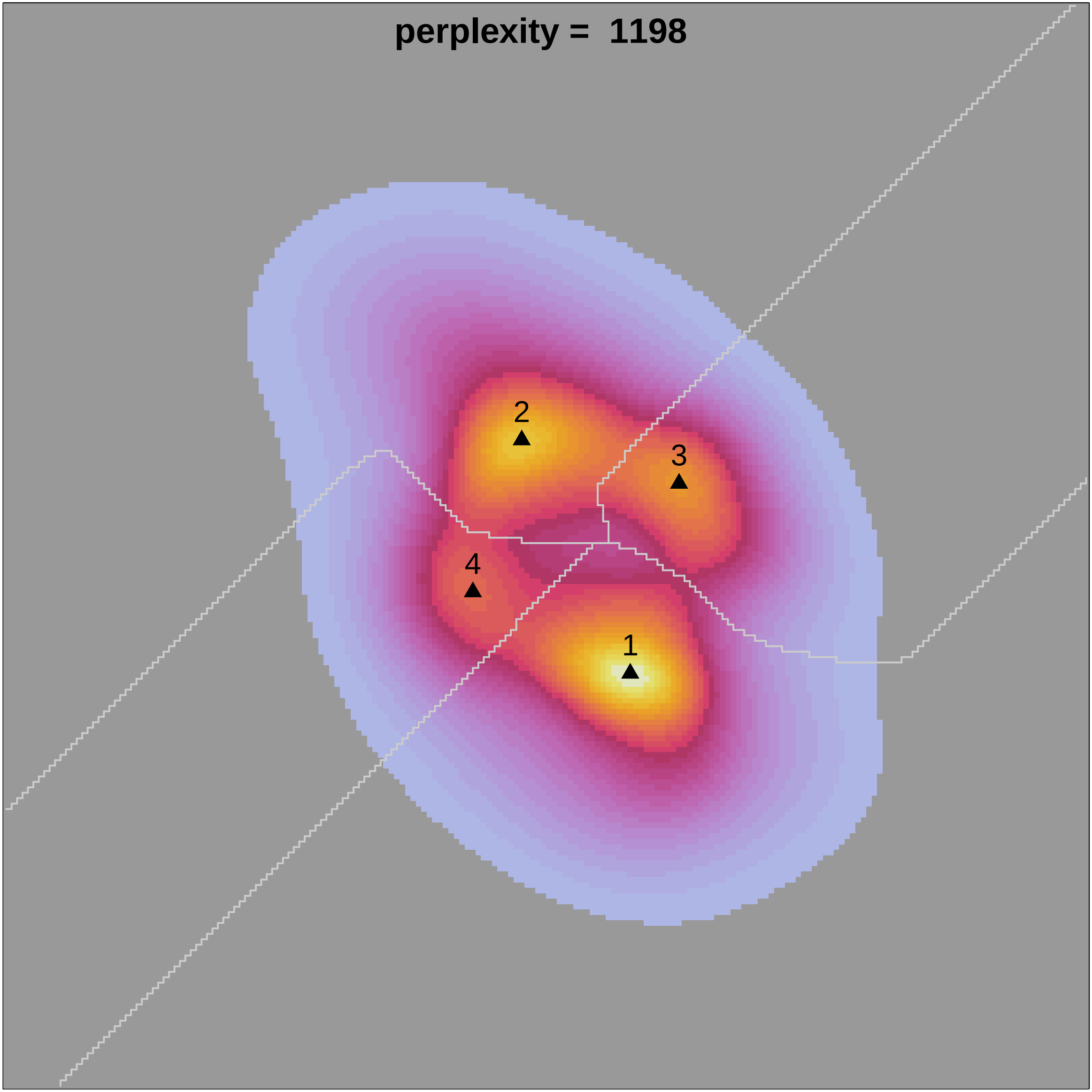
perplexity = 1198
2
3
4
1

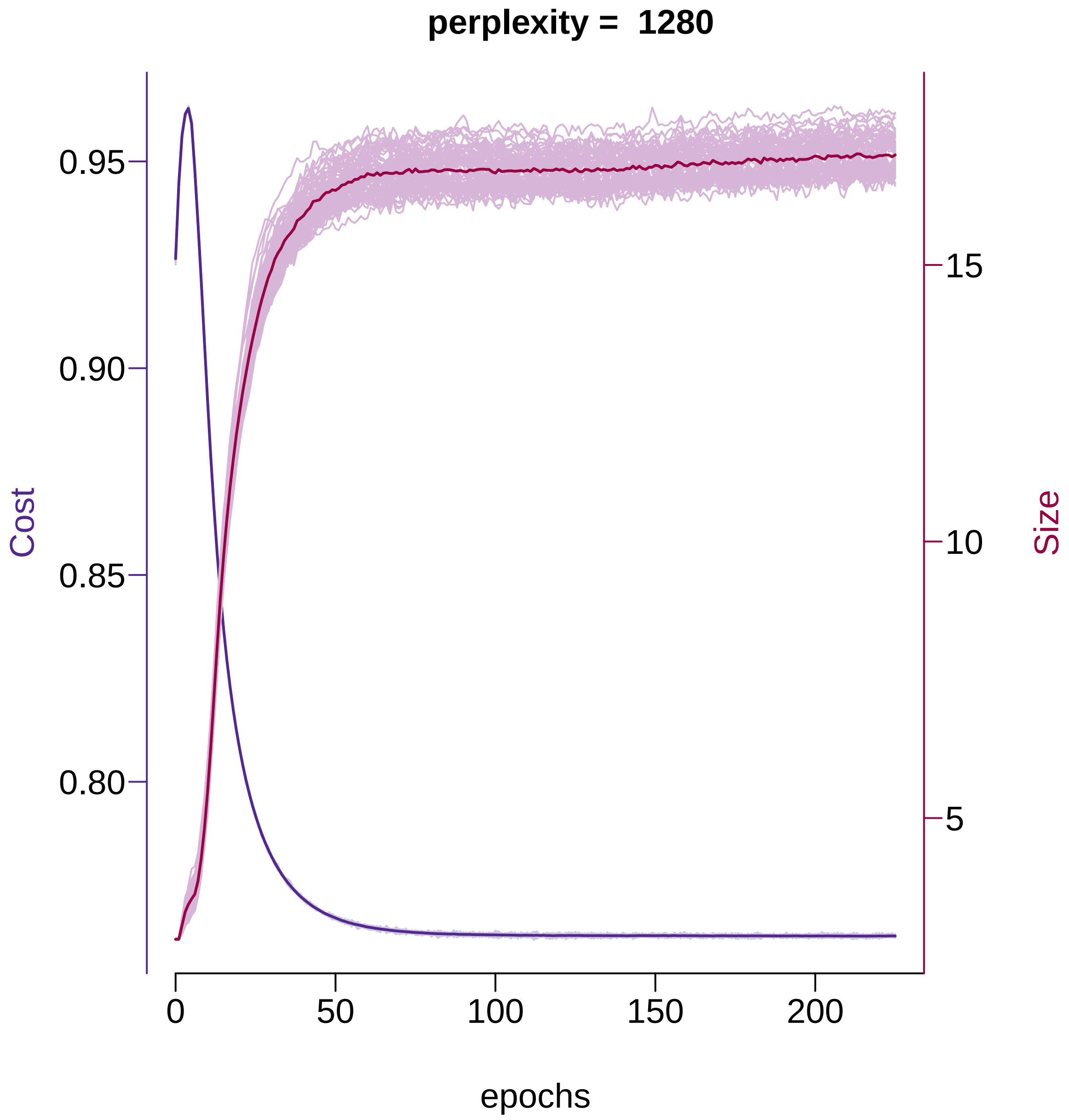
perplexity = 1280
Cost
0.95
0.90
0.85
0.80
Size
15
10
5
0
50
100
150
200
epochs

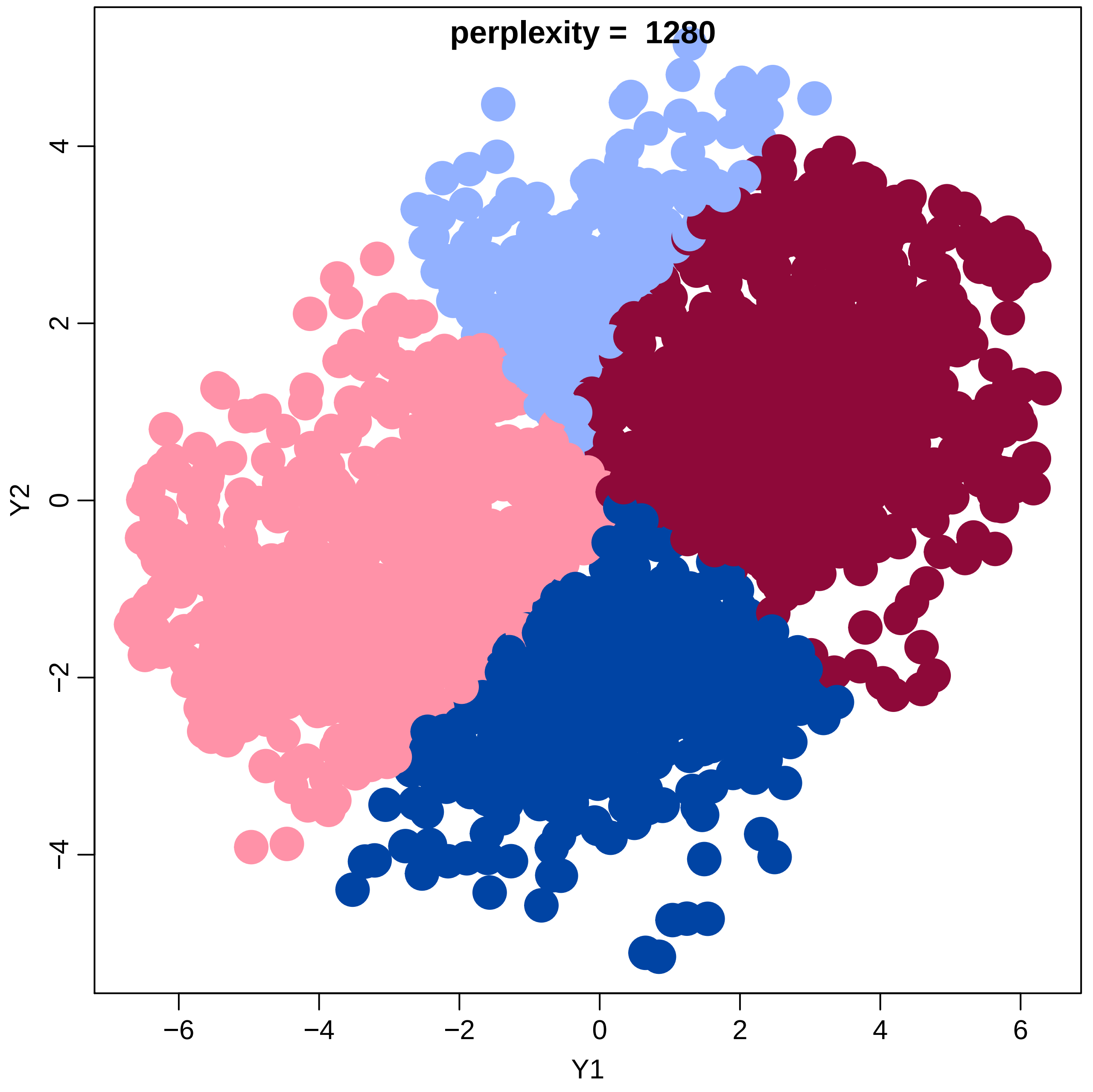
perplexity = 1280
Y2
4
2
0
−2
−4
Y1
−6
−4
−2
0
2
4
6

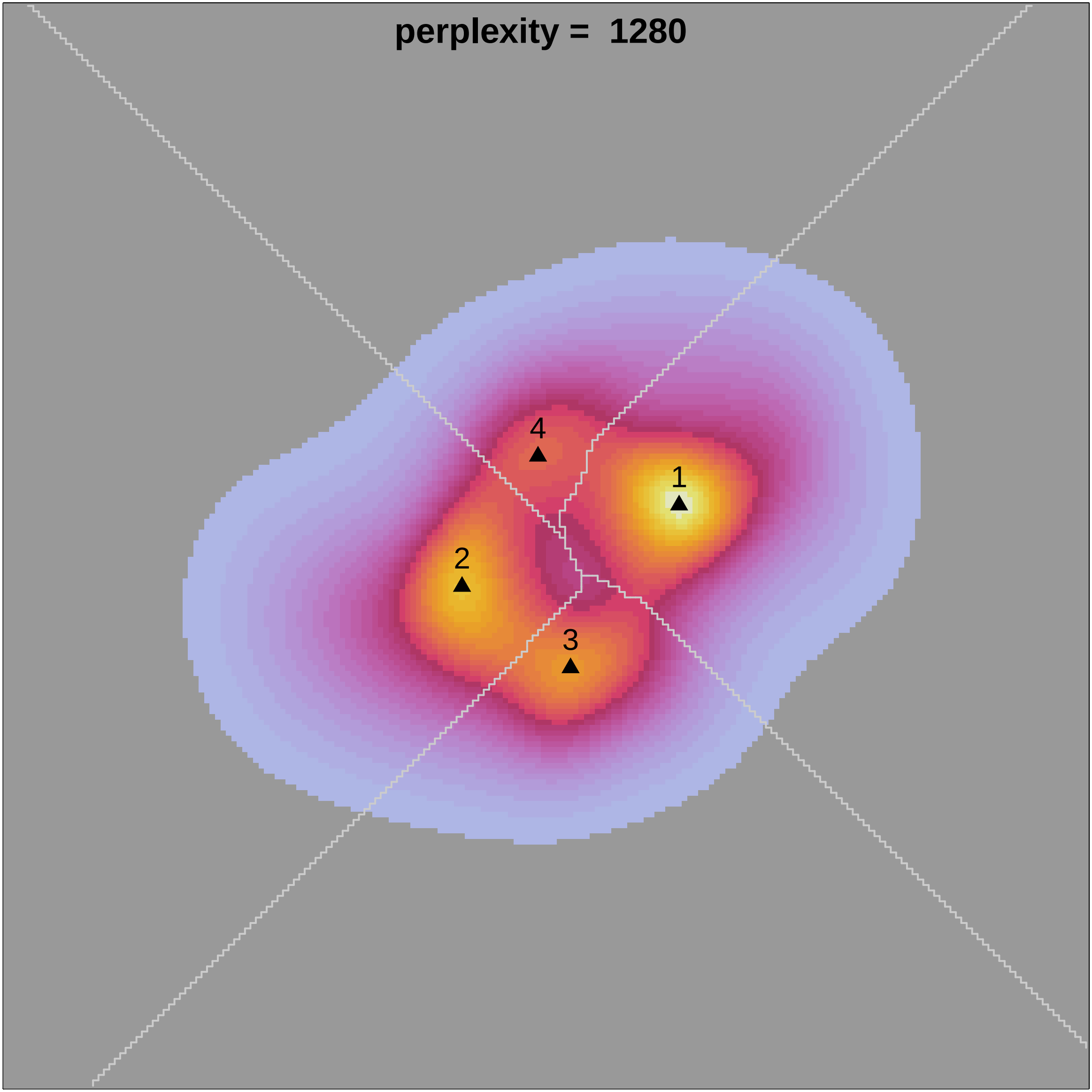

perplexity = 1280
1
2
3
4

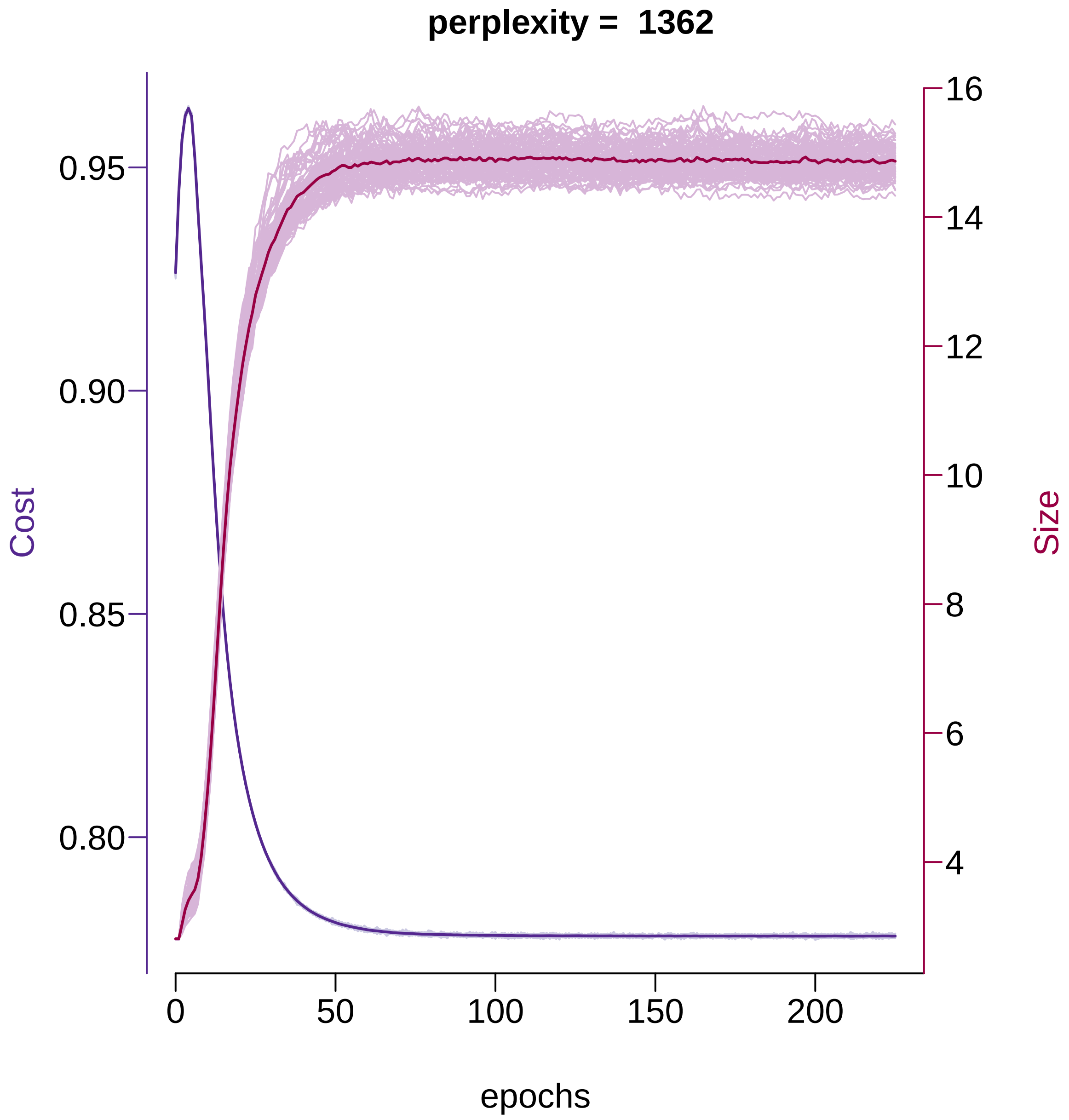
perplexity = 1362
Cost
0.95
0.90
0.85
0.80
Size
16
14
12
10
8
6
4
0
50
100
150
200
epochs

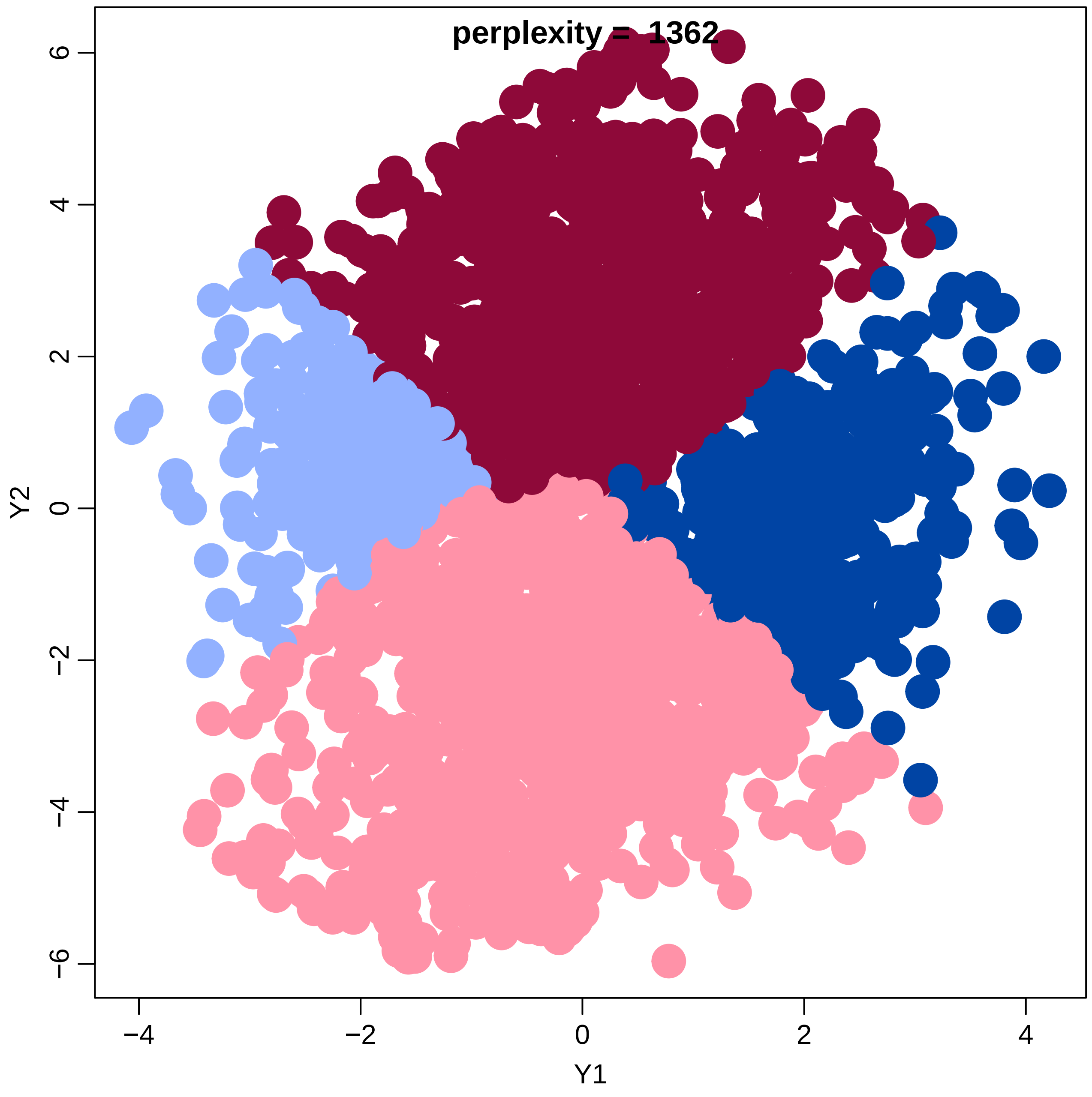

perplexity = 1362
6
4
2
0
−2
−4
−6
Y2
−4
−2
0
2
4
Y1

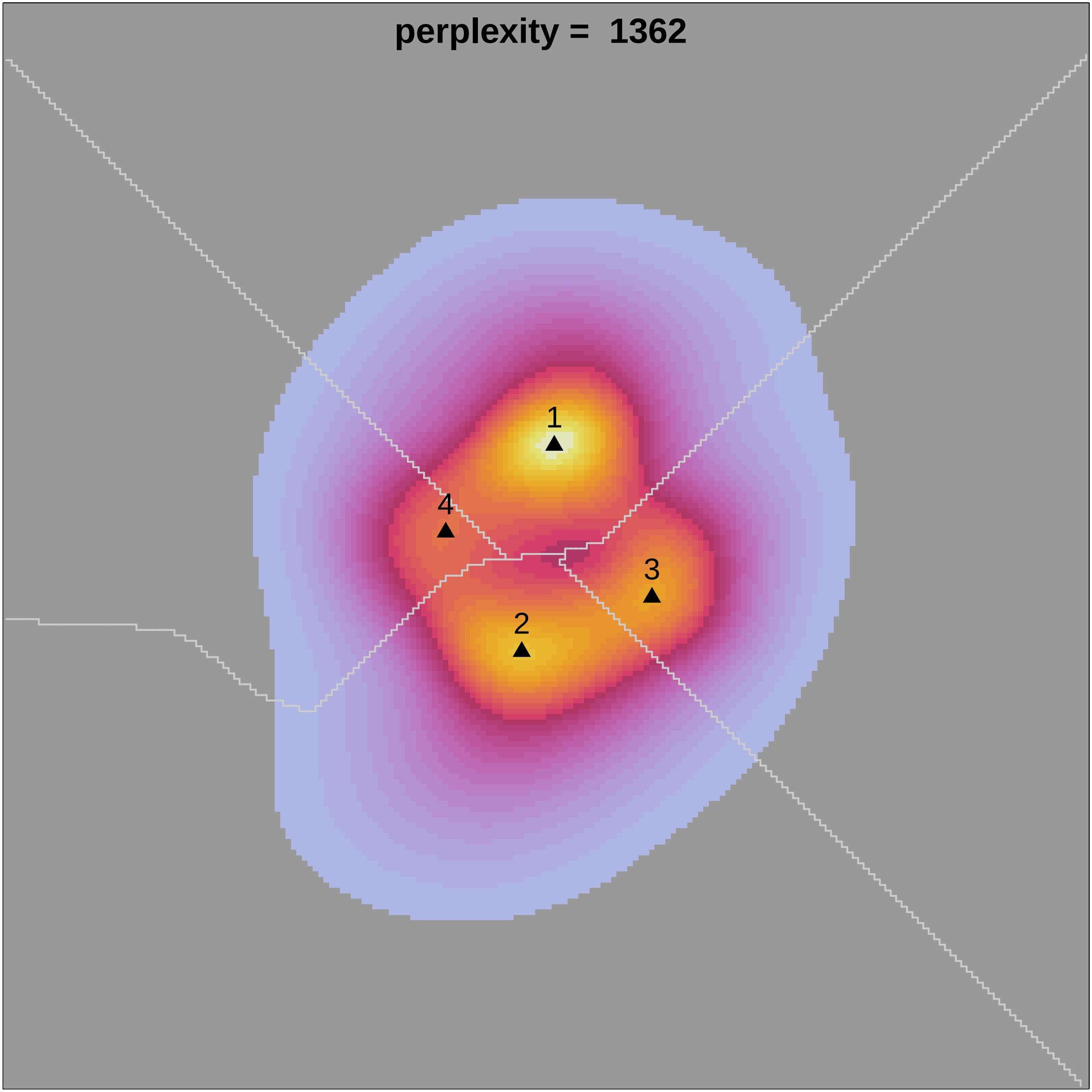
perplexity = 1362
1
2
3
4

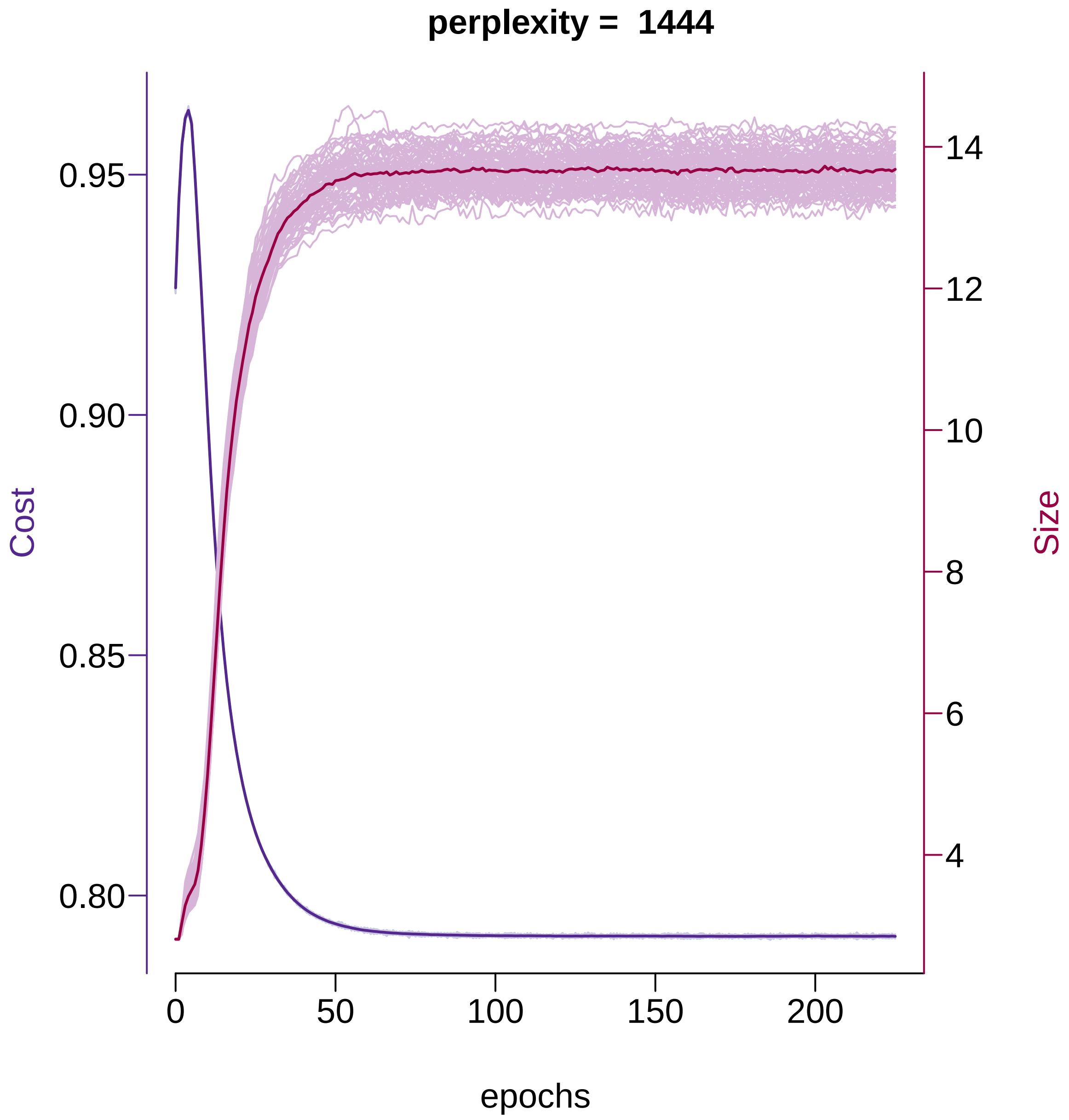

perplexity = 1444
Cost
0.95
0.90
0.85
0.80
Size
14
12
10
8
6
4
0
50
100
150
200
epochs

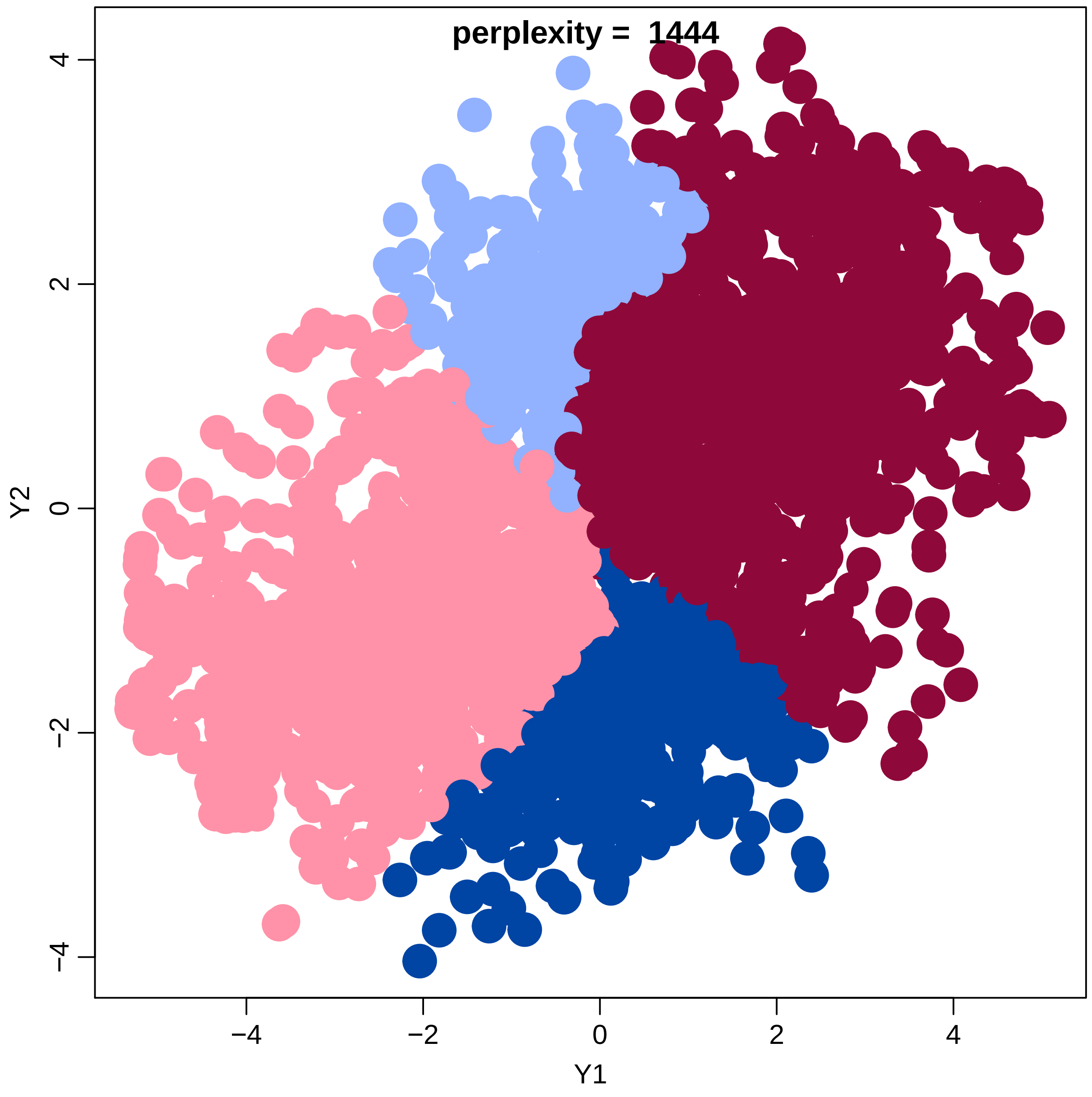
perplexity = 1444
Y2
Y1
4
2
0
−2
−4
−4
−2
0
2
4

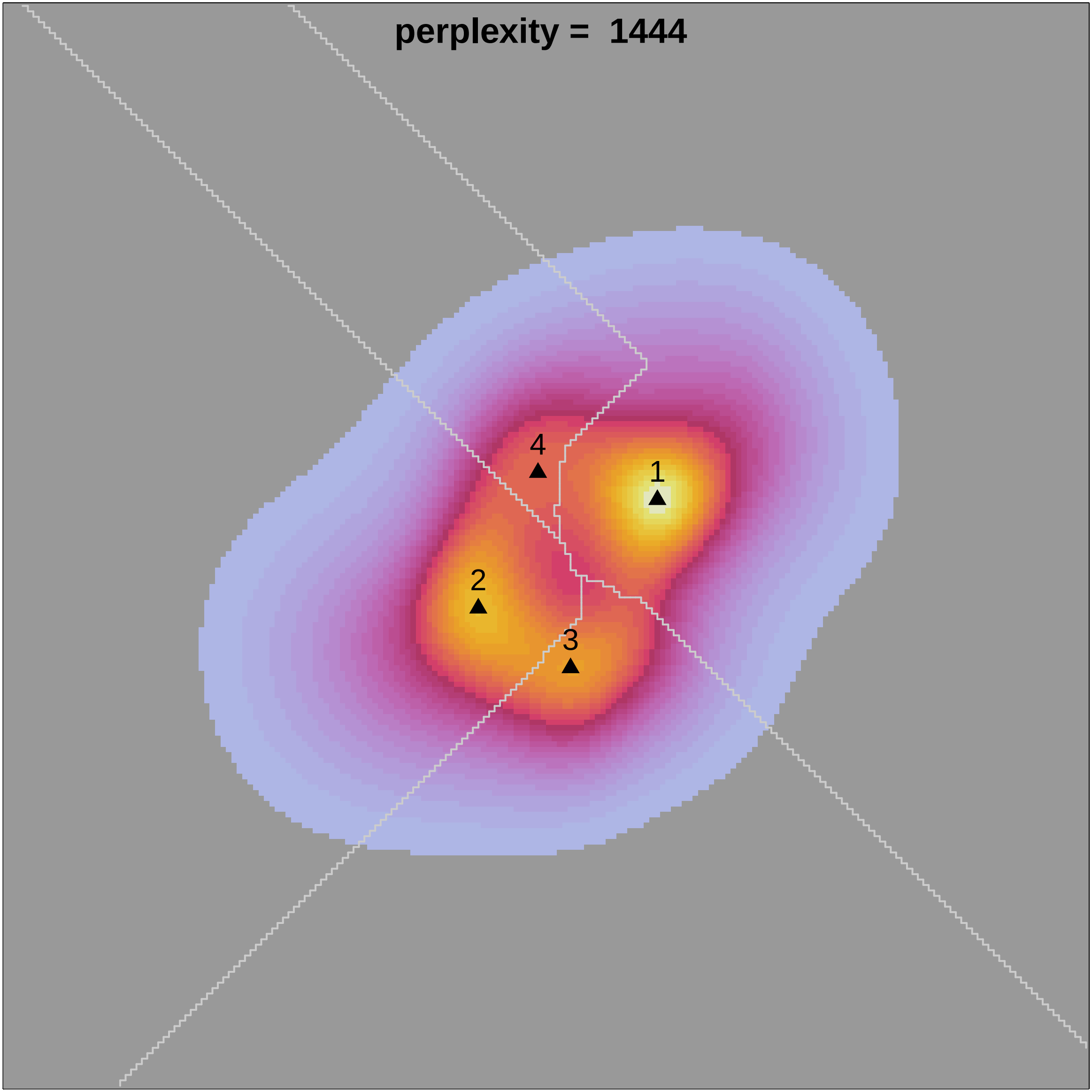
perplexity = 1444
4
1
2
3

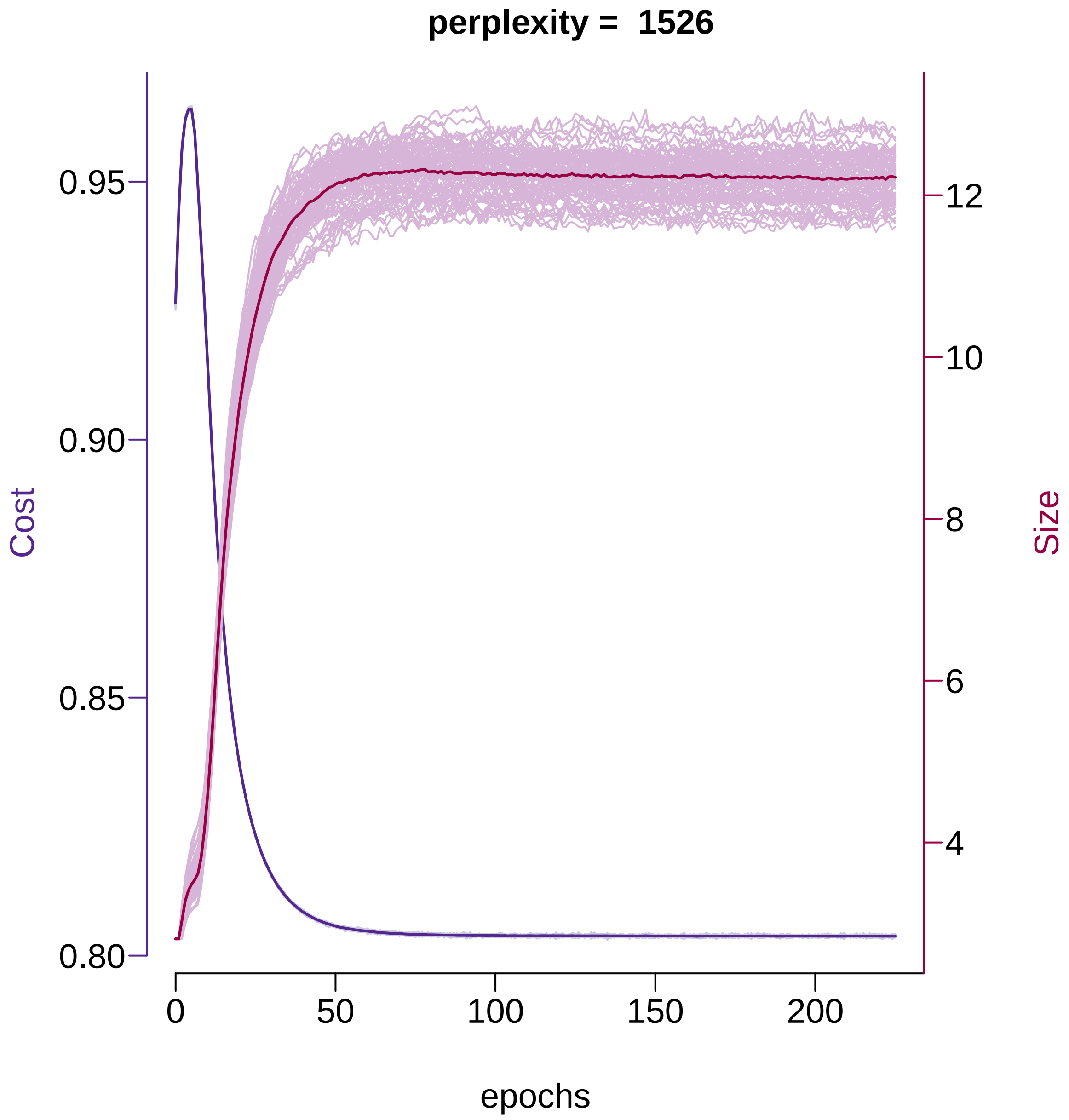
perplexity = 1526
Cost
0.95
0.90
0.85
0.80
Size
12
10
8
6
4
0
50
100
150
200
epochs

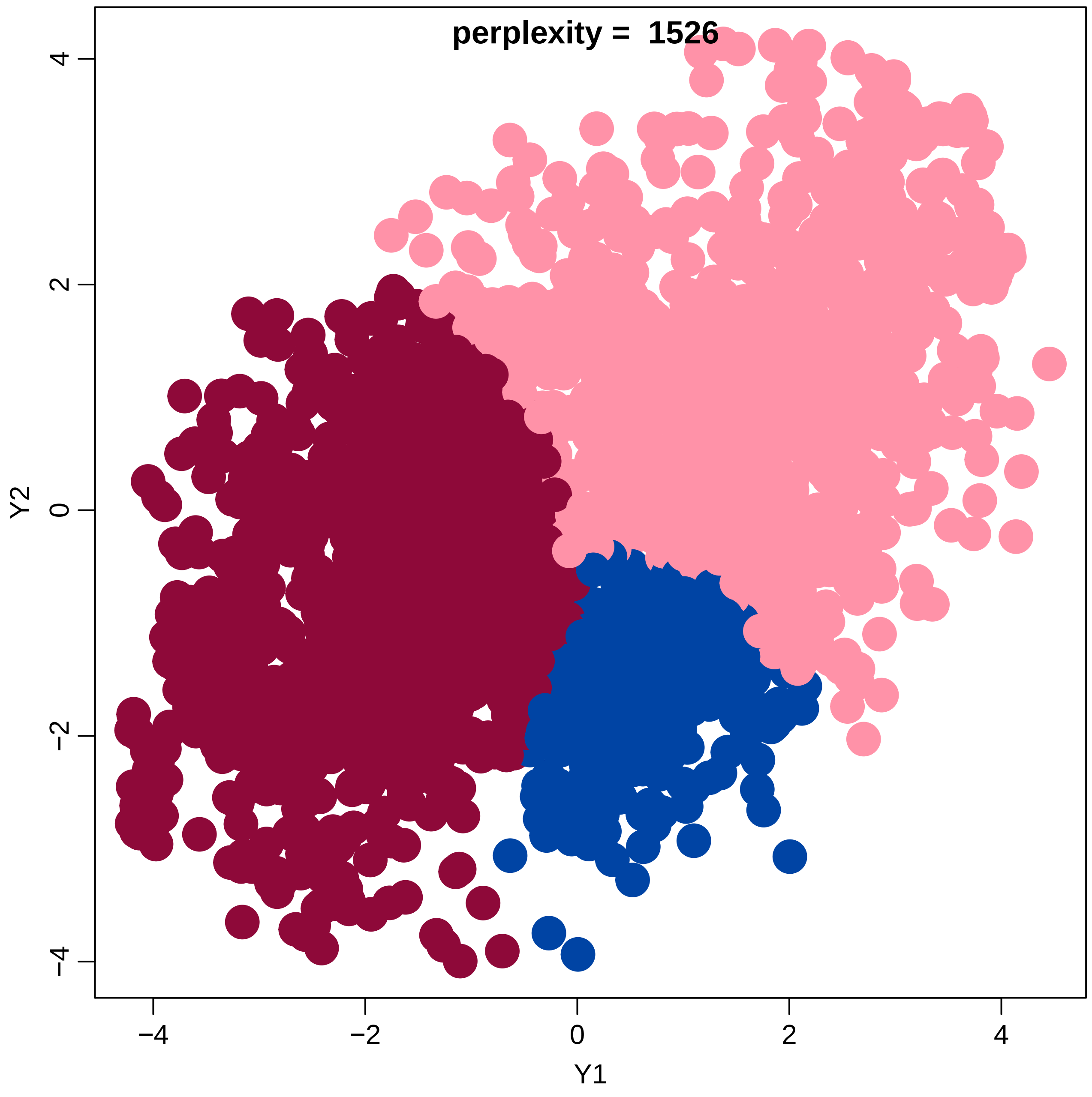
perplexity = 1526
Y2
Y1
4
2
0
−2
−4
−4
−2
0
2
4

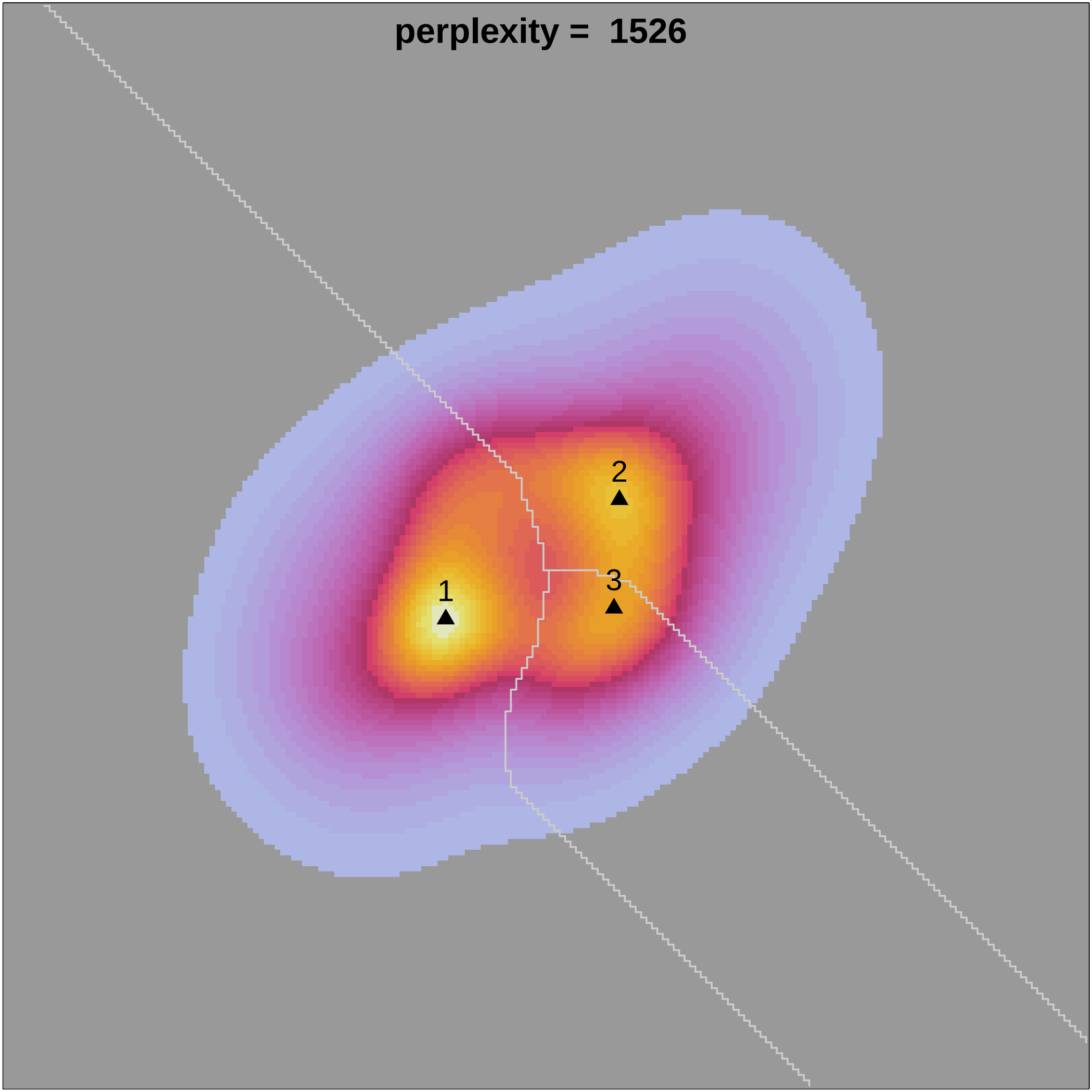
perplexity = 1526
1
2
3

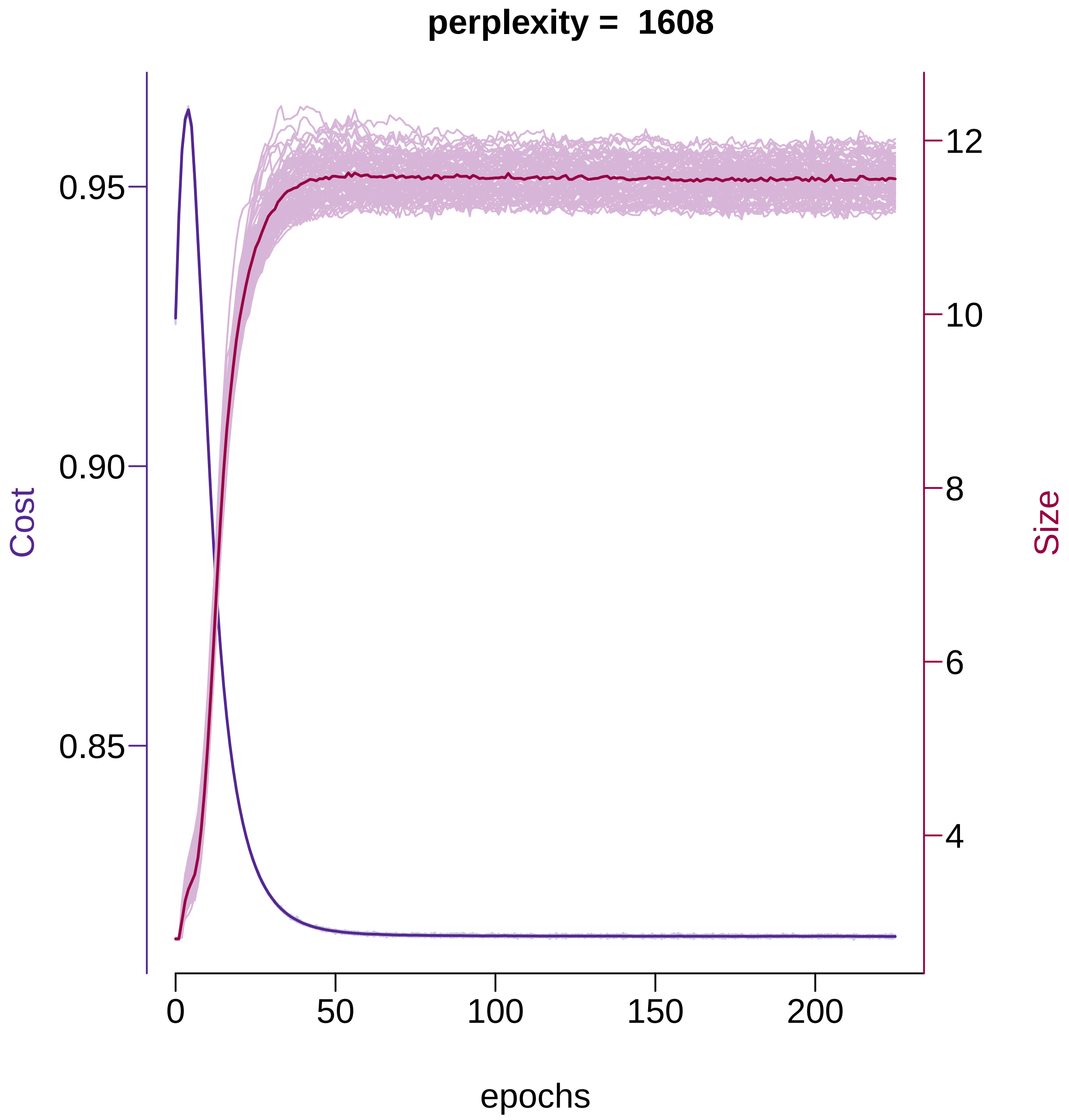

perplexity = 1608
Cost
0.95
0.90
0.85
Size
12
10
8
6
4
0
50
100
150
200
epochs

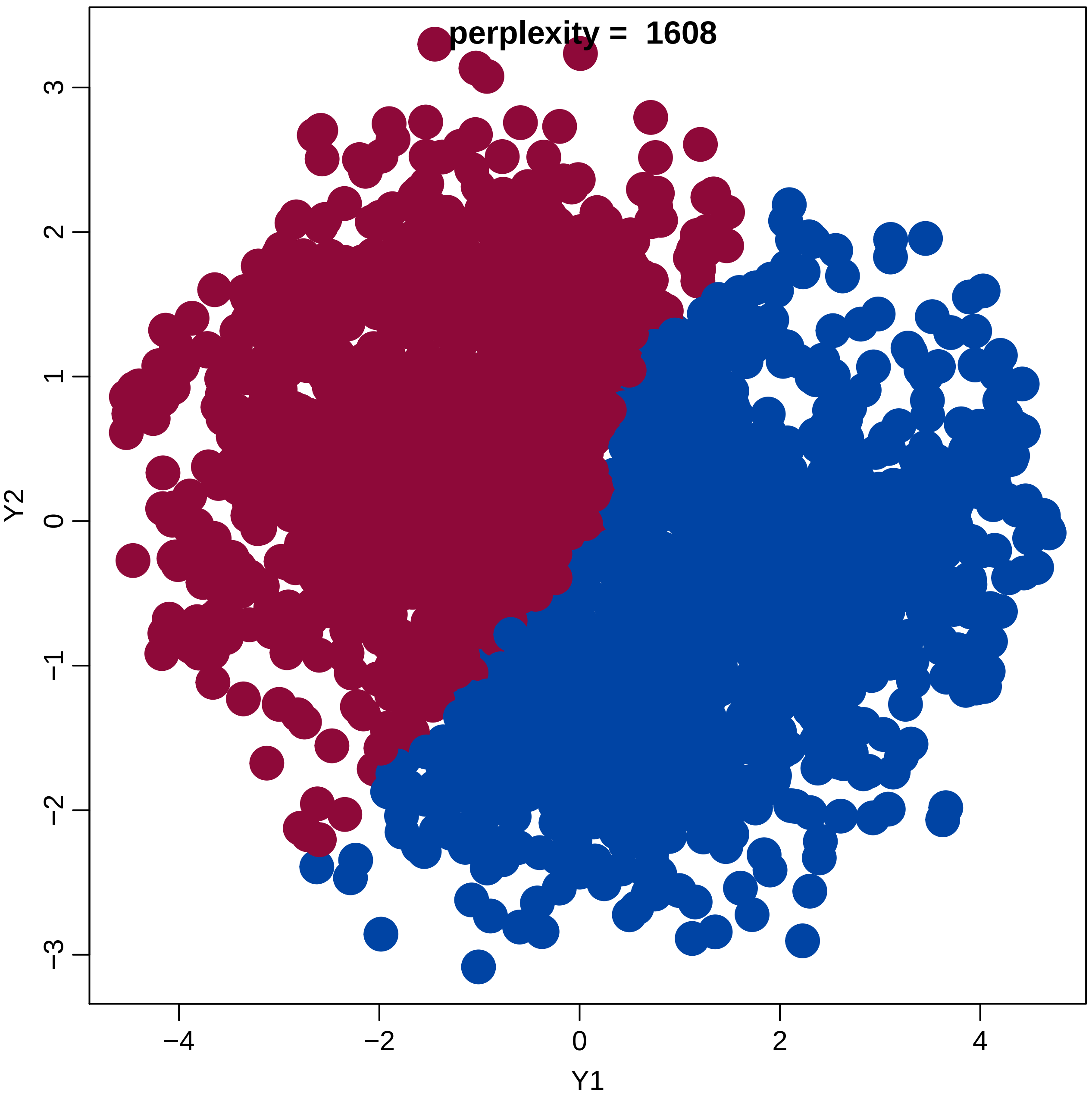
perplexity = 1608
Y2
Y1
3
2
1
0
−1
−2
−3
−4
−2
0
2
4

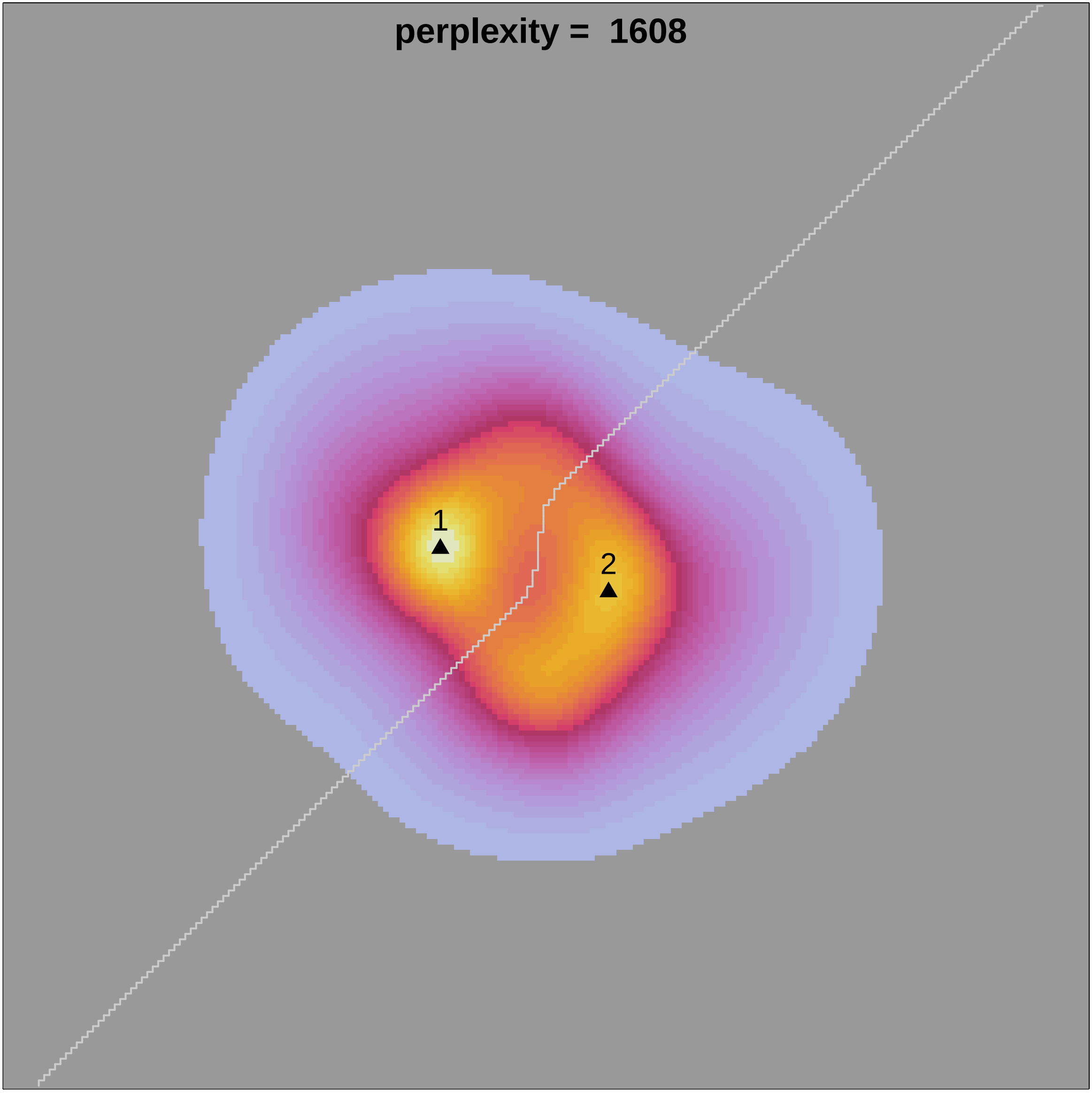
perplexity = 1608
1
2

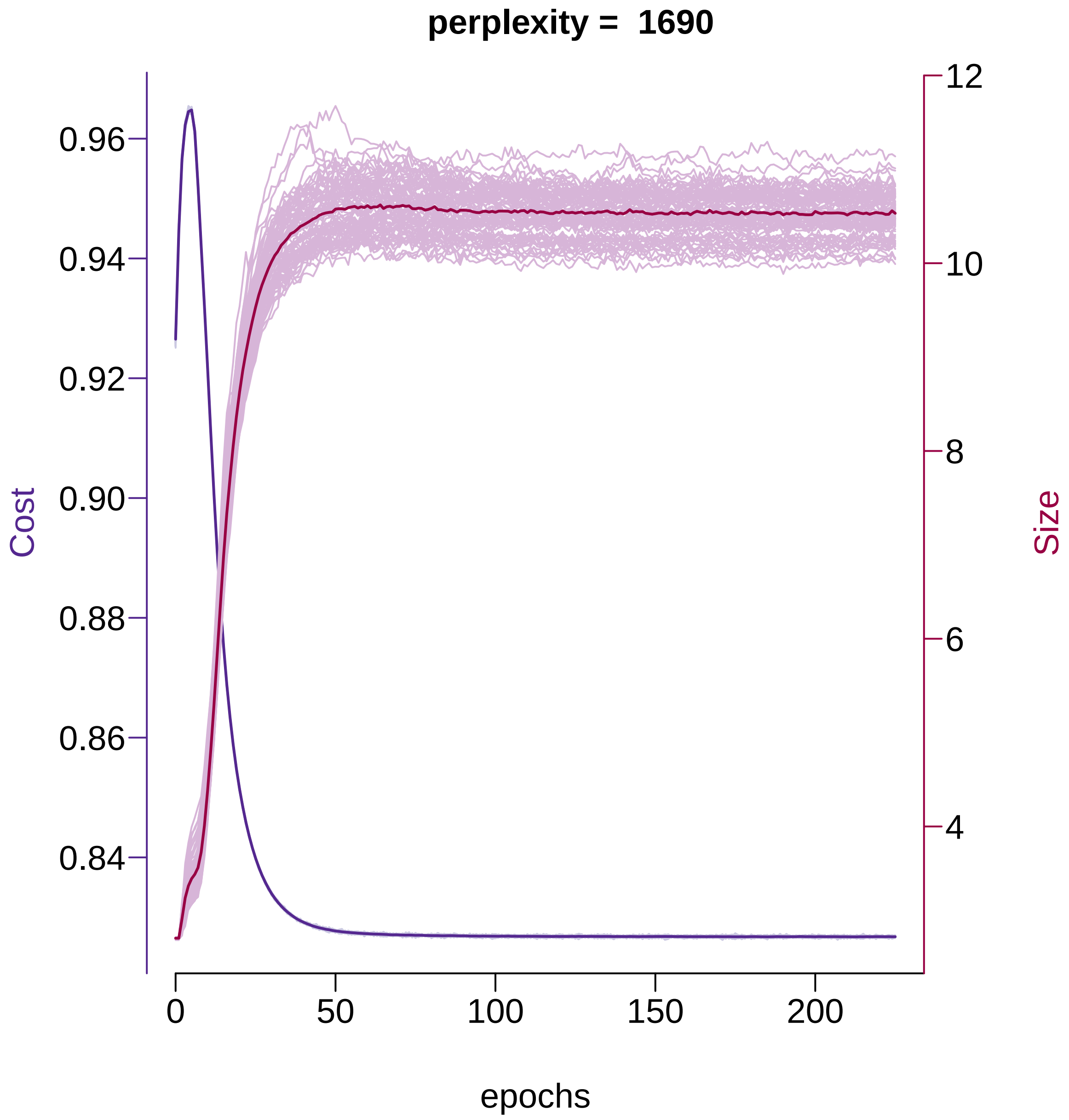
perplexity = 1690
Cost
Size
epochs
0.84
0.86
0.88
0.90
0.92
0.94
0.96
4
6
8
10
12
0
50
100
150
200

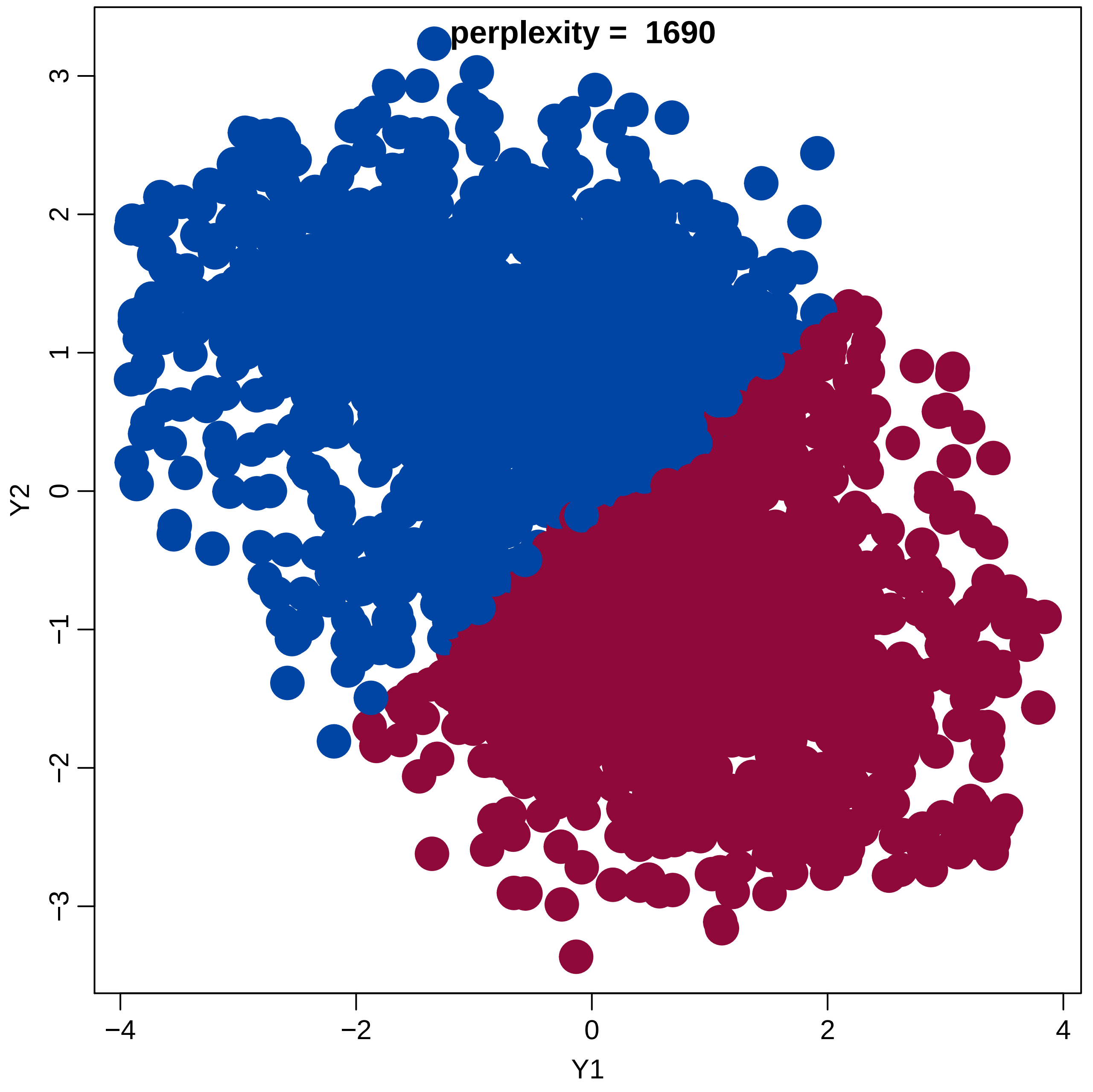
perplexity = 1690
Y1
Y2
−4
−2
0
2
4
−3
−2
−1
0
1
2
3

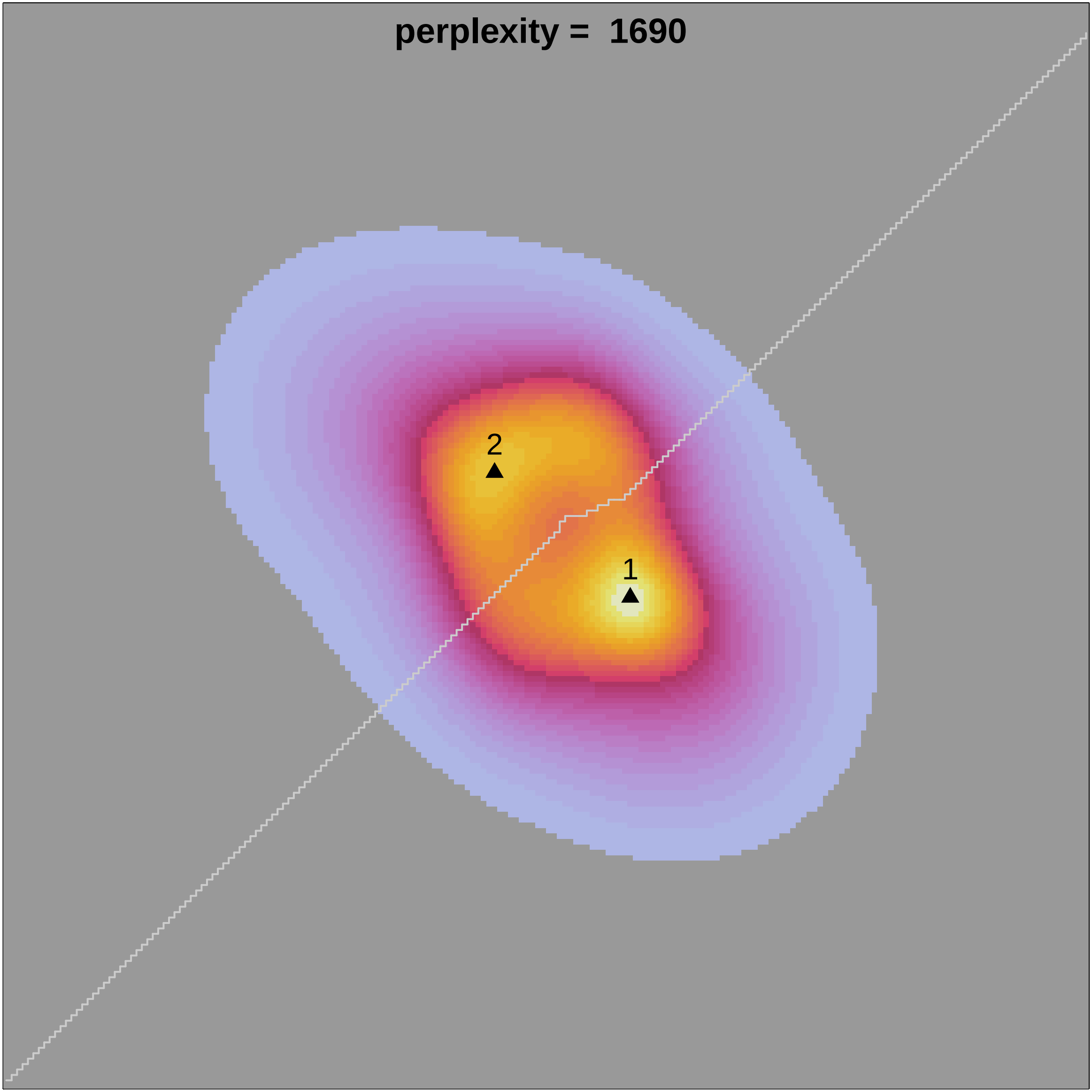
perplexity = 1690
2
1

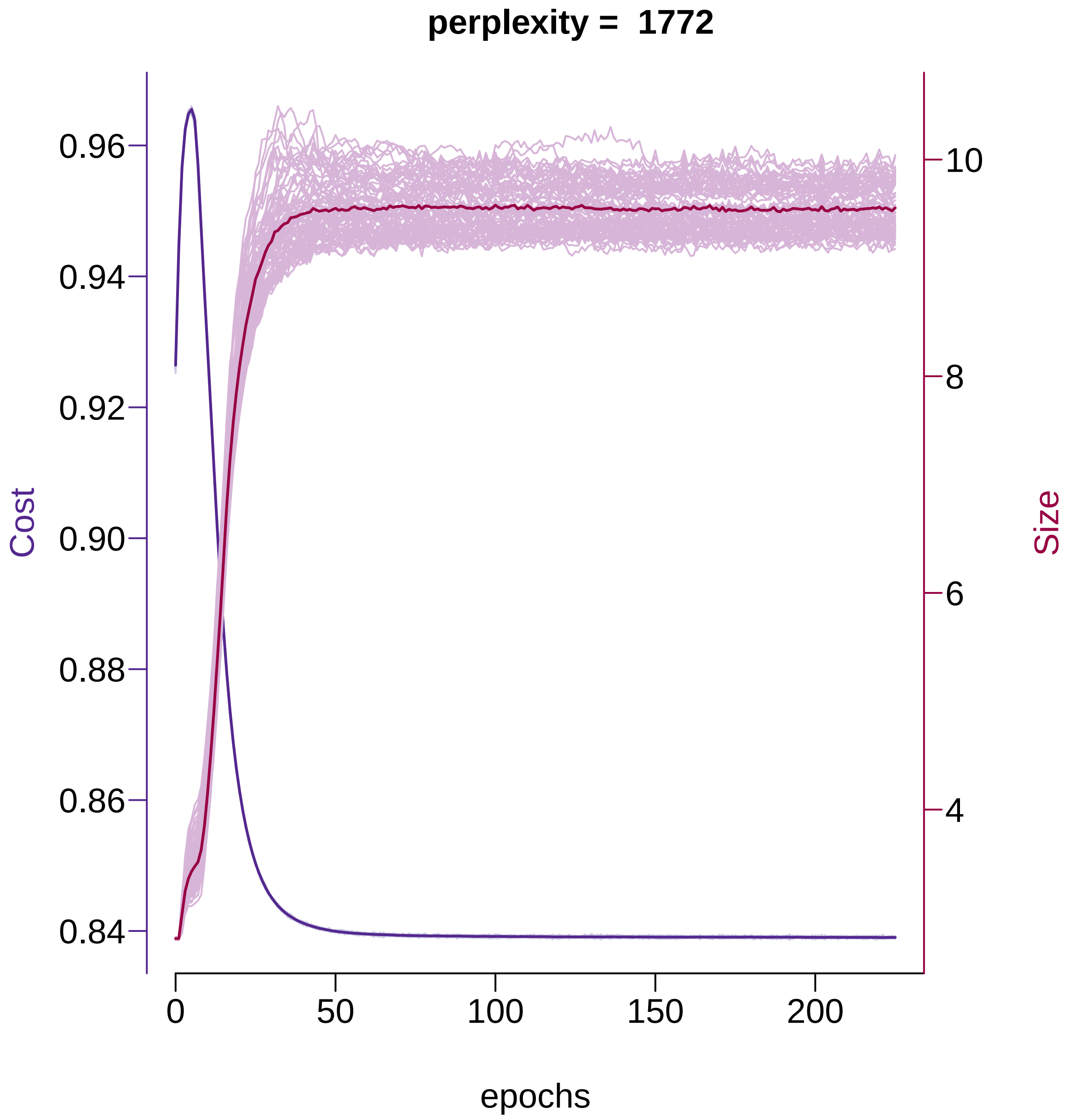
perplexity = 1772
Cost
0.96
0.94
0.92
0.90
0.88
0.86
0.84
Size
10
8
6
4
0
50
100
150
200
epochs

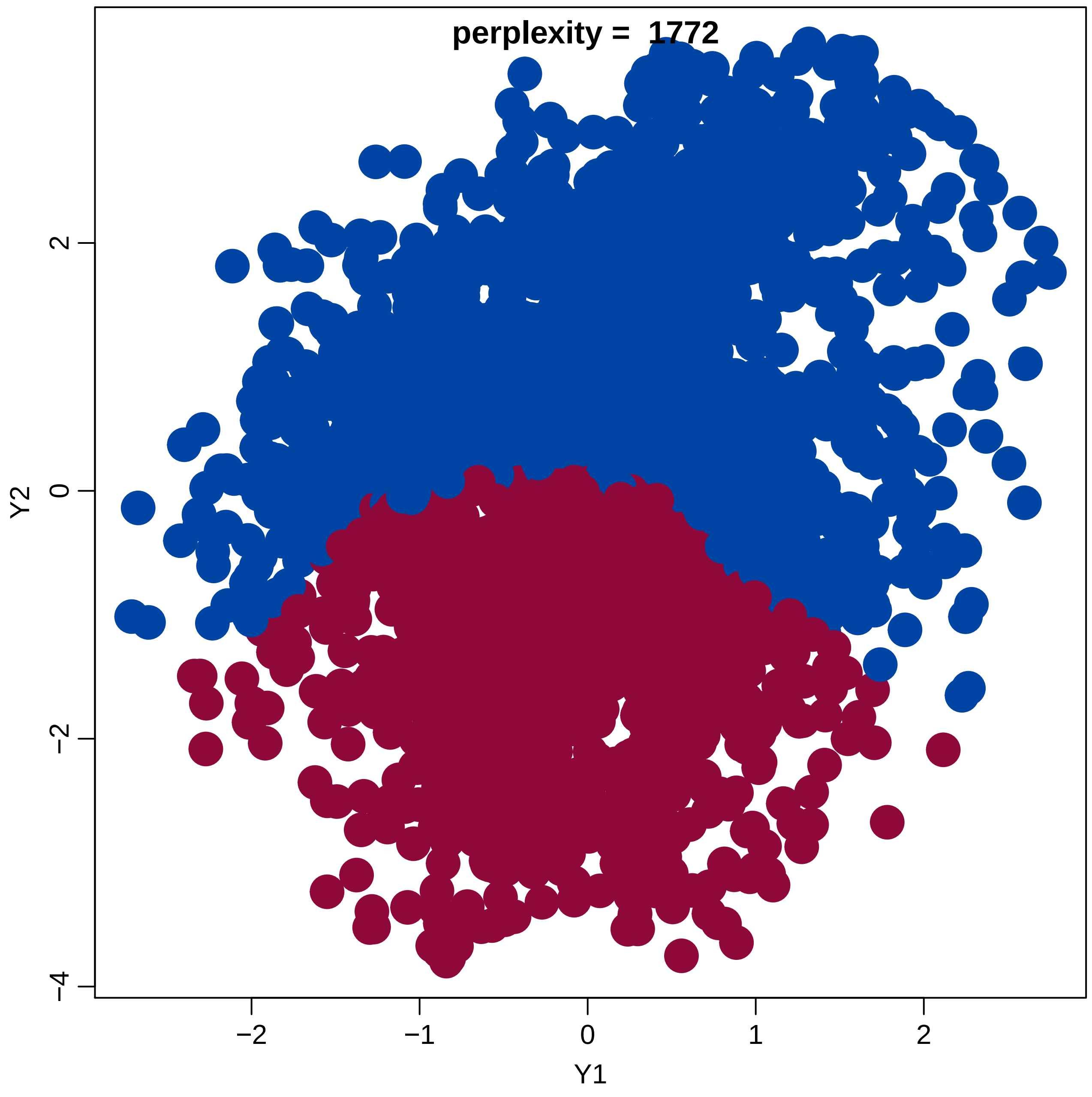
perplexity = 1772
Y2
Y1
2
0
−2
−4
−2
−1
0
1
2

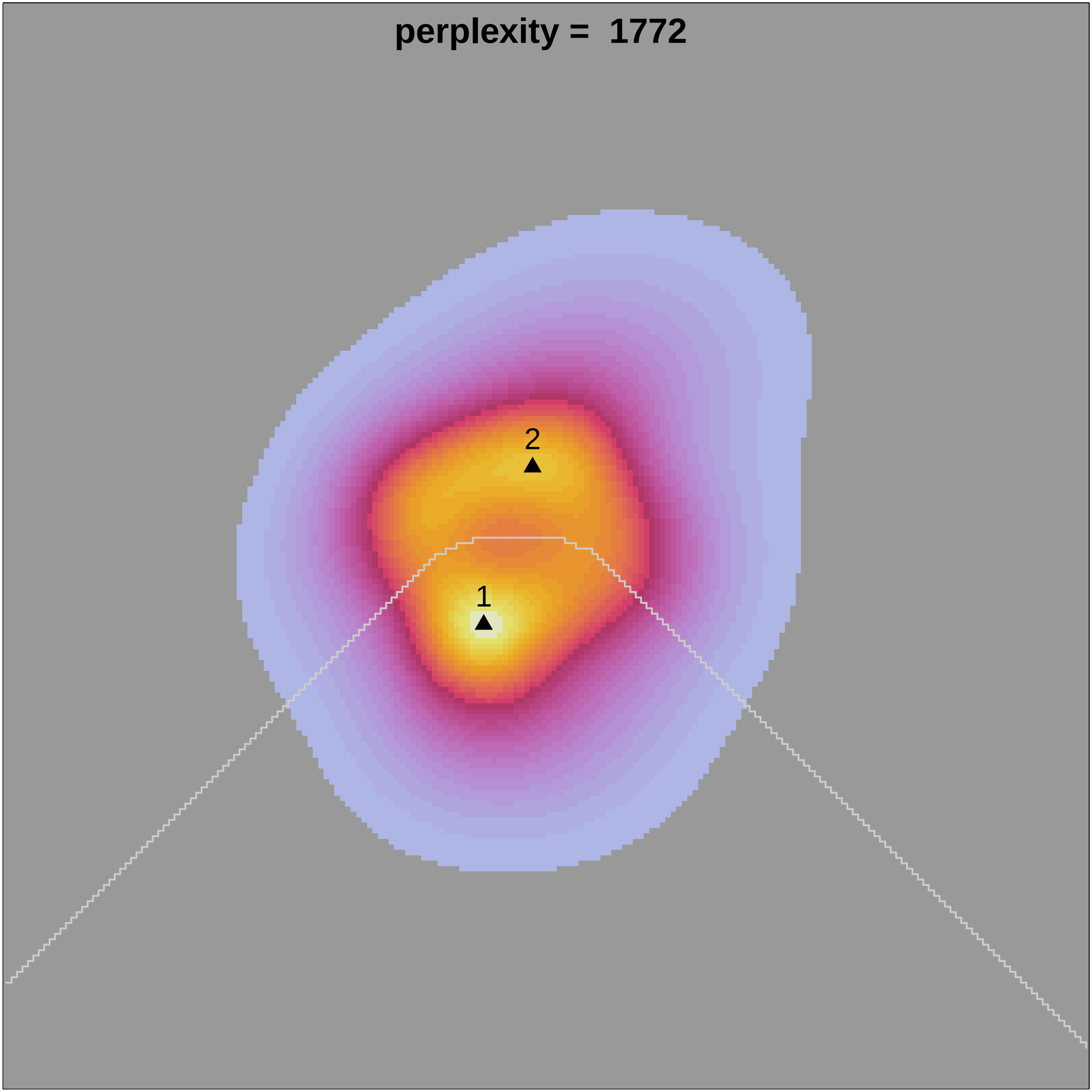

perplexity = 1772
2
1

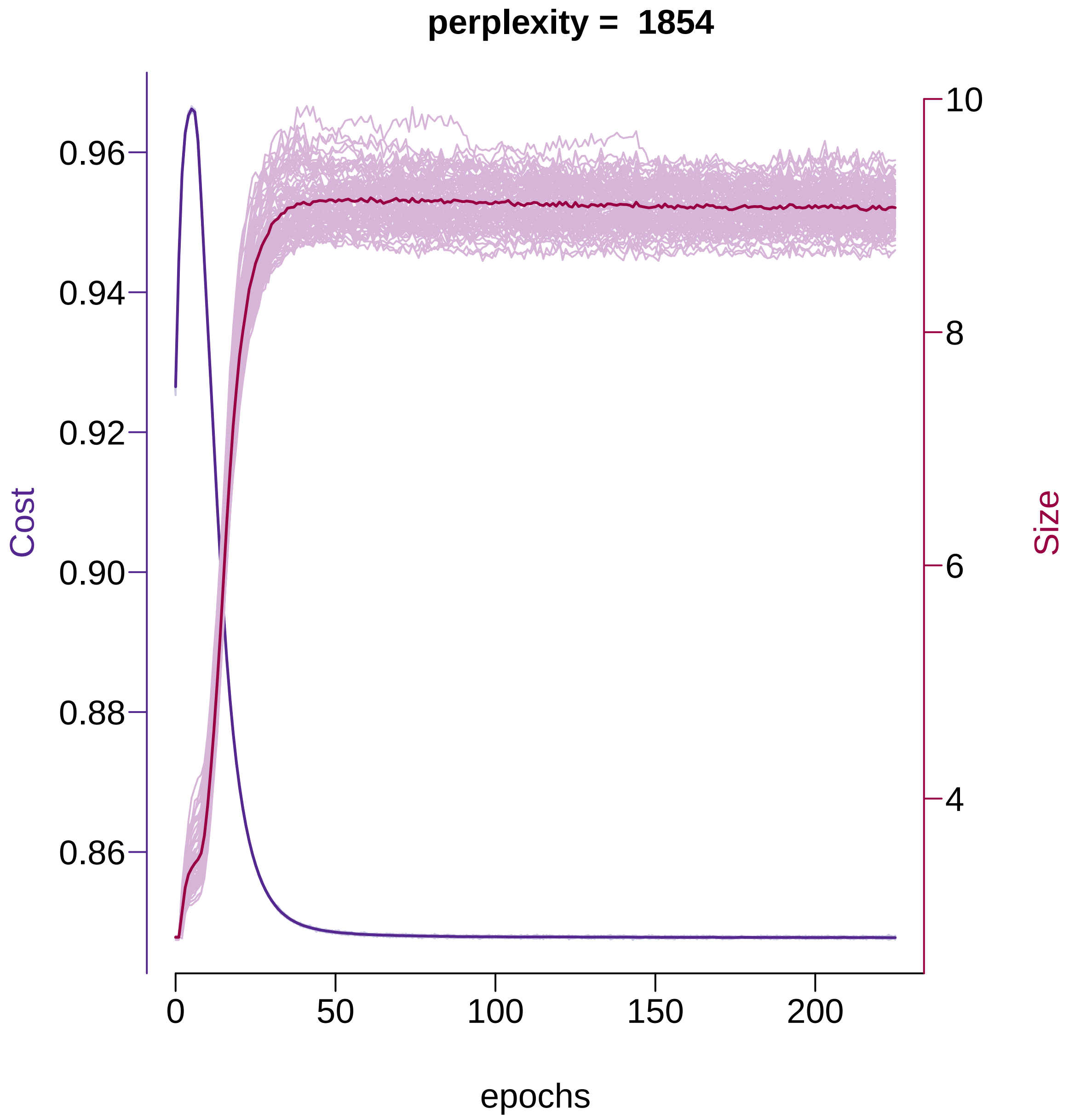
perplexity = 1854
Cost
0.96
0.94
0.92
0.90
0.88
0.86
Size
10
8
6
4
0
50
100
150
200
epochs

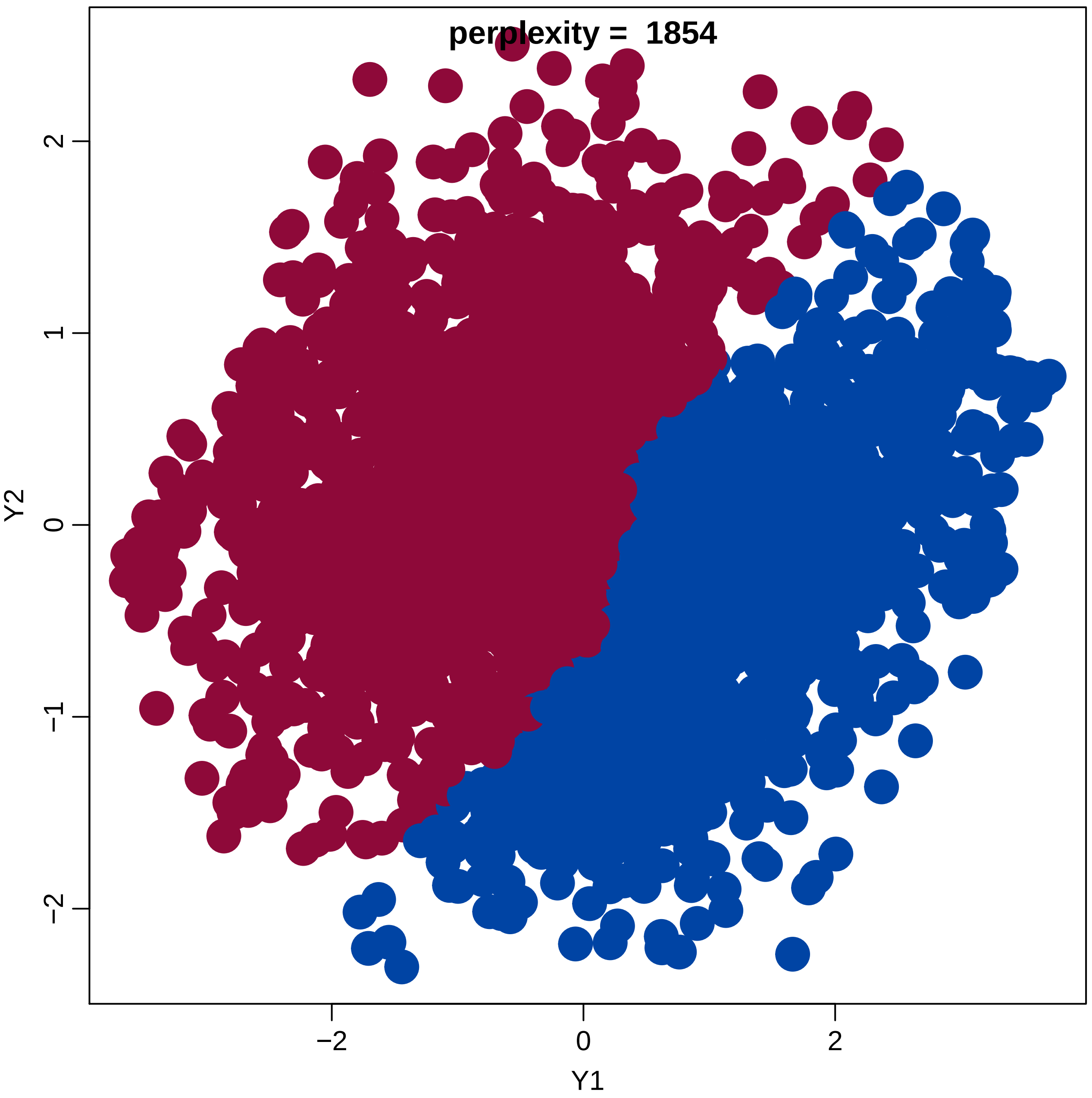
perplexity = 1854
Y2
2
1
0
−1
−2
Y1
−2
0
2

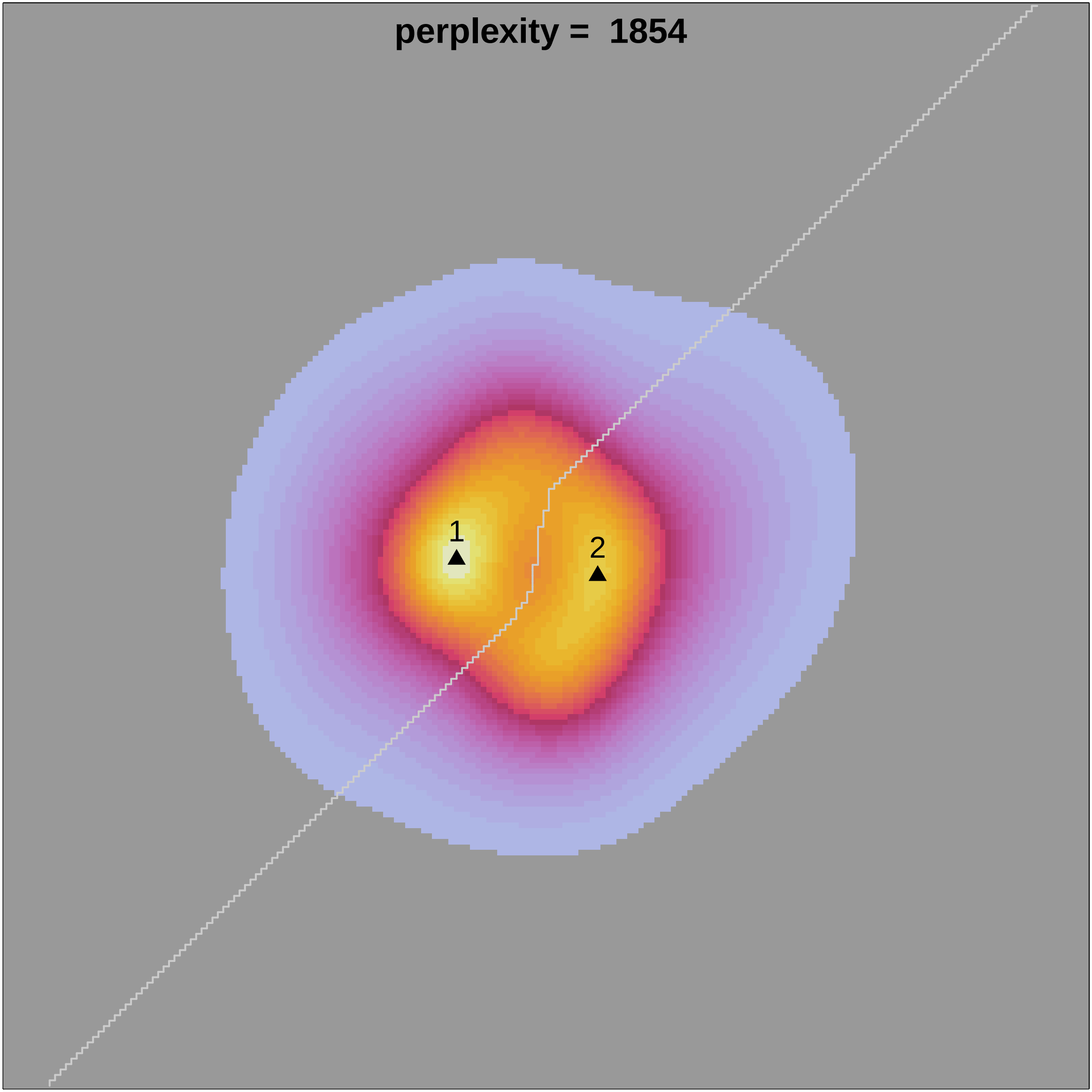
perplexity = 1854
1
2

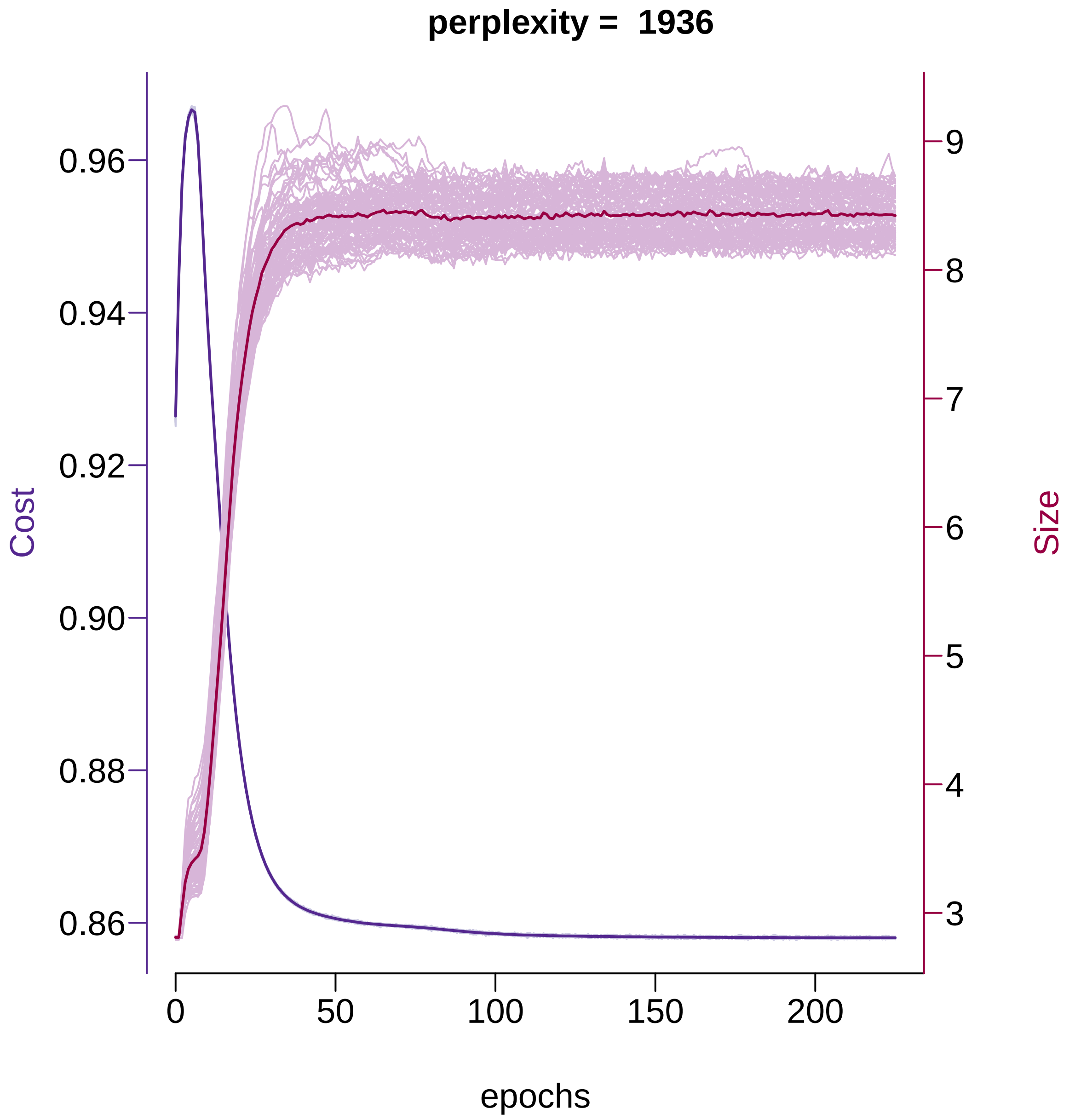
perplexity = 1936
Cost
Size
epochs
0.86
0.88
0.90
0.92
0.94
0.96
3
4
5
6
7
8
9
0
50
100
150
200

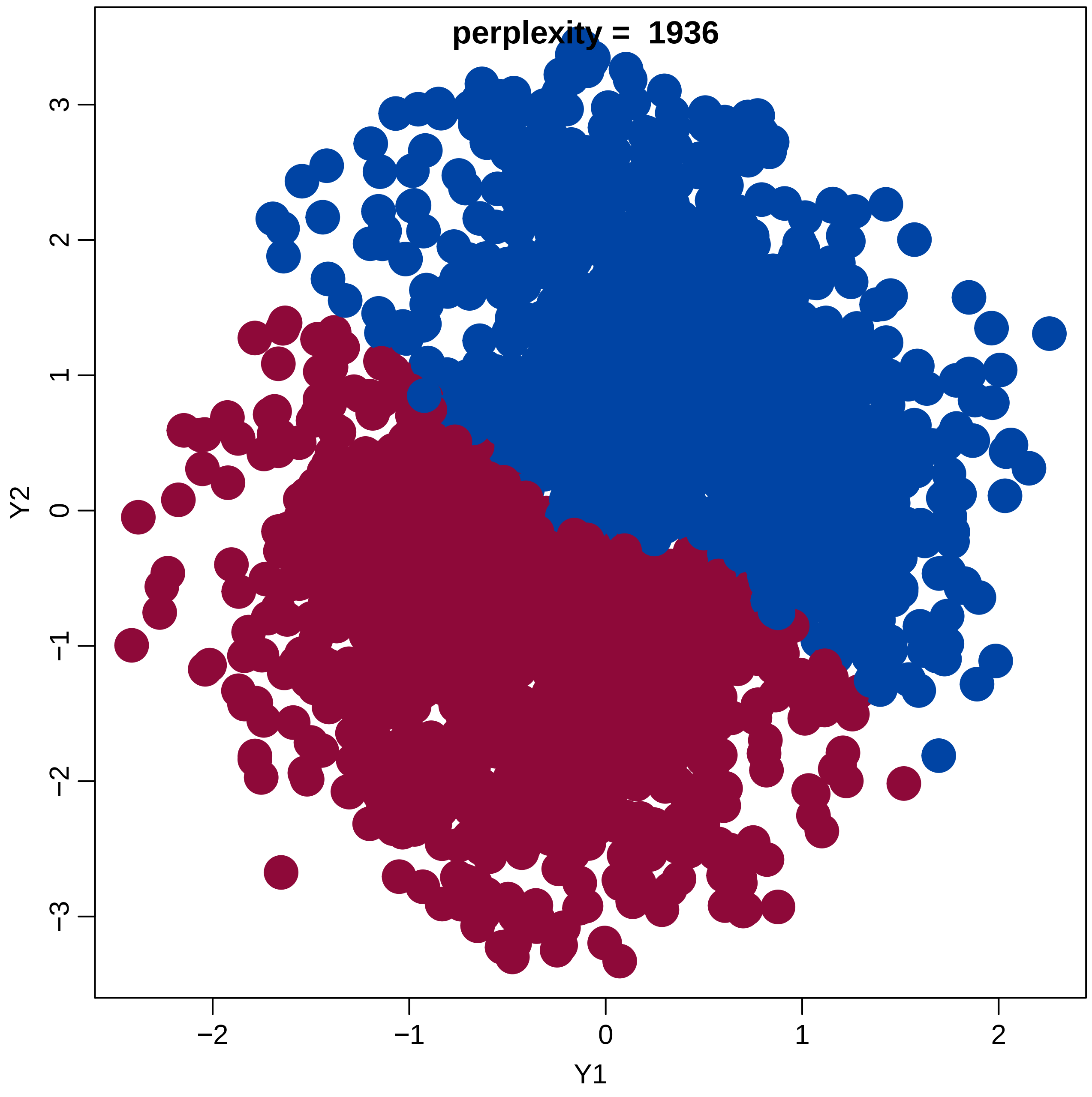

perplexity = 1936
Y2
Y1
3
2
1
0
−1
−2
−3
−2
−1
0
1
2

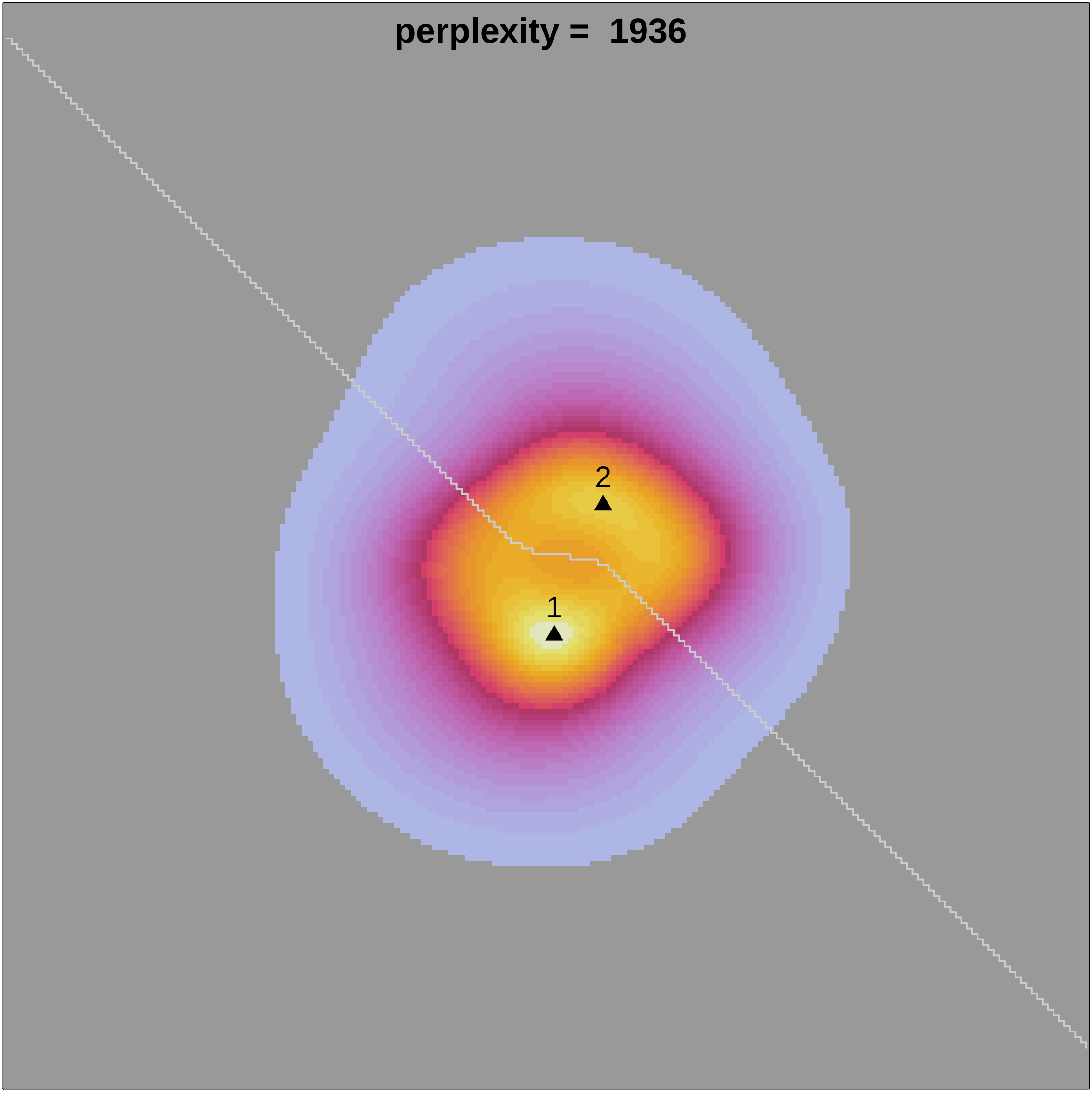
perplexity = 1936
2
1

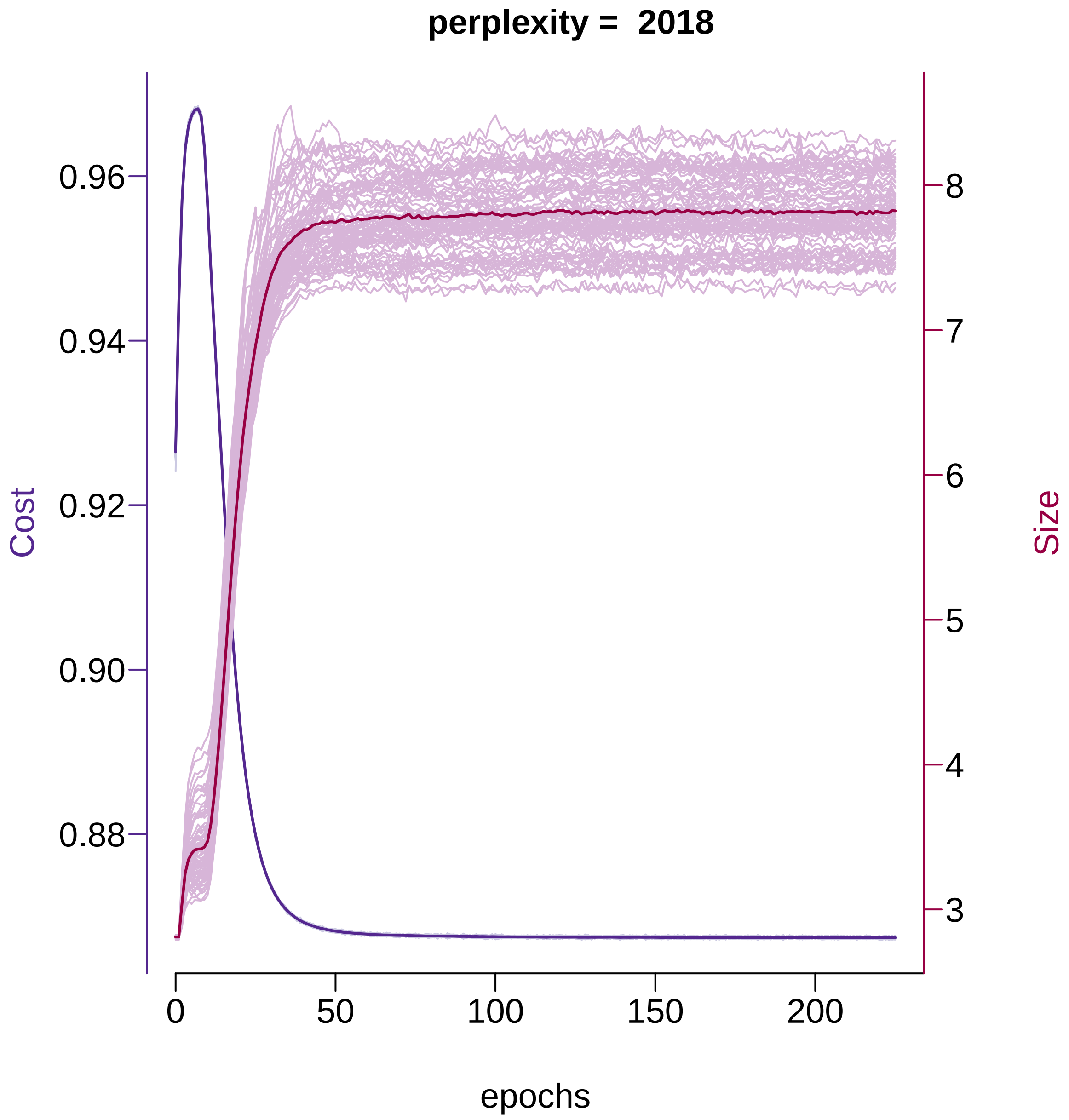

perplexity = 2018
Cost
0.96
0.94
0.92
0.90
0.88
Size
8
7
6
5
4
3
0
50
100
150
200
epochs

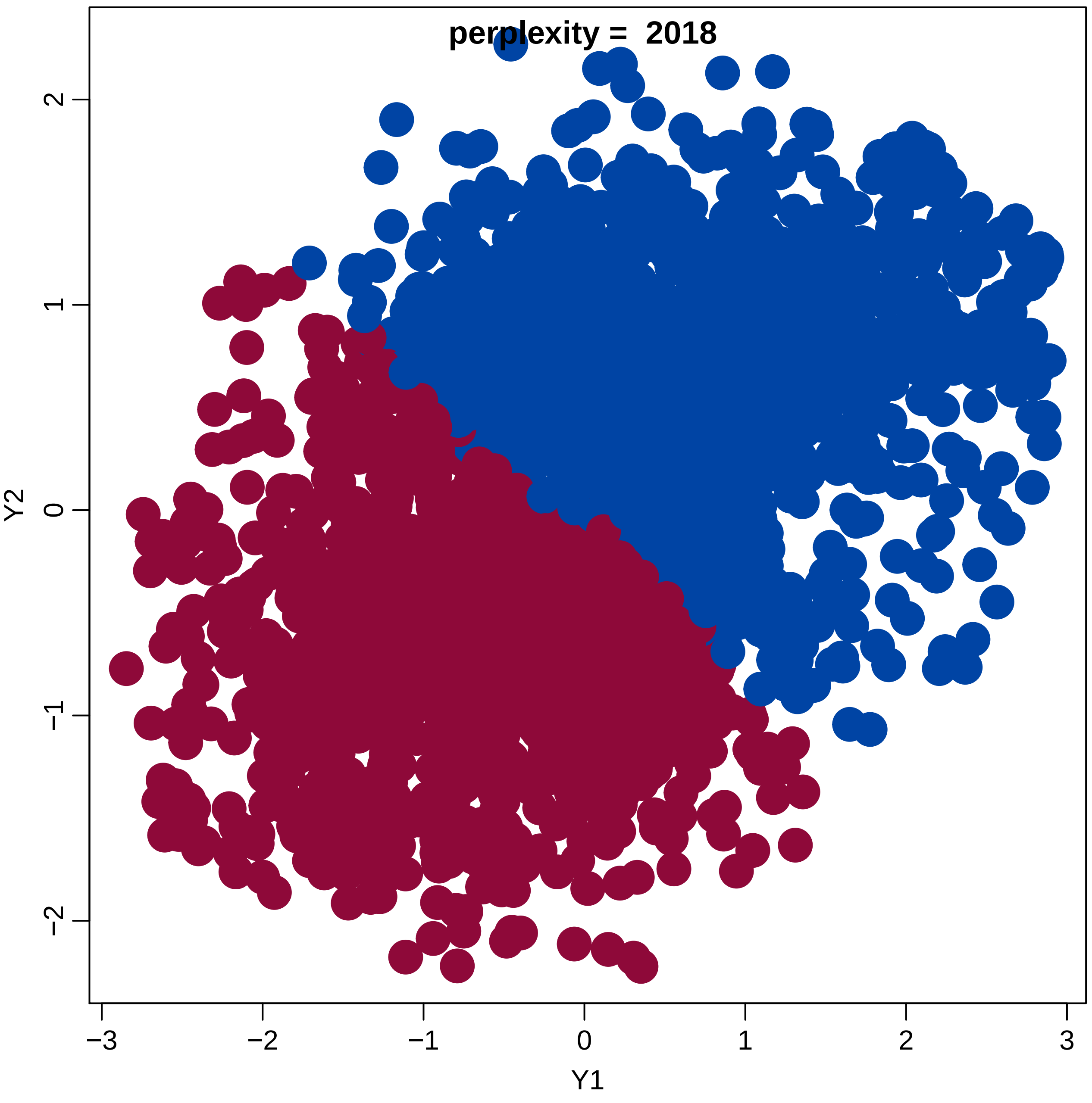

perplexity = 2018
Y2
Y1
2
1
0
−1
−2
−3
−2
−1
0
1
2
3

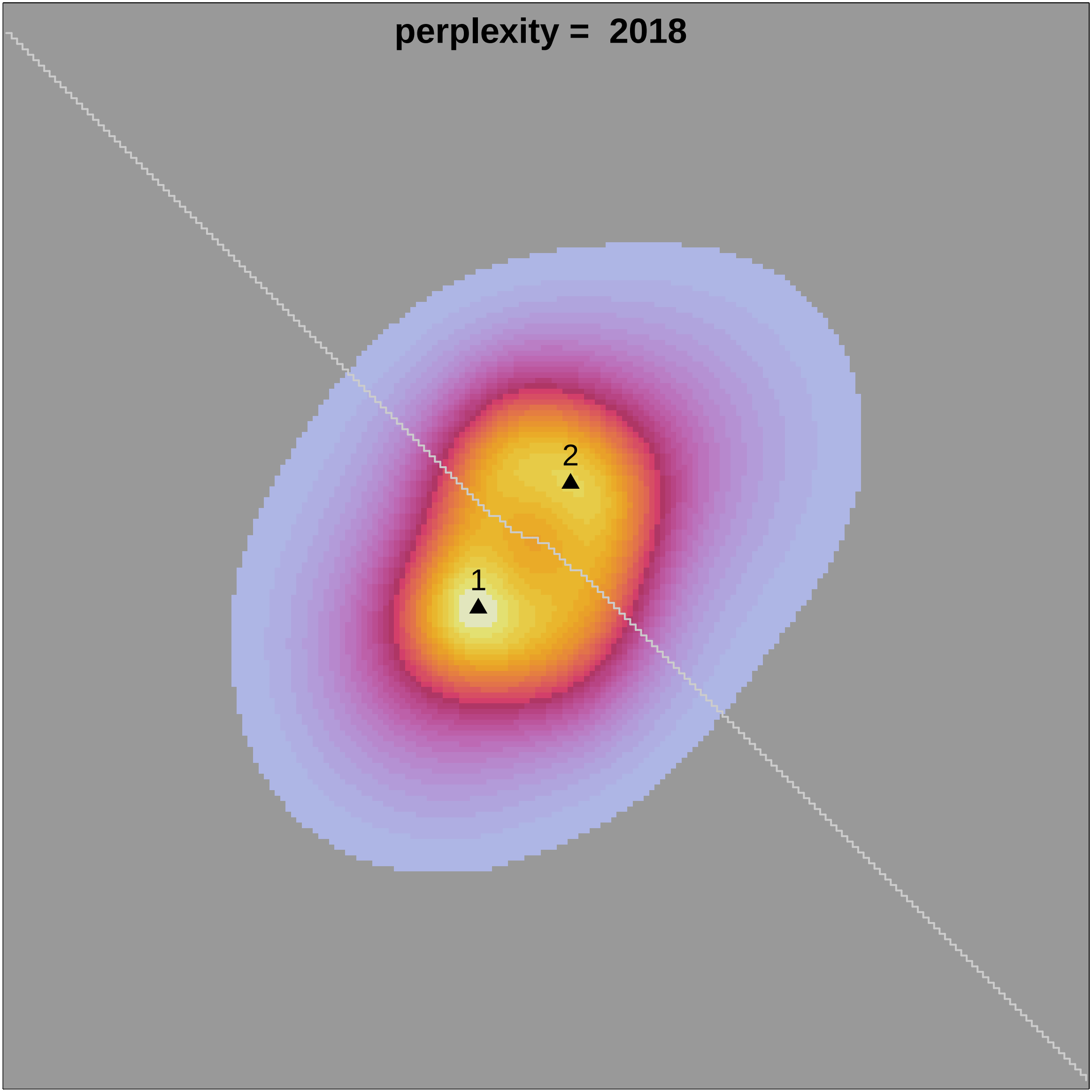
perplexity = 2018
2
1

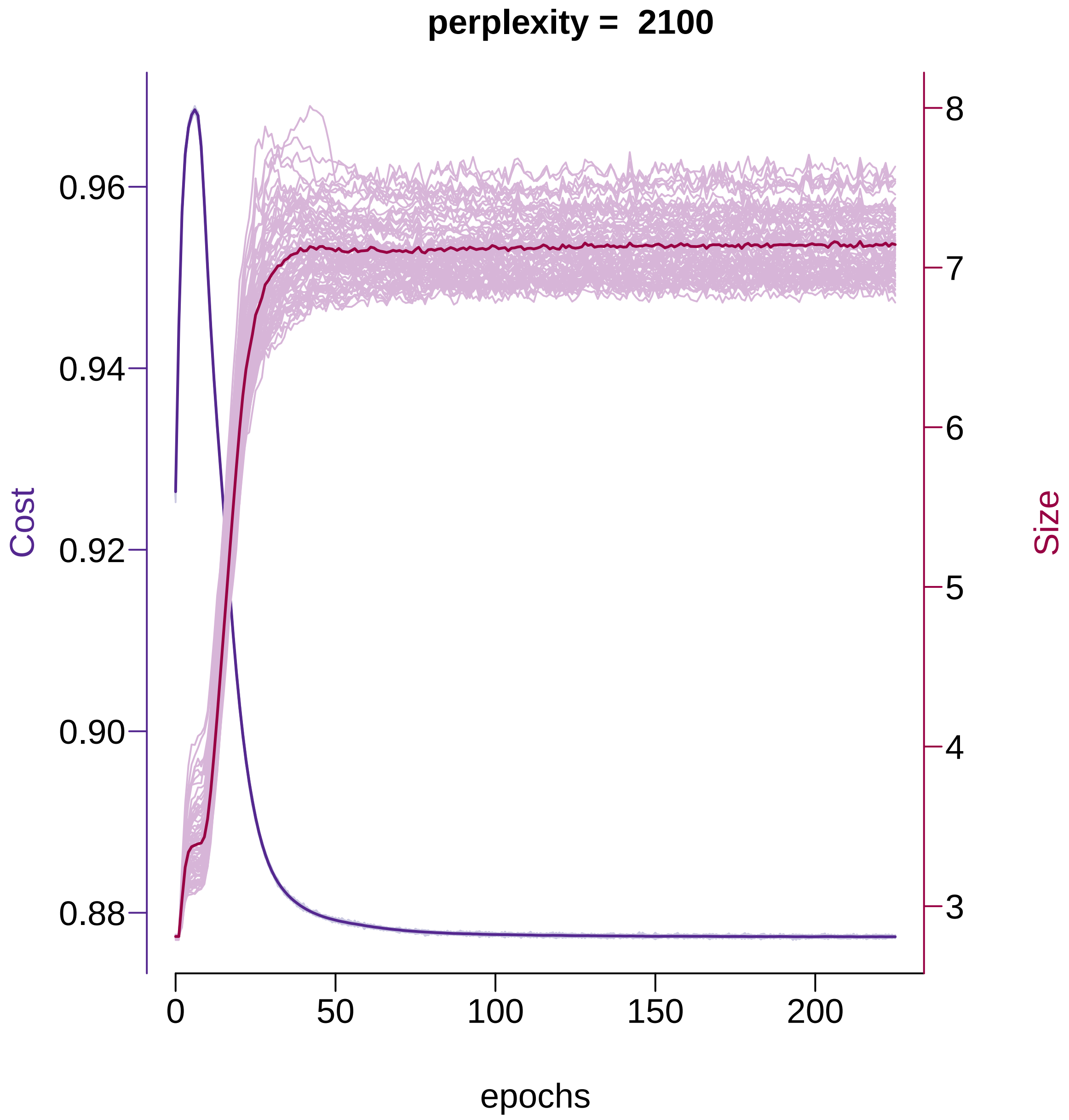
perplexity = 2100
Cost
Size
epochs
0.96
0.94
0.92
0.90
0.88
8
7
6
5
4
3
0
50
100
150
200

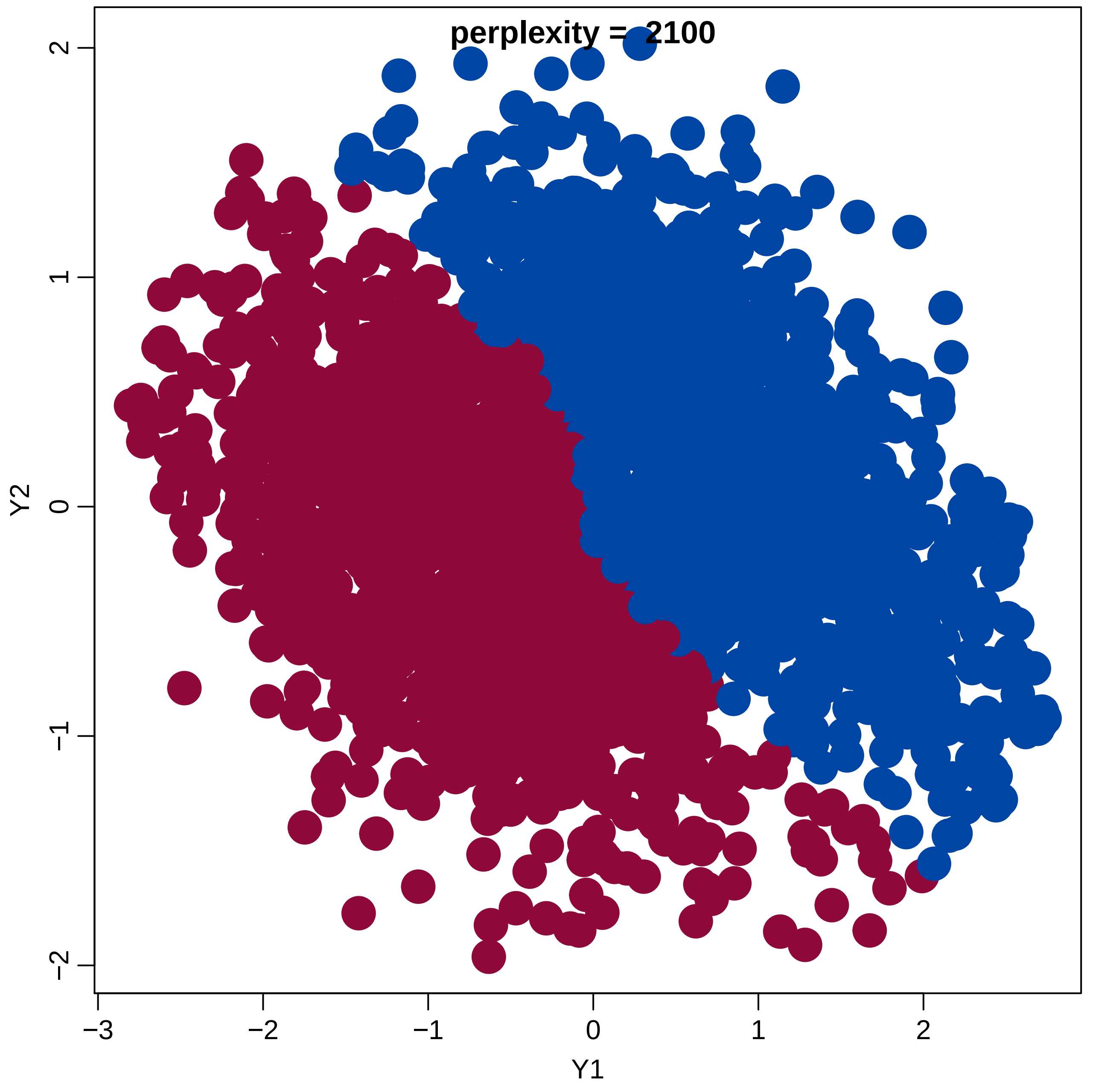
perplexity = 2100
Y2
2
1
0
−1
−2
Y1
−3
−2
−1
0
1
2

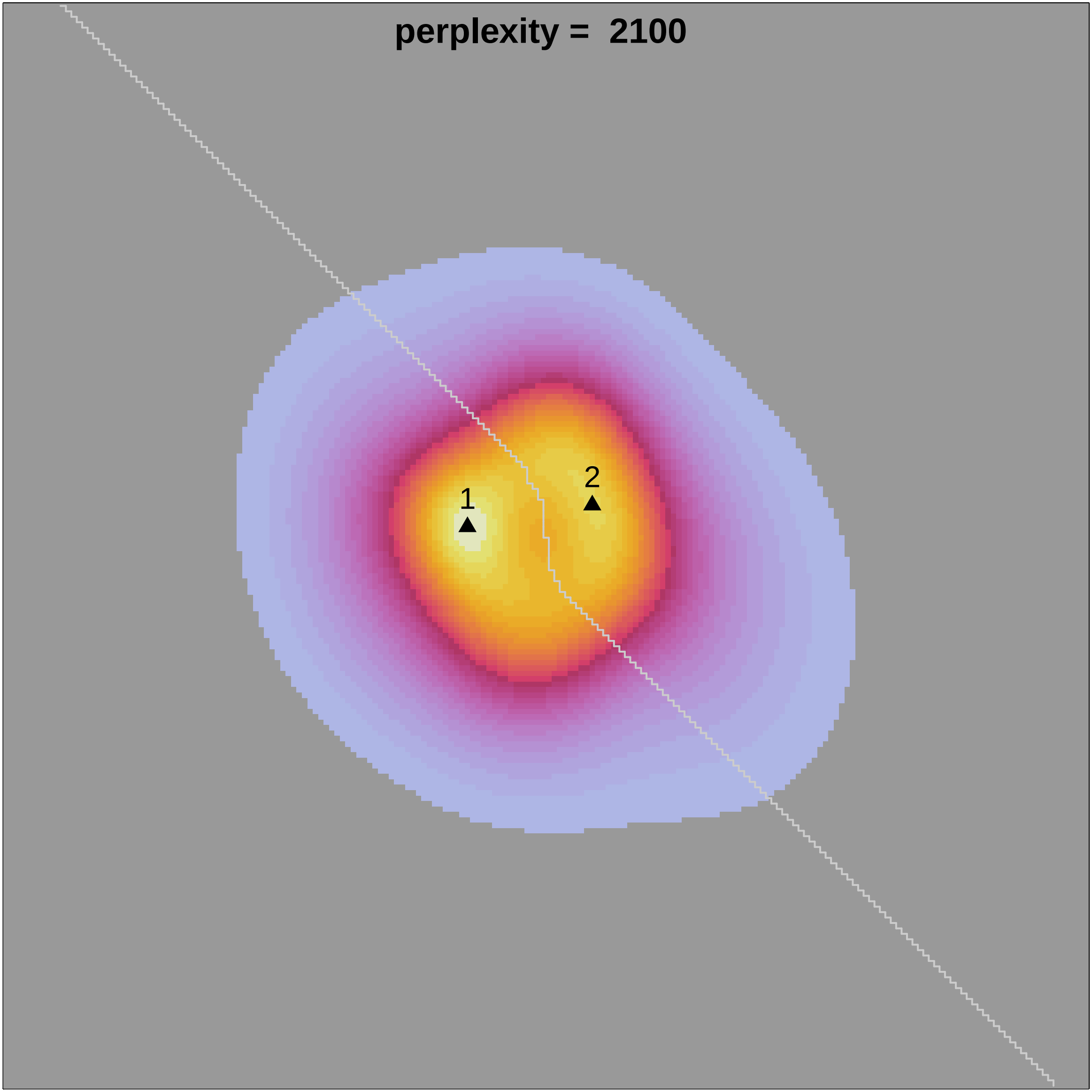
perplexity = 2100
1
2
